# How mutagenesis and laboratory conditions affect the genome of the metal-resistant bacterium *Cupriavidus metallidurans* strain CH34

**DOI:** 10.1128/jb.00121-26

**Published:** 2026-04-29

**Authors:** Cornelia Große, Viola Dreyer, Vladislava Schulz, Grit Schleuder, Thomas A. Kohl, Martin Herzberg, Dirk Dobritzsch, Matt Fuszard, Stefan Niemann, Dietrich H. Nies

**Affiliations:** 1 Martin-Luther-University Halle-Wittenberg, Institute for Biology/Microbiology 9176, Halle, Germany; 2 Molecular and Experimental Mycobacteriology, National Reference Center for Mycobacteria, Research Center Borstel, Borstel, Germany; 3 German Center for Infection Research (DZIF) Tuberculosis Unit 459706, Borstel, Germany; 4 National and WHO Supranational Reference Laboratory for Tuberculosis, Research Center Borstel, Borstel, Germany; 5 Department of Analytical Chemistry, Helmholtz Centre for Environmental Research – UFZ 28342, Leipzig, Germany; 6 Core Facility - Proteomic Mass Spectrometry, Charles Tanford Center, Martin-Luther-University Halle-Wittenberg 9176, Halle, Germany; National Institutes of Health, Bethesda, Maryland, USA

**Keywords:** genome stability, *Cupriavidus metallidurans*, Zinc

## Abstract

Insights into transition metal homeostasis of *Cupriavidus metallidurans* have relied heavily on the construction and characterization of deletion mutants. To reveal the genetic consequences of these deletions, the genomes of *C. metallidurans* strain CH34 wild type and 34 of its mutants were analyzed. The genome of *C. metallidurans* wild type remained stable when the strain was strictly kept under the appropriate conditions. Omission of selection pressure or construction of mutants, however, resulted in three different kinds of mutations. Large deletions affected genomic islands or regions in the vicinity of transposon-associated genes. Some of these large deletions could be assigned to groups of overlapping deletions associated with groups of mutants, for instance, those with deletions in metal-efflux systems. Second, single-nucleotide polymorphisms (SNPs), such as point mutations, small insertions, or deletions, with a high variant frequency, were candidates for suppressor mutations, for example, in the *corA1* or *yidC* genes. The third and novel kind of mutations comprised hypervariable regions, groups of SNPs with low variant frequencies in a small region of up to 70 base pairs that were located upstream or within genes. In the well-studied example involving the central zinc uptake regulator Zur, the hypervariable regions in the *zur* gene enabled the production of a GTP cyclohydrolase, restoring folate biosynthesis in double mutants. These data demonstrate that mutations de-stabilize the genome of *C. metallidurans,* accumulating changes that result in adaptation to the absence of the respective deleted gene, in some cases via hypervariable regions.

**IMPORTANCE:**

To determine how bacteria thrive, they are often isolated from their natural environment and maintained in a laboratory. Mutations are introduced, and these derivatives are characterized phenotypically. Understanding how bacteria and their derivatives adapt to the effects of site-directed mutations, curing of plasmids, or just the laboratory environment is important. We show here that changes in the genomes of *Cupriavidus metallidurans* mutants occurred in all instances except when the wild type was maintained under selection conditions. The secondary mutations identified may be neutral, but some may affect the outcome of subsequent experiments performed to analyze the phenotypes. Our findings indicate that all generated mutations should undergo complete genomic sequencing. The information gained may deepen our understanding of bacterial life processes.

## INTRODUCTION

*Cupriavidus metallidurans* type strain CH34 is a model system for the interaction of a bacterium with a mixture of transition metal cations in mesophilic environments (1–3). The beta-proteobacterium can be found in metal-contaminated environments, including zinc deserts and auriferous soils (4–7). Its genome is composed of a chromosome, a chromid, which is a chromosome-like but RepA-controlled replicon, and two large plasmids, pMOL28 and pMOL30, both of which are central for metal resistance of this bacterium. All the replicons contain a variety of genomic islands, large sets of genes acquired by horizontal gene transfer, some of which harbor metal resistance determinants in addition to those located on the plasmids (8, 9). The genome of *C. metallidurans* has been sequenced (10), the transcriptome has been analyzed by gene arrays and RNA-seq (11–14), and the proteome has also been investigated (11, 15–17).

Central to the ability of *C. metallidurans* to handle mixtures of essential-but-toxic and toxic-only transition metal cations is a flow equilibrium of zinc and other metal cations (18–21), which results from rather unspecific metal cation import by at least 10 transport systems (22, 23) plus efflux by P-type ATPases (TC3.A.3, transporter classification system [24, 25]), CDF proteins (TC2.A.4), and other inner membrane proteins (26–28). Additionally, large hetero-multimeric transenvelope efflux complexes, such as CzcCBA and CnrCBA encoded on plasmid pMOL30 and pMOL28, respectively, remove cations from the periplasm to the outside and mediate resistance to high (low mM) concentrations of metal ions (3, 29). *C. metallidurans* is also able to survive zinc starvation conditions at very low (nM) concentrations of the essential cation and can substitute some of the zinc with cobalt ions (30, 31). This ability is mediated by the components of the Zur regulon, mainly the zinc importer ZupT of the ZIP protein family (TC2.A.5) plus three metal-binding GTPases designated CobW1–CobW3 (31–36).

**Fig 1:**
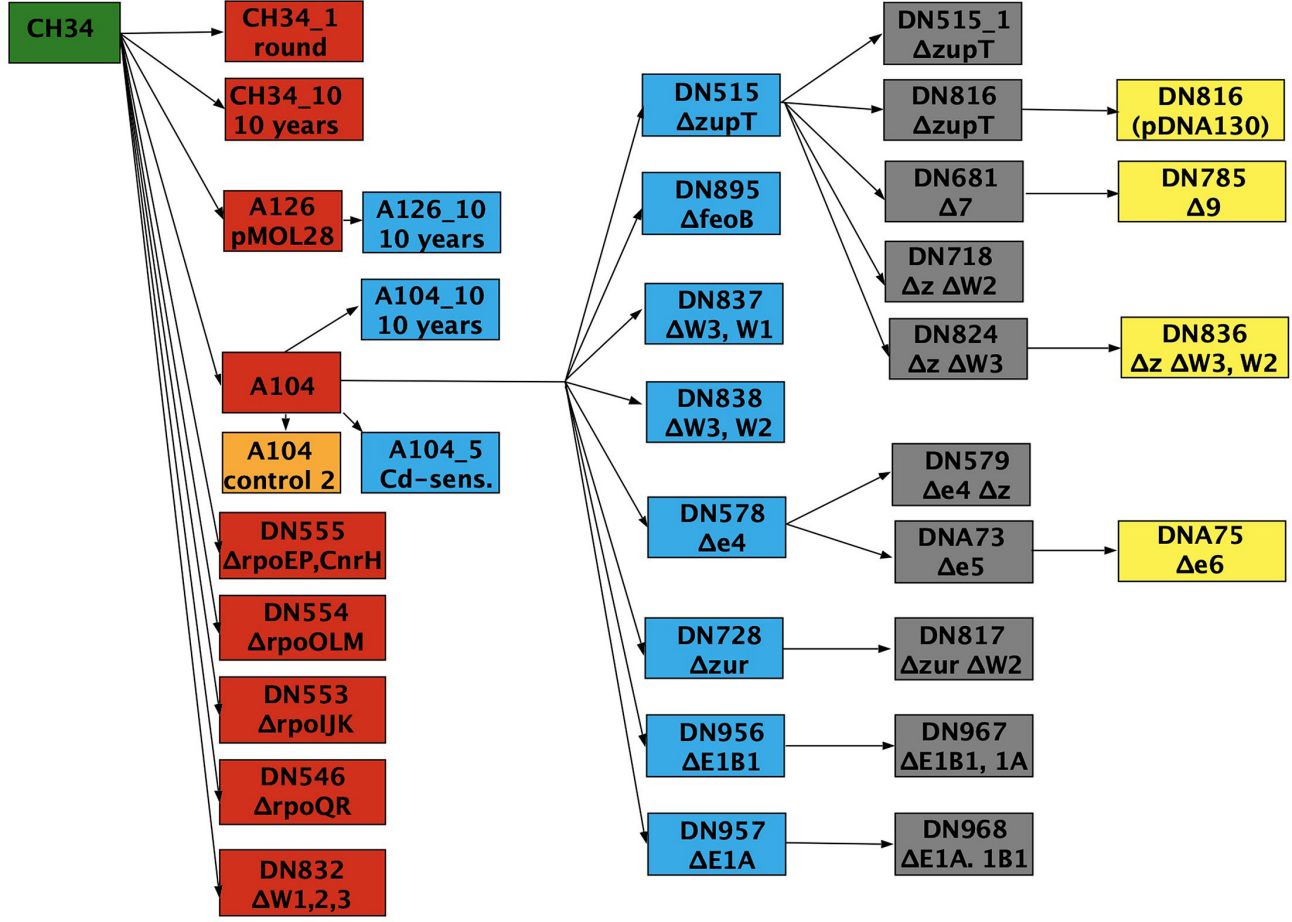
Overview of the mutant strains that were sequenced. The arrows lead from the parent to the respective descendant strain: *C. metallidurans* CH34 wild type (green), first level (red; re-sequenced biological repeat of the plasmid-free strain AE104, orange), second level (blue), third level (gray), and fourth level (yellow). The same colors as in Fig. 10 and the supplementary Excel sheets are used. AE126 contains only plasmid pMOL28. Strains DN546 to DN555 are sigma factor deletion strains with the deleted genes for the sigma factors indicated. Some names for deletions were abbreviated: ∆z, ∆*zupT*; ∆W, ∆*cobW; ∆*E1B1*, ∆folE_IB1; and ∆*E1A*, ∆folE_IA*. Efflux mutants ∆e4, ∆e5, and ∆e6 and uptake mutants ∆7 and ∆9 are explained in the text. Please note that strain AE104 appears twice as another control (orange).

Understanding metal homeostasis in *C. metallidurans* relied heavily on the creation and investigation of deletion mutants (Fig. 1). Initially, the two plasmids were cured in 1982 (1). First, only one marker-free deletion plus one interruption could be introduced into the genome of the bacterium (37). Subsequently, the number of deletions could be increased to eight marker-free deletions, plus one interruption (22, 23, 28). Some of these constructed deletions were accompanied by unwanted ones, which were often located in genomic islands (13).

The effect of certain constructed deletions in a bacterium may result in a partial suppression by secondary mutations that allow the mutant cells to survive and grow. An example comes from *ssrA* (38). Together with the RNA chaperone SmpB, *ssrA* binds to stalled translating ribosomes that do not possess a complete codon at the A-site due to an incomplete mRNA. The *ssrA* is also called “tmRNA” because it functions first as tRNA and rescues the ribosome. Subsequently, *ssrA* functions also as an mRNA so that translation can proceed and be terminated at a stop codon within the mRNA part of *ssrA*. The resulting protein contains an *ssrA*-encoded peptide tag at the C-terminus, which labels it for degradation. Some bacteria such as *Escherichia coli* contain an additional pathway to rescue ribosomes stalled by incomplete codons at the A-site, ArfA (39). A ∆*ssrA ∆arfA* double mutation in *E. coli* is synthetically lethal, but overexpression of the *yaeJ* gene could suppress the lethal effect of the ∆*ssrA ∆arfA* double mutation (40). In this way, a third pathway able to rescue stalled ribosomes with an incomplete codon at the A-site was identified. Finding suppressions by secondary mutations and studying their effect may lead to a deeper understanding of the full consequences of the introduced mutation.

Zinc is an essential element (41), and deletion of the *zupT* gene for the main zinc importer in *C. metallidurans* already leads to a pleiotropic effect, including the accumulation of misfolded RpoC beta-prime subunit of the RNA polymerase (35). Multiple deletions in other genes connected to zinc homeostasis might have led to synthetic lethal effects that were not recognized because suppressor mutations might have been selected. Characterization of such "suppression" may provide deeper insights into zinc homeostasis or multiple metal homeostasis in general. Here, we characterized in detail the genomes of *C. metallidurans* strain CH34 wild type and 34 of its mutants (Fig. 1). Twelve mutants carry deletions in genomic islands, which have already been published (13). We asked the question of whether additional mutations with a potential to affect metal ion homeostasis might have appeared in these 12 mutant strains, along with 22 additional mutants and the wild type. We show that the genome of parental strain CH34 stays stable, but only when kept strictly under selection pressure. Loss of the plasmids, cultivation on mineral salts medium without metal stress or constructed mutations, however, resulted in additional secondary mutations: large deletions; single-nucleotide polymorphisms (SNPs) with a high variant frequency, such as point mutations, small deletions, or insertions (InDels); or hypervariable regions, upstream, or within genes. Hypervariable regions are clusters of low-frequency SNPs within a small region of up to 70 base pairs and seem to be a novel type of mechanism to adapt to strong selection pressure, which mostly occurs only in multiple deletion strains of *C. metallidurans*. In one case, in which a link between the zinc uptake regulator Zur and enzymes involved in folate biosynthesis was identified, the suppression was studied in detail to demonstrate the connection between a hypervariable region and the resulting phenotype.

## RESULTS

### Overall approach

The genomic sequences of *C. metallidurans* CH34 and its 34 mutants (Fig. 1) were assembled into the genome of CH34 wild type (10). Large, newly identified deletions were found in 23 mutant strains in addition to those already published in 12 strains, the wild type, and 11 derivatives (13) (Fig. 10, details are provided in the supplement). SNPs and regions of low coverage (smaller than mean coverage minus 2-fold deviation) were identified in all annotated genomes. Changes with a SNP frequency greater than 90% were defined as “almost certain mutations” or simply “mutations,” those between 50% and 90% as “possible mutations,” but only if they were (i) outside the regions of low coverage; (ii) not at the edge of large deletions; and (iii) not assigned to genes having multiple paralogs, which may indicate a possible mis-assembly of sequence reads (Fig. S1). The supplement contains detailed lists of the mutated genes or mutations in regions outside the open reading frames. These were sorted by the mutant strain or integrated into the transcriptional landscape of the respective four replicons of *C. metallidurans* (File S1 to S3).

All 34 mutants showed the intended genotypes. This had already been checked by PCR or Southern blot DNA/DNA hybridization (42, 43). The genomic sequences confirmed these results. In mutations generated by interruption via integration of a plasmid containing a part of the target gene by homologous, single-cross recombination and subsequent selection using plasmid-encoded resistance markers (abbreviated as “*gene::I”*), the sequence reads were annotated to the separated segments of the interrupted genes. In these cases, polymorphisms at low-coverage regions clearly marked the integration site. All the published mutant strains had the desired genotype, and this genotype was stable over the years, which is an important control concerning the work of the last decades.

As in the published genomic sequences of 12 strains (13), four large deletions on the chromosome (A, B, E, and J) and one (C) on the chromid were present in most strains (Fig. 10; all large deletions are characterized in detail in the Supplement). While only two more deletions appeared in the chromosome (G and H) in addition to deletions A, B, E, and J in the newly characterized mutant strains, many deletions were found on the chromid, which was less stable than the chromosome. Every mutation or omission of selection pressure for metal resistance resulted in large deletions. Only the wild-type strain remained stable when revived at least once a year from frozen stock culture and kept under selection pressure.

In addition to the large deletions, 81 almost certain mutations plus 72 possible ones were identified in the 35 bacterial strains (Fig. 10; Table 1). Among these mutations, 14 (+22 possible mutations) were identified between the open reading frames of genes, and 67 (+50 possible mutations) were identified within genes. Among these, 12 (+5 possible mutations) appeared in sister mutants, leaving 55 (+45 possible mutations) unique mutations and 36 (+35 possible mutations) in expressed genes. About one-third of the unique mutations in expressed genes were substitutions, most of which were transitions, followed by small deletions, silent mutations, frame-shift mutations, insertions, and truncations.

**TABLE 1:** Number of single or multiple nucleotide polymorphisms^*a*^

Change in genomic sequence	Number
All mutations	81 + 72
Inter-gene	14 + 22
Genes	67 + 50
Minus those occurring in sister strains	55 + 45
Not expressed	19 + 10
Expressed	36 + 35
Deletions	8 + 1
Frame shifts	4 + 6
Insertions	2 + 1
Silent	7 + 4
Substitutions	13 + 21
SNP, transitions	10 + 4
SNP, transversions	3 + 8
In more than one base pair	0 + 9
Truncations	2 + 2

^*a*^ All changes identified by the SNP search in Geneious were cured by assembly, annotation, and other sequencing artifacts, which occurred in the flanking regions of intended or naturally created deletions, reads mis-annotated due to multiple paralogs such as *tnpB*, or regions with low coverage. In the first genomic sequences from number 1 to number 21, only changes that were present in the MiSeq and NextSeq double reads with variant frequencies > 50%. Listed are the number of almost certain mutations (variant frequencies ≥ 90%) plus possible mutations (> 50% and < 90%). Finally, changes in sister strains were subtracted, subsequently those in genes that were not expressed (NPKM < 10), and the remaining mutations in expressed genes were sorted according to the effect of the mutation on the predicted protein.

All types of mutations were thus found. The respective descendants (Fig. 1) also carried the mutations of their parent strain in cases of a high variant frequency. For the possible mutations, the probability of an inheritance increased with the frequency of the respective polymorphism. In the subsequent analysis of the mutations, only those that were new in a strain compared to its parent (Fig. 1) were considered, listed, and described.

An extended version of the Results in the Supplement contains detailed information about all the sequenced bacterial strains and their mutations for the Results sections from “Genome stability” to “Folate biosynthesis.”

### Genome stability

*C. metallidurans* CH34 wild type retained all genomic islands and plasmids intact, with no further deletions (Fig. 10). All SNPs on the chromosome yielded frame shifts and resulted in open reading frames encoding proteins with a high similarity to orthologs in other bacteria (Fig. S2 and S3). Since the probability that a random mutation creates a fully functional gene from an inactivated one is low, the four SNPs on the chromosome of *C. metallidurans* wild type were probably caused by errors in the more than 20-year-old published (10) genomic sequence, highlighting the increase in sequence fidelity during the last two decades. Importantly, these findings demonstrate that the genome of *C. metallidurans* CH34 wild type was stably inherited.

The mutant CH34_1 of strain CH34 originated spontaneously and was different from the wild-type strain in several respects, for example, a larger cell diameter and a coccoid cell morphology were observed (13). As published, it contained the two large deletions A and B on the chromosome, deletion C on the chromid, and D on plasmid pMOL28 (13). The type D deletion in plasmid pMOL28 removed the *chr-cnr* metal-resistance determinants and was unique to strain CH34_1 (Fig. 10, Supplement features of the large deletions). The genome of strain CH34_1 with its altered cell morphology contained nine SNPs, five of which were classified as "possible" mutations. None led to insights concerning the phenotype of this strain (File S1).

Strain CH34_1 was kept for more than 10 years with monthly transfer on Tris-buffered mineral salts medium without selection pressure. In none of the monthly transfers were revertants with the cell morphology of the wild type observed. Strain CH34 wild type was also maintained under the same conditions without selection pressure or revival from a stock culture, leading to strain CH34_10 (Fig. 1). This strain never showed cells with the round morphology of CH34_1 during monthly transfers. Strain CH34_10 lost the regions B, C, and a part of region A, but none of the plasmids (Fig. 10), which was also verified by PCR (Fig. S4). Additionally, CH34_10 carried the deletions I and R. The CH34_10 genome contained 15 SNPs, 13 within genes. Two mutations were in the intergenic region between the genes encoding the rod shape-determining proteins MreB and MreC. The predicted secondary structure of this region (Fig. S5) demonstrated that these mutations would lead to an improvement of a large stem-loop structure in strain CH34_10, possibly encoding a small RNA involved in control of the cell shape. Interestingly, these mutations were present in strain CH34_10 with its rod-shaped morphology, but not in strain CH34_1 with its coccoid cells. Although CH34_10 contained both plasmids, this strain was less resistant to cobalt and nickel than the CH34 wild type (Fig. S6), but the zinc-resistance phenotype of both strains was similar. This indicated that the regions A, B, C, or the recessive and inactivated *nim* determinant that was absent in strain CH34_10 may contribute to full cobalt and nickel resistance in *C. metallidurans* CH34.

The plasmid-free mutant AE104 contained all regions except the *caiB*-region C on the chromid when revived regularly from frozen stock, but deletion A of the island CMGI-2 (Fig. 10, indicated by a small “a”; Fig. S7A, left hand) and, as indicated by many regions of low coverage, also deletion B (Fig. S7A, right hand) began to emerge. A decreasing coverage of regions thus indicated “emerging deletions” within the cells or population of a bacterial strain: AE104 carries the emerging deletions A and B, plus full deletion of region C. In contrast, after 10 years on an agar plate without selection pressure, strain AE104_10 lost the regions A, B, C, and E. Deletion E comprises genomic CMGI-4 except transposon Tn*6048*, which is located in the middle of CMGI-4. All derivatives of strain AE104 carried deletions A, B, C, and E as full or emerging deletions, except a cadmium-sensitive AE104 mutant, ∆*zur* mutants, and ∆*zupT ∆cobW2* (Fig. 10), indicating a connection between zinc homeostasis and these four large deletions.

To understand how the selection pressure for the plasmid-encoded metal resistance affected mutations and large deletions, strains AE126 (pMOL28) and AE126_10 were considered. The latter strain was kept for more than 10 years on TMM, but with alternating selection for the cobalt-nickel resistance determinant *cnr* and *chr* for chromate resistance. Although regularly revived from frozen stock, strain AE126 carried a nearly complete deletion of region B as indicated by limited annotated sequence reads (Fig. 10, indicated by a “(B)”, Fig. S7B, right hand), accumulation of regions of low coverage on region A (left hand), and an even lower coverage of region C (Fig. S7D). Compared to this, region B was completely deleted in strain AE126_10 despite the selection pressure (Fig. S7C, right hand). Regions A (S7C, left hand) and E displayed the emergence of deletions (Fig. 10). Deletion of plasmid pMOL30 with the large zinc resistance determinant *czc* destabilized regions A, B, C, and E, which again links these regions to zinc homeostasis. No mutations were found in strains AE126 and AE126_10, but two SNPs were found in strain AE104 and two more in AE104_10; however, these did not lead to any insights regarding their cause.

A mutant strain of AE104 named AE104_5 had been identified by chance that had lost its resistance to cadmium and cobalt when compared to its parent (Fig. S8). The genomes of AE104_5 and its parent (designated in the sequencing experiment “AE104_4”) were both sequenced. In its cadmium-sensitive descendant AE104_5, deletions A and E were complete, while region B, including the genes encoding the Calvin cycle enzymes and the soluble hydrogenase, was still present. Three possible mutations were found (Supplement), but none were located in genes for regulators of magnesium uptake, for example, the *pho* regulon, or for riboswitches upstream of the genes of magnesium-importing P-type ATPases *mgtA* or *mgtB*. Interestingly, only one other mutant, a ∆*zur ∆cobW2* double mutant, carried a deletion of island A but maintained region B (Fig. 10). Only very few regions of low coverage were found in region B in these two strains (Fig. S9). In strain AE104_5, decreased resistance to cadmium and cobalt may be connected with the large deletions rather than to other "possible" mutations.

Deletions in regions A, B, C, and E appeared in wild-type strain CH34 when either a plasmid was lost, selection pressure for the presence of the plasmids, or revival from the frozen stock were omitted. When one or two of these conditions were maintained, loss of the regions was limited, but not prevented. Deletions emerged in regions of low coverage. This means that in the pure cultures, an increasing percentage of a subpopulation or replicons within the cells carried the deletions. Selection pressure that allowed maintenance of region B, despite a type A deletion, may be connected to a cadmium- and cobalt-sensitive phenotype of a spontaneous AE104 mutant. This indicated that the four regions A, B, C, and E may be involved in metal, especially zinc, homeostasis, but only when both plasmids were present in the cells of *C. metallidurans,* and selection pressure was continuously applied. The type E deletion appeared exclusively in plasmid-free strains, and in most of them except the AE104 parent, the ∆*zur* mutants, and as the emerging deletion in strain AE126_10 (Fig. 10). Selection for the presence of the plasmids or deletion of *zur* prevented the occurrence of the large deletion E.

### Sigma factors

The four mutants with deletions in genes for extracytoplasmic function sigma (ECF) factors, which all contained both plasmids, had different patterns of large deletions (13, 14). As published, strains DN554 (∆*rpoO ∆rpoL ∆rpoM::pLO2*) and DN546 (∆*rpoQ ∆rpoR*) had type A, B, and C deletions, whereas strains DN555 and DN553 carried complementary deletions (Fig. 10). This connected the genomic island encoding the nickel-dependent hydrogenases to nickel resistance on plasmid pMOL28 and the *caiB* region C to the interaction of iron homeostasis with that of other transition metal cations (13, 14).

A mutation in strain DN546 was found in the *copA1* gene on plasmid pMOL30, which is involved in high-level copper resistance (11). CopA1 is a periplasmic copper-dependent copper oxidase related to CueO from *E. coli* (44–48). The mutation led to an *in*-frame deletion of an MGG in a region containing 18 of the total 36 Met residues of the protein, which also organizes a part of the protein (Fig. S10). Since methionine-rich loops serve as a means of scavenging copper ions in the vicinity of the substrate binding site (49), this may decrease by one the number of Cu(I) ions bound to the protein. Strain DN546 (∆*rpoQ ∆rpoR*) with a disturbed thiol homeostasis may have counteracted this effect partially by evolving a mutation in the genes encoding CopA1 and two factors involved in translation. The other changes in sigma factor mutants were inconclusive. None of these mutants had polymorphisms in the genes encoding other sigma factors or any promoter region.

### Uptake systems

In addition to the A, B, C, and E deletions, the ∆7 strain (∆*zupT, ∆corA1, ∆corA2, ∆corA3, ∆pitA, ∆hoxN,* and *∆zntB*) (22, 23) had additionally deleted the region F that encodes another carnitine dehydratase in addition to *ciaB*. Removal of the two genes for magnesium-transporting P-type ATPases MgtA and MgtB in strain ∆9 resulted in three more deletions in regions G, H, and I being acquired. Deletions G and H were unique to strain ∆9, leading to the loss of a hypothetical exported protein with an uninterrupted stretch of 50 Leu residues in G and two putative uncharacterized proteins in H. Deletion I had also been observed in CH34_10. Both mutants lost a gene for the DNA-binding protein H-NS, which silences foreign genes. Removal of the genes for uptake systems led to not only a loss of fitness (22, 23) but also decreased stability of the genome.

Deletion of the ∆*zupT* gene encoding the main zinc importer in *C. metallidurans* caused severe pleiotropic effects, for instance, problems to fold the beta-prime subunit RpoC of the RNA polymerase, although the cells were still able to accumulate a sufficient amount of zinc (18, 35). Two ∆*zupT* deletion strains that were kept for different periods of time on TMM differed with respect to the types of deletion they acquired. The presence of a copy of the *czcCBAD’* region cloned on the vector pVDZ’2 (50), leading to plasmid pDNA130 (51), neither changed the pattern of large deletions nor yielded unique mutations (Fig. 10).

All ∆*zupT* strains showed the same four mutations: three were inconclusive, and one was found upstream in the promoter region of the heat shock sigma factor RpoH. The mutated region upstream of *rpoH* in strain DN515 (∆*zupT*) and the same region in the parent strain AE104 were cloned upstream of a promoterless *lacZ* gene on vector pVDZ’2 (50) and transferred. into strains AE104 and DN515, which resulted in expression of the reporter gene in both strains (Fig. S11). The mutation upstream of *rpoH* had no effect under the tested conditions.

**Fig 2:**
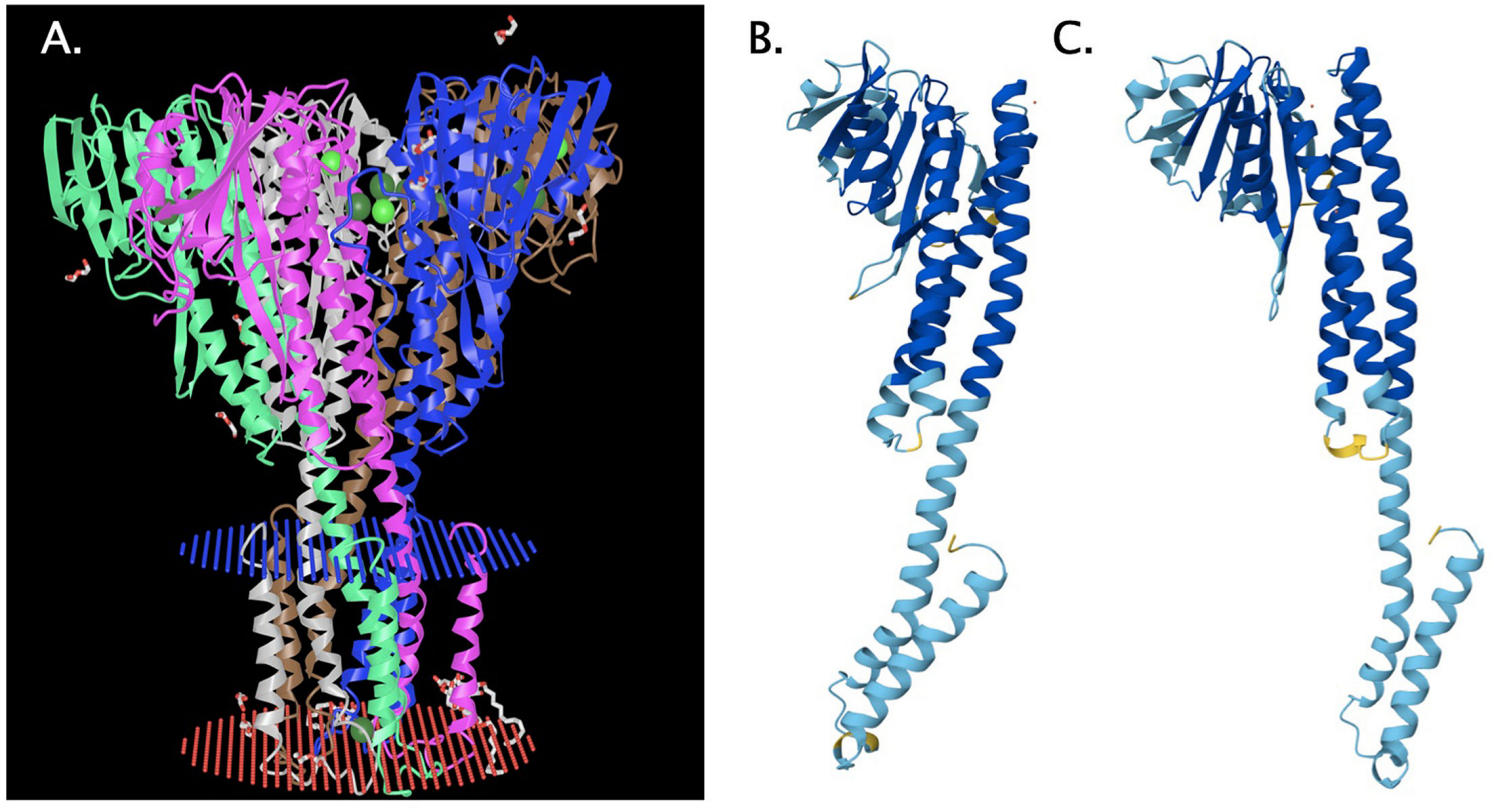
Structure prediction of CorA1 and its mutated derivative from *feoB*. CorA is a pentameric protein (**A**) as shown by the crystal structure of the protein from *T. maritima* 4I0U (52). Each protomer contains two transmembrane alpha helices at the amino-terminal end, with the second helix extending into the cytoplasm. Together with two subsequent helices, three and four, and a beta-region, the cytoplasmic part is involved in a gating mechanism and in flux control. CorA1 (Rmet_3052) monomers from the parent strain AE104 (**B**) and the ∆*feoB* mutant (**C**) were modeled together with three Mg(II) ions (dots) using AlphaFold3 (53). The mutated region lies between helices three and four. Deletion of one A from the AAAAD sequence changed the flexibility of the connection between both helices (yellow, less properly predicted part).

One of the two chromosomal mutations in an AE104 derivative with a deletion in *feoB* of a central iron uptake system was a deletion of one repeat out of four in a (GCC)_4_ motif in the gene for the magnesium uptake system *corA1,* leading to deletion of an alanine residue from an AAAAD sequence. Comparison of the modeled CorA1 protomers from the mutant strain and the parent with the crystal structure of CorA from *Thermotoga maritima* (52, 53) (Fig. 2) indicates that the mutation may change the gating mechanism (54), substrate selectivity, or flux control of this protein. Removal of the central Fe(II) import system of *C. metallidurans* led to a mutation that might be a suppression, which adapts the homeostasis of Mg(II), Co(II), and other divalent metal cations to a barred import route for Fe(II).

Deletion of more genes encoding proteins possibly involved in zinc uptake systems in strain ∆*zupT* (Fig. 1) led to the ∆7 strains (∆*zupT, ∆corA1, ∆corA2, ∆corA3, ∆pitA, ∆hoxN,* and *∆zntB*) (22, 23), which survived at low zinc concentrations, albeit with a decreased fitness (18). It contained, in addition to the type F deletion, three further mutations and four possible mutations. A mutation occurred downstream of the gene encoding the nickel chaperone and GTPase, UreG, a substitution in a membrane protein that might be involved in drug resistance, with signatures of a rhodanese-like sulfur transferase. Together with three possible mutations in Rmet_1656 (*yhjG*), encoding a glutathione-S-transferase-like protein, this suggested that the ∆7 mutant might have acquired suppressor mutations that alter regulatory processes connected to sulfur metabolism. The three possible mutations in Rmet_1656 included a variety of polymorphisms between codons 215 and 244 of this gene, which were mostly below the 50% level. This clustering of polymorphic mutations outside of large deletions or regions of low coverage was designated as “hypervariable regions” in *C. metallidurans*.

**Fig 3:**
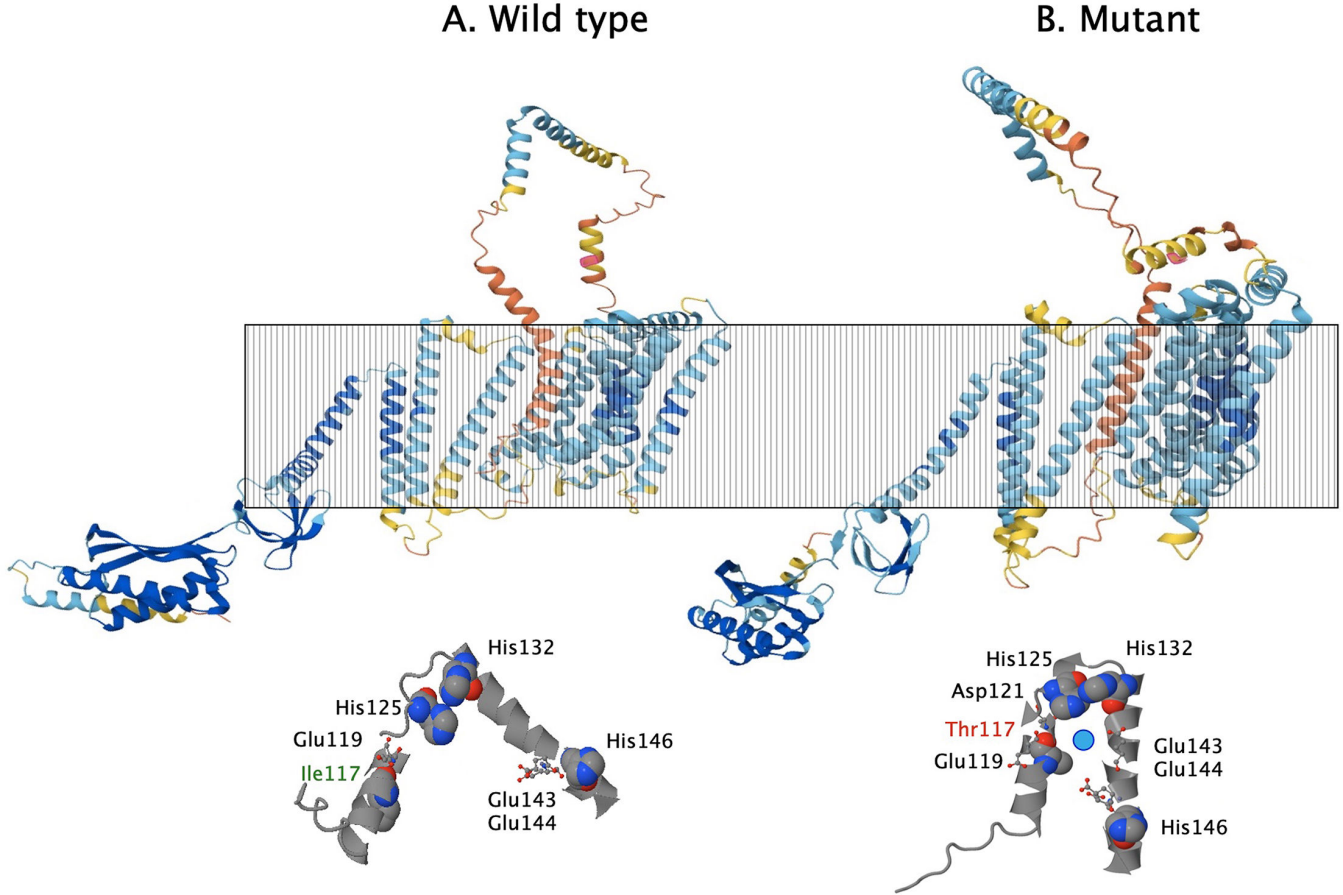
MscS wild type (**A**) and mutant (**B**). Modeled with AlphaFold3 (53) and projected into the cytoplasmic membrane (striped box). The program simulates a change in the predicted periplasmic part of this protein mediated by the Ile - > Thr substitution at codon number 117. The predicted reorganization of the loop is shown below with histidine residues, with space-filled atoms and negatively charged amino acids as ball and sticks. Ile117 in the wild type and Thr117 in the mutant are indicated with green and red letters, respectively. The light blue ball in the mutant loop simulates a Zn(II) ion, which would be in close vicinity to three His and two negatively charged amino acids, which might form a metal binding site.

Strain ∆9 resulted from a deletion of the gene for the Mg^2+^/Ca^2+^-transporting P-type ATPase MgtB and an interruption of its paralog *mgtA*. No marker-free deletion of *mgtA* could be obtained in the ∆7 ∆*mgtB* or the ∆7 mutant (22, 23). Strain ∆9 possessed three additional large deletions and also three additional SNPs. The deletions that were found in the ∆9 strain removed the gene encoding an H-NS-like protein that may silence foreign DNA and a protein involved in RNA interference. In addition to a silent transition on the chromid, a transition in Rmet_3908 led to an I->T amino acid exchange, and an insertion into a tandem repeat region resulted in the insertion of additional G and H amino acids into Rmet_4198. *Rmet_3908* is expressed on a low level and encodes an MscS-type mechanosensitive ion channel, *Rmet_4198* a transcriptional regulator of the PadR-family. The I->T change at position 117 in MscS is located in the first predicted extracellular part of the protein between the transmembrane alpha helices 1 and 2 of this 771 aa protein, which has 12 transmembrane spans. As modeled by AlphaFold3, this change appears to mediate convergence of two periplasmic alpha-helices, which may result in the formation of a zinc-binding site including three His and an Asp or Glu residue (Fig. 3). In ∆9, mutations in regulatory proteins might alter the control of *mscS* expression, and this mechanosensitive ion channel might serve as a zinc importer in the ∆9 strain.

### Efflux systems

Deletion of the four efflux systems known to be involved in Zn(II) export from the cytoplasm to the periplasm (28, 37), namely the two P-type ATPases ZntA and CadA, as well as the two CDF proteins DmeF and FieF (55, 56), resulted in the ∆e4 strain. This strain and its descendants carried the A, B, C, and E deletions plus two additional ones, J and K (13), which may affect outer membrane proteins, via genes for TonB-dependent, methionine biosynthesis, a histidine protein kinase, and a periplasmic substrate-binding protein, all potentially influencing import of metal cations into the cell. Additionally, strain ∆e4 carried seven further mutations, but none had obvious links to metal-ion homeostasis, except a possible change in the DNA-binding N-terminus of a TetR-type regulator (Fig. S12).

Deletion of the gene for the zinc importer ZupT in the ∆e4 strain resulted in two mutations in intergenic regions and six possible mutations, of which five of them are in highly expressed *Rmet_3071* encoding a histone H1-like DNA packaging protein. Reminiscent of *Rmet_1656*/*yhjG* in the ∆7 uptake mutant, the five possible mutations were part of a hypervariable region and were adjacent to a variety of polymorphisms that were below the 50% threshold frequency of occurrence. These mutations should inactivate *Rmet_3071*.

**Fig 4:**
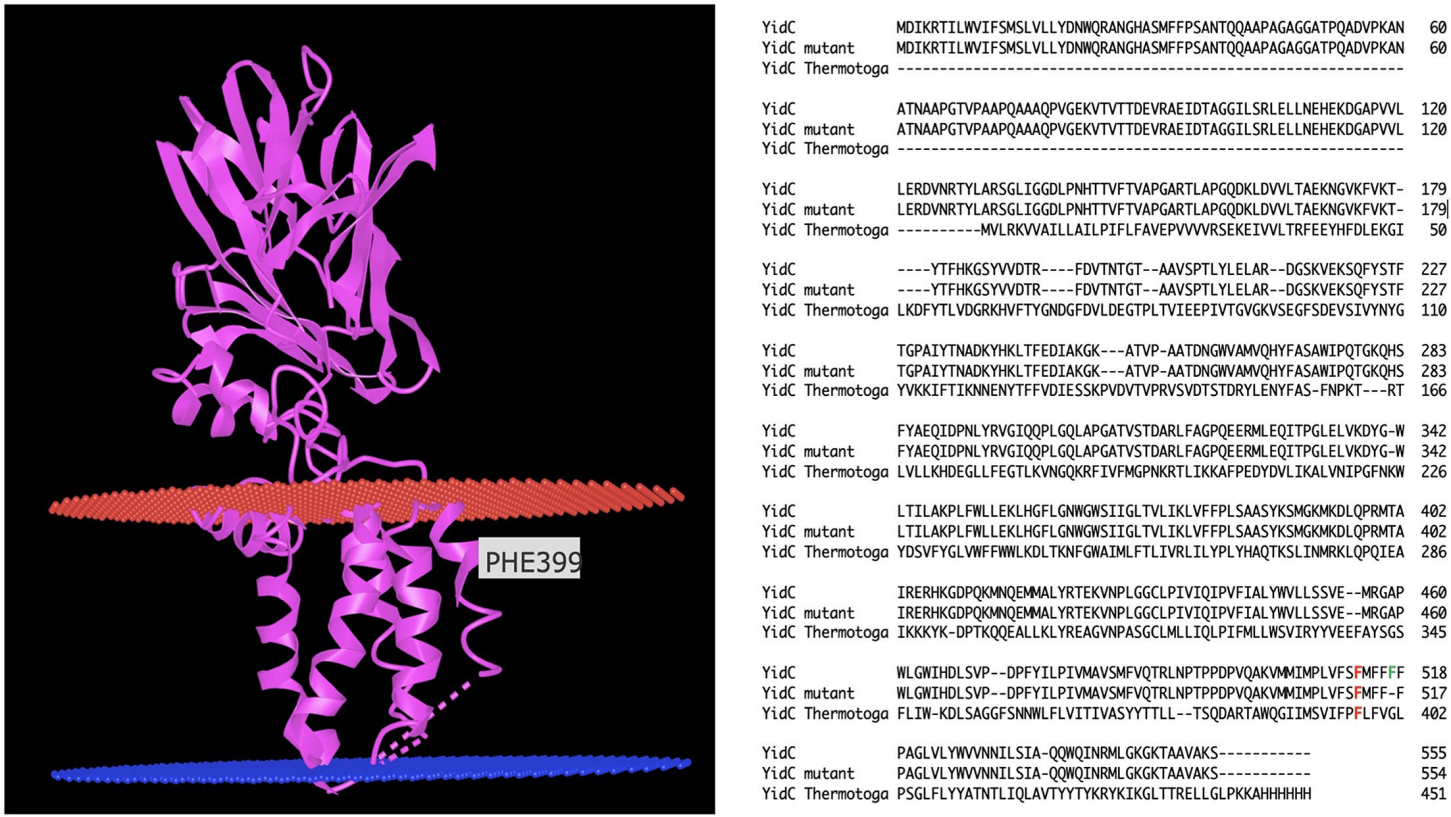
Mutation in YidC in strain ∆e5. Left side, the YidC crystal structure from *T. maritima* 5Y83 is shown (19); right side, a multiple alignment of YidC from *C. metallidurans*, the ∆e5 mutant, and *T. maritima*. Phe399 highlighted in the structure is labeled in red in the alignment, and the missing Phe in YidC from *C. metallidurans* is in green. The beta-barrel structure of YidC is located in the periplasm.

CdfX functions as an additional zinc-efflux system that relieves the main zinc-cadmium exporting P-type ATPase ZntA under certain stress conditions (26). Deletion of *cdfX* in ∆e4 results in the quintuple efflux mutant ∆e5, and this mutant has picked up four mutations, as well as a possible mutation, which are all within genes. One mutation led to the deletion of a Phe residue from a FFFF sequence at position 518 of YidC (Fig. 4). Removal of one Phe from the FFFF sequence in the last transmembrane alpha helix of YidC would shorten it and may influence YidC-mediated insertion of membrane proteins. No additional polymorphism with a low frequency appeared at the region of the deletion of the Phe codon in YidC of the ∆e5 strain, so that in the case of *yidC,* not a hypervariable region but a single deletion of one codon might, act as a suppressor that compensated for the loss of another zinc efflux system.

**Fig 5:**
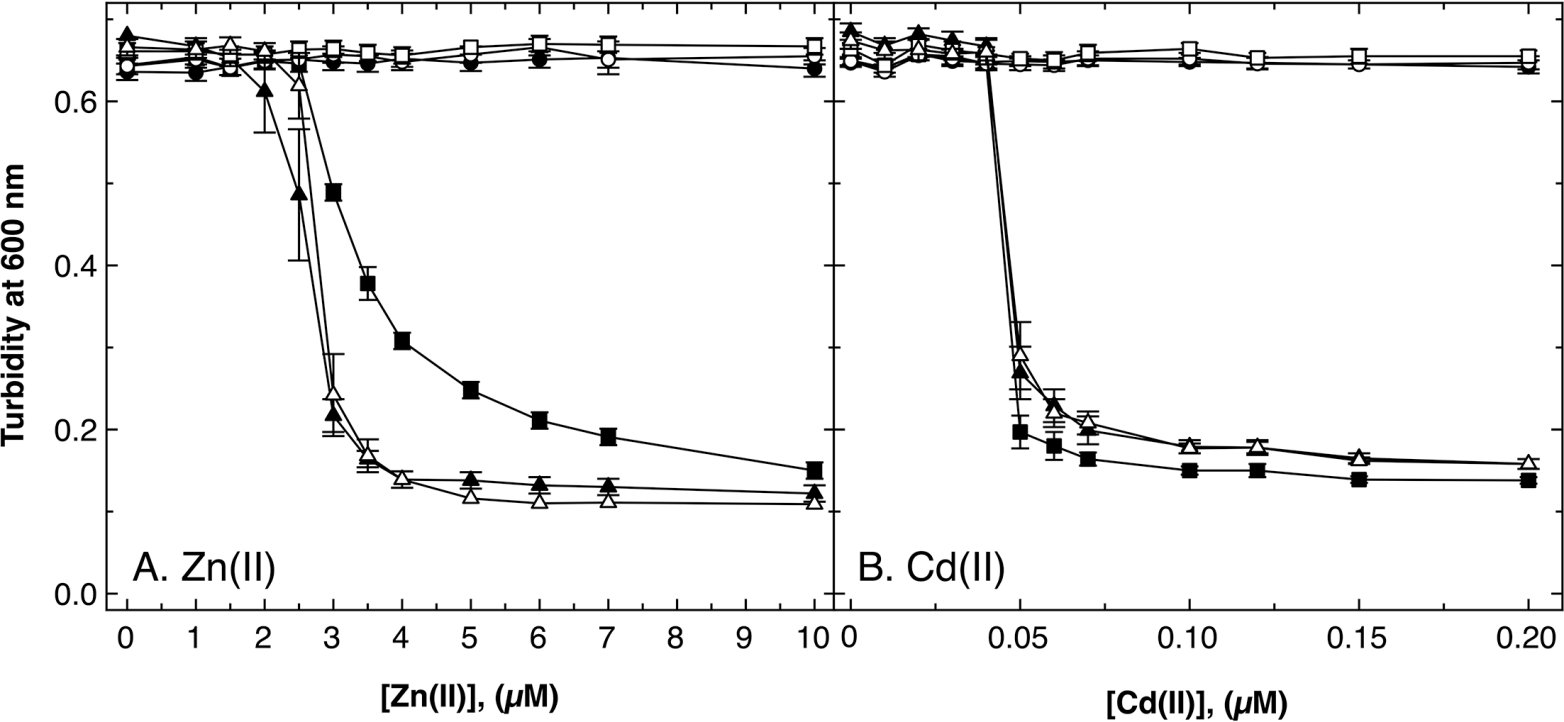
Metal resistance of the ∆e6 mutant of *C. metallidurans* AE104. Dose-response curves showed the measurement of zinc (**A**) and cadmium resistance (**B**) of the strains AE104 parent (closed circles, ●), its ∆*cdfX* (open circles, ○), ∆*cdfX ∆atmA* (open squares, □), ∆e4 (∆*zntA, ∆cadA, ∆dmeF,* and *∆*fieF, closed squares, ■), ∆e5 (∆e4 ∆*cdfX*, closed triangles, ▲), and ∆e6 (∆e5 ∆*atmA*, open triangles, △) mutant in Tris-buffered mineral salts medium TMM. Deviations are shown, *n* ≥ 3.

The ∆e5 strain was still able to efflux zinc, indicating the presence of yet another transport protein with metal efflux activity in *C. metallidurans* (26). A candidate could be the ABC transporter AtmA that is involved in nickel and cobalt resistance (57). The ∆*atmA* gene was deleted in the ∆e5 strain, leading to ∆e6, as well as in the AE104 ∆*cdfX* strain. Zinc and cadmium resistance of AE104 ∆*cdfX* was not affected by the additional ∆*atmA* deletion (Fig. 5). While zinc resistance of ∆e6 seemed to be lower compared to that of ∆e5, albeit not significantly, there was no difference in cadmium resistance.

**Fig 6:**
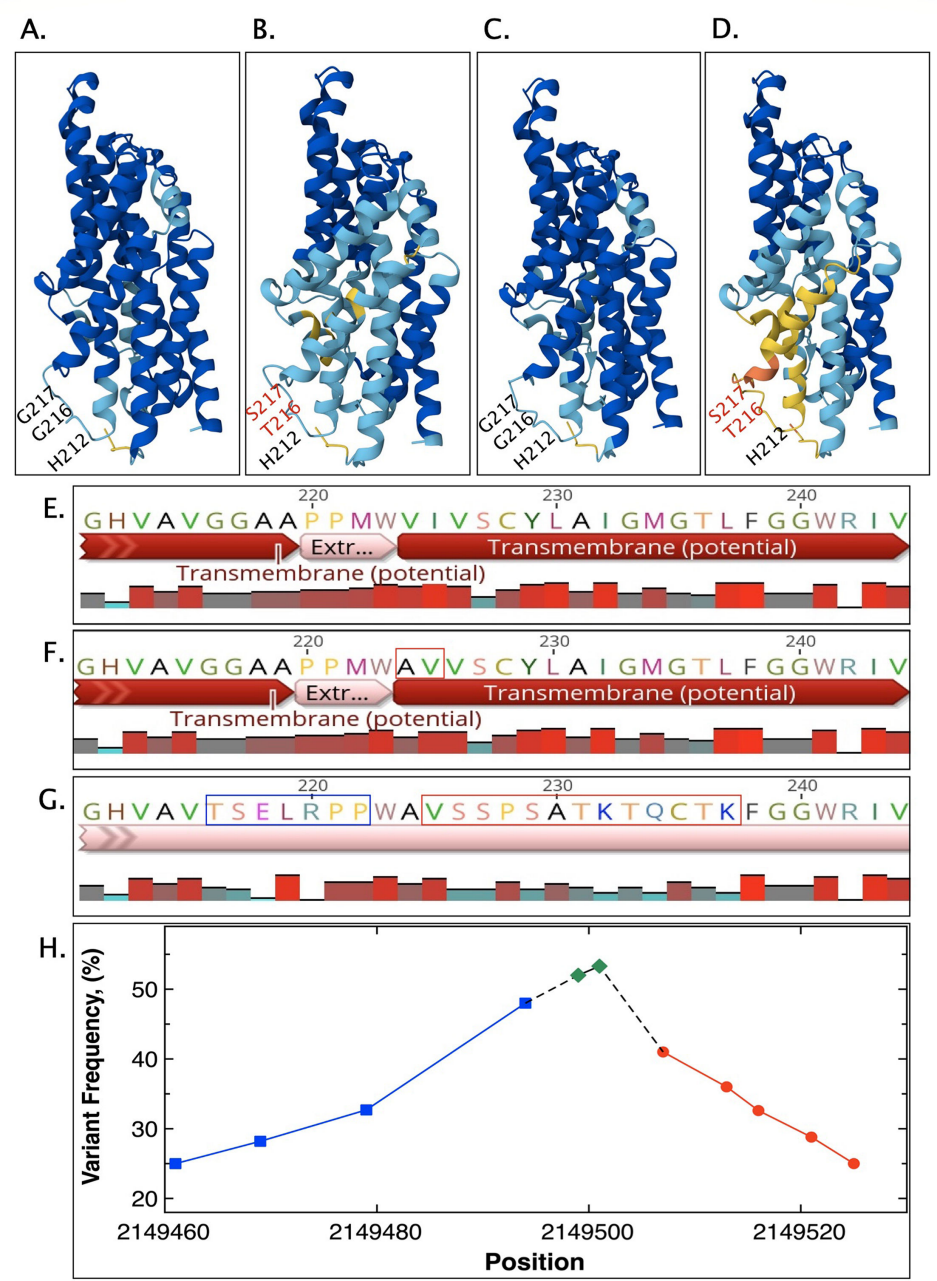
Mutations in the gene for the metal cation phosphate importer PitA in mutant ∆e6. AlphaFold3 (53) models (**A–D**) and transmembrane predictions (**E–G** generated using Geneious) for *C. metallidurans* PitA wild type (**A, E**), its V224A/I225V mutant (**B, F**, mutation in red box), with mutations upstream of this mutation (**C, G** blue box), and a PitA mutant with all mutations (**D, G**, mutated regions boxes) are shown. Panel **H** shows the frequency of the polymorphisms plotted to the position in the DNA. The colors show linked mutations with the two at the frequency peak occurring in either group.

Strain ∆e6 had acquired three mutations, two of which are possible mutations on the chromosome that were again at the center of a hypervariable region. The affected gene was *pitA* encoding the PitA import system for metal phosphate complexes (Fig. 6). Both SNPs were transitions leading to an Ile - > Val and a Val - > Ala substitution. The VI->AV exchanges with a variant frequency >50% were linked either with low-frequency polymorphisms upstream (Fig. 6, GH, blue) or downstream (red), which may affect the formation, stability, or membrane insertion of the transmembrane helices 6–7 (Fig. 6). Loss of all known efflux systems for divalent transition metal cations in the *C. metallidurans* ∆e6 strain results in hypervariable regions in *pitA* in the respective mutant population. Hypervariable regions thus appeared in strains with multiple deletions or, as in the case of ∆*zupT*, when a pleiotropic phenotype indicated a broad impact of the constructed deletion on the bacterial metabolism.

### Zur regulon components

The *zupT* gene, three genes for zinc-binding CobW-GTPases of the COG0523 protein family, and some genes for zinc-salvage proteins are under control of the Zur regulator (31–34, 58–62). CobW1 and the zinc-salvage proteins are only produced under severe zinc-starvation conditions. CobW2 is a zinc-storage protein that occurs in two different conformations. CobW3 no longer has GTPase activity but seems to control the activity of transport systems (18, 31–33).

Deletion of the *cobW* genes was accompanied by deletions in the chromid that were not observed in other mutant strains (Fig. 10). On the other hand, some deletions occurring in these other mutant strains were absent in the ∆*cobW* mutants. All ∆*cobW* mutants with an intact *zur* gene, except ∆*zupT ∆cobW2,* possessed the type A, B, and C deletions plus the type E deletion in the two AE104 derivatives, resembling the AE104_10 strain. Region A was present in the two ∆*zur* mutants, and region B was present in the ∆*zupT ∆cobW2* (Fig. 10), suggesting that different changes in zinc homeostasis stabilized different genomic islands: overexpression of the Zur regulon components the CMGI-2 region A, while it hampered cytoplasmic zinc availability in the CMGI-3 region B.

New types of deletions were type L in the CH34 derivative, types M and N in AE104 ∆*cobW,* and type O in ∆*zur ∆cobW2::*pLO2, all in the chromid. The L, M, and N deletions affected genes upstream of the ancient *czc_2_* determinant. These deletions were part of three clusters of chromid deletions containing overlapping losses. Clusters V, R, N, and Q (red in Fig. 10) could be assigned to *folE* and *cobW* mutants plus strain CH34_10; clusters P, K, S, and O (blue in Fig. 10) are assigned to mutants with multiple deletions in metal efflux systems and Zur regulon components, and clusters L, T, U, and M (green in Fig. 10) are assigned to mutants with mutations in member genes of the Zur regulon.

One SNP in the AE104 ∆*cobW3 ∆cobW1*::pLO2 strain DN837 was a substitution in Rmet_1748, leading to a F->L exchange in the AgrC outer membrane protein of the silver-inducible AgrCBA transenvelope efflux system (63). This SNP was also present in the sister strain AE104 ∆*cobW3 ∆cobW2*::pLO2 DN838. An additional SNP in this strain was an insertion of 3 bp (Gln residue) into Rmet_1033/*slyD* (Fig. S13). The SNPs in the ∆*zur* strain or its ∆*cobW* derivatives or the ∆*zupT* ∆*cobW* mutations could not be associated with a phenotype that would compensate in any way for increased zinc uptake capacity by an overproduction of the zinc importer ZupT or increased zinc handling capacity by CobW1, CobW2, or CobW3. Details for these mutants are provided in the Supplemental materials.

### Folate biosynthesis

In *C. metallidurans* and other bacteria, folate biosynthesis is initiated by a FolE_I-type GTP cyclohydrolase (62, 64). Since tetrahydrofolate is essential for the biosynthesis of GTP, deletion of *folE_I* genes might lead to conditional lethal conditions. *C. metallidurans* possesses three FolE-type enzymes, the strictly Zn-dependent FolE_IA and the two Fe/Mn/Co-dependent FolE_IB1 and FolE_IB2 (30). A triple mutant could not be constructed (30), suggesting that *C. metallidurans* most likely does not possess independent pathways for folate biosynthesis (65). Interestingly, *folE_IB2* is part of the *cobW1* operon, which is controlled by Zur and only expressed under severe zinc starvation conditions (32, 33), such that conditional lethal conditions might indeed exist in a ∆*folE_IA ∆folE_IB1* double mutant when cultivated in the presence of sufficient zinc supply. Because the respective second mutation could only be created as an interruption, this indicated that conditional lethal conditions might have indeed occurred during the strain construction and might have been compensated for by a suppression.

Genomic characterization of the *folE* double mutants was done twice. First, the *∆folE_IB1 ∆folE_IA::I* strain (genome sequence number 33) and its parents were characterized. In a second round using improved protocols for DNA preparation and sequencing, the ∆*folE_IA ∆folE_IB1::I* mutant (number 46) was analyzed (Fig. 10). While in the first characterization, the ∆E1B1 single mutant contained only type A, B, C, and E deletions, and the same strain exhibited an emergent type V deletion in the second experiment performed later. Deletion V involved the loss of the genes for the periplasmic PstS phosphate-binding protein, the *copA_2_B_2_C_2_D_2_* and *copE_2_S_2_* operons, and part of the *nim* operon encoding an incomplete transenvelope efflux system. The second ∆*folE_IA* parent possessed a type Q deletion that included the loss of *furB* encoding the second regulator of iron uptake in *C. metallidurans*. Additionally, the ∆*folE_IA* single mutant carried a type P deletion, which was similar to the type O and S deletions in strains with other mutations in Zur-regulated genes. Loss of other genes involved in metal and phosphate homeostasis in these mutants might represent suppressions that allow an adaptation to handling of zinc and possibly for zinc allocation to the important zinc-dependent FolE_IA enzyme.

One mutation identified in both genomes of both *folE* single mutants was a deletion of 12 bp at a distance of 51 bp upstream of the *murI* gene (*Rmet_2274*) encoding a glutamate racemase important for the synthesis of D-glutamate for the peptide cross-link in peptidoglycan (66). Two additional possible mutations in the ∆E1B1 mutant occurred in the region upstream of the transcriptional start site of Rmet_1033. Close examination (Fig. S13 top) revealed a hypervariable region directly upstream and at the 5′ start of this gene in all *folE* mutants and also in the two ∆*cobW3 ∆cobW1* and ∆*cobW3 ∆cobW2* double mutants, which additionally possessed a SNP, an insertion of an additional Gln residue in a series of already 10 of these residues. *Rmet_1033* is *slyB* and encodes a stress-induced complex with outer membrane proteins during lipopolysaccharide destabilization (67), for instance, as a consequence of a disturbed metal cation homeostasis.

Additionally, all *folE* mutants possessed a hypervariable region in *Rmet_2164,* which was present in no other sequenced strains (Fig. S13 bottom). Rmet_2164 encodes a KAP-P-loop NTPase (68), which may reorganize membrane-associated complexes in an NTP-dependent manner. No polymorphisms in addition to that upstream of *murI* were revealed in the second genomic sequence 44∆1B1 (Fig. 10). Both hypervariable regions in all ∆*folE* mutants, plus the *murI* mutation in the ∆1B1 mutant, seemed to be involved in cell wall-associated stress response.

In both genomic sequences 32 and 45 of the ∆1A mutant, a transition led to a D14- > G14 exchange in the UvrB protein of the nucleotide excision UvrABC system involved in DNA repair in general and also in the Mfd-mediated transcription-coupled DNA repair (69, 70). UvrB is an ATP-dependent exonuclease that binds to the UvrA homodimer and scans DNA for regions of damage. The mutation is located upstream of the N-terminal ATP-binding site in a region not conserved in the UvrD protein of *Bacillus subtilis* (71). AlphaFold3 models a slightly different structure of the ATP-binding wild-type and mutant proteins (Fig. S14), but the N-terminal disordered structure and the first alpha helix that contains the mutated region are not different. Any influence of the mutation on ATP-binding could not be concluded from these structures.

All ∆*folE* single mutants exhibited the mutation in the region upstream of *murI*, the hypervariable regions upstream of *slyB,* and the gene for the KAP-P-loop NTPase. Additionally, deletion in ∆*folE_IA* was coincidental with a mutation in the UvrB gene, which may or may not have a physiological consequence. Together, loss of FolE function seemed to be associated with cell envelope stress (the accompanying Extended Results part ends here).

### Zur

**Fig 7:**
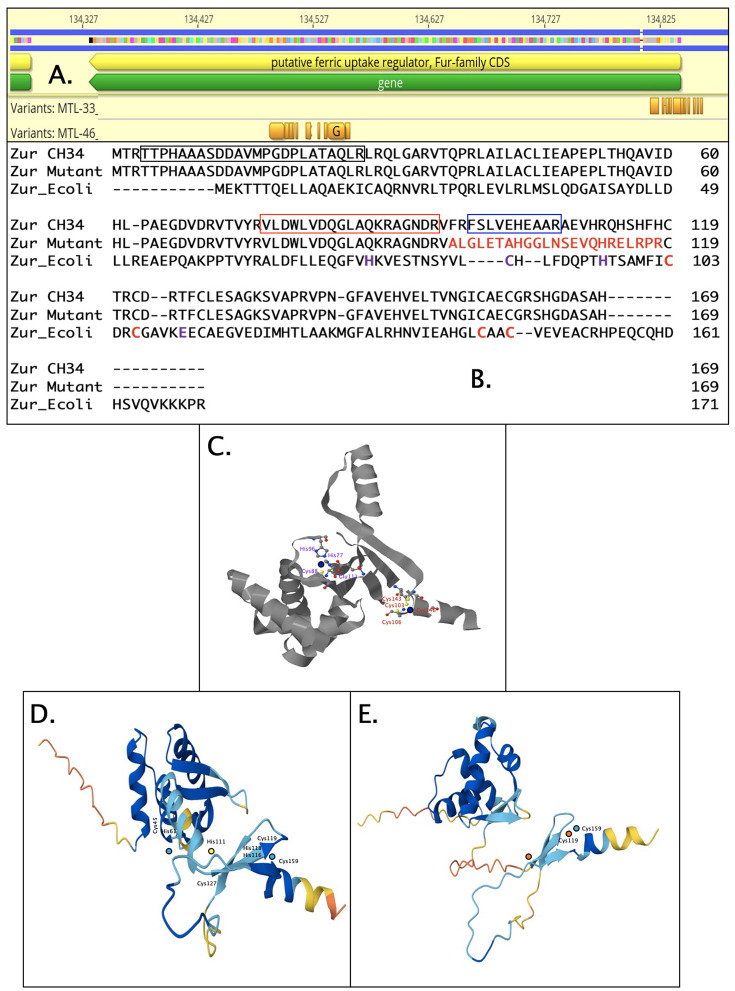
Effect of mutation in *zur*. The screenshot (**A**) shows the hypervariable regions in the ∆1B1∆1A mutant in genome sequence number 33 and in the ∆1A∆1B1 mutant in number 46. Below (**B**) is an alignment of the Zur from CH34, the mutant (changes in red), and Zur from *E. coli* (Zn-binding sites in red and purple for the structural and sensing sites, respectively). The boxes in the sequence of the wild-type protein indicate the peptides used for Zur identification: peptide 1 (black box), 2 (red), and 3 (blue). Panel **C** gives the structure of this protein (4MTD) with the Zn binding amino acids in ball and stick representation. Panels **D** and **E** are the AlphaFold3 prediction of the CH34 enzyme and its mutant.

In one example, the effect of hypervariable regions as a consequence of a conditional lethal condition was investigated in more detail and connected to a phenotype. The last three possible mutations in the 33∆1B1∆1A mutant genome and a mutation in 46∆1A∆1B1 were linked to *zur* (Fig. 7). This gene encodes the zinc uptake regulator, which represses the operon containing ∆*folE_IB2* under conditions of zinc sufficiency. The three possible mutations with variant frequencies > 50% in the ∆1B1∆1A mutant were located upstream of *zur* in the promoter region at positions −1, −4, and −7 relative to the transcription start site, which places them between the −10 promoter region and the discriminator (Fig. 7A). The promoter is RpoD-dependent and possesses a “TTAAAT” −10 region instead of the consensus sequence “TATAAT,” with the second A and last T being the most important bases in *C. metallidurans* (72). The mutant had a G here with a variant frequency of 59%, which should decrease the efficiency of this promoter in the cells carrying this mutation. The mutations in the discriminator would make this part more GC-rich, increasing affinity for RpoD compared to the starvation sigma factor RpoS (73). The three mutations with variant frequencies > 50% were surrounded by additional ones with variant frequencies < 50% (Fig. 7A). Three SNPs upstream with variant frequencies at about 30% never grouped with those with a frequency of > 50%. They turned the first TTs of the −10 sequence into CC, changed the extended −10 “TGG” with the consensus sequence “TGx” into a “CCG,” and deleted two bases from the region between the −10 and the −35-recognition sequence, which decreased the size of this spacer from 16 bps to 14 bps. These mutations, present in about 33% of the sequences, should also decrease the efficiency of this promoter. The remaining four polymorphisms with variant frequencies < 50% were within *zur* because transcription of this gene starts directly at the ATG start codon. These mutations are grouped with those with variant frequencies > 50%. The first one, with a variant frequency between 46% and 55%, resulted in a loss of the start codon, and the other changed the possible amino acid sequence at the N terminus of Zur. Up to 60% of the sequences indicated variants in the ∆1B1∆1A mutant that expressed *zur* with lower efficiency compared to the wild type, with most of them additionally being unable to initiate translation. The second group of sequences with up to 36% variant frequency indicates a strongly decreased expression of *zur*.

In the second double mutant genome 46∆1A1B1, the single mutation in *zur* with a variant frequency > 50% would lead to a *frame shift* due to the loss of two base pairs. Again, this mutation was surrounded by a variety of additional polymorphisms with lower variant frequencies (Fig. 7A). One mutation directly downstream of the AA deletion in the direction of the reading frame inserted two additional base pairs, so that the reading frame was restored. Other mutations resulted in amino acid exchanges. All these mutations would change a sequence of 24 amino acids (Fig. 7B, red letters in the mutant sequence).

Zur proteins contain a structural Zn-binding site composed of 4 Cys residues, as shown for the protein from *E. coli* (Fig. 7B and C red and bold letters, [74]). The dimerization interface and a sensing zinc-binding site are upstream and partly overlapping with the structural Zn-binding site. Zur proteins from other bacteria may contain additional sensing sites (75, 76) with different zinc affinities, allowing a titration of the cellular zinc content (75, 76). The DNA-binding part of Zur may overlap with the sensing site or sites. The changes in the Zur sequence in the ∆1A∆1B1 mutant would be located directly upstream of the structural zinc site, which may lead to a strong change in the Zur conformation (Fig. 7D and E), and interfere with the binding and sensing of zinc. The deletion and insertion were found in the same sequence in two-thirds of the about 360 coverages. The polymorphisms with the low variant frequencies group with the deletion-insertion region, but this overlap was either with the polymorphisms upstream or downstream of the deletion-insertion sites. There was no evidence for wild-type sequences, suggesting that the mutant ∆1A∆1B1 cell population may contain a variety of Zur proteins with different sequences upstream of the structural Zn-binding site (Fig. 7A, red letters in the mutant sequence).

**Fig 8:**
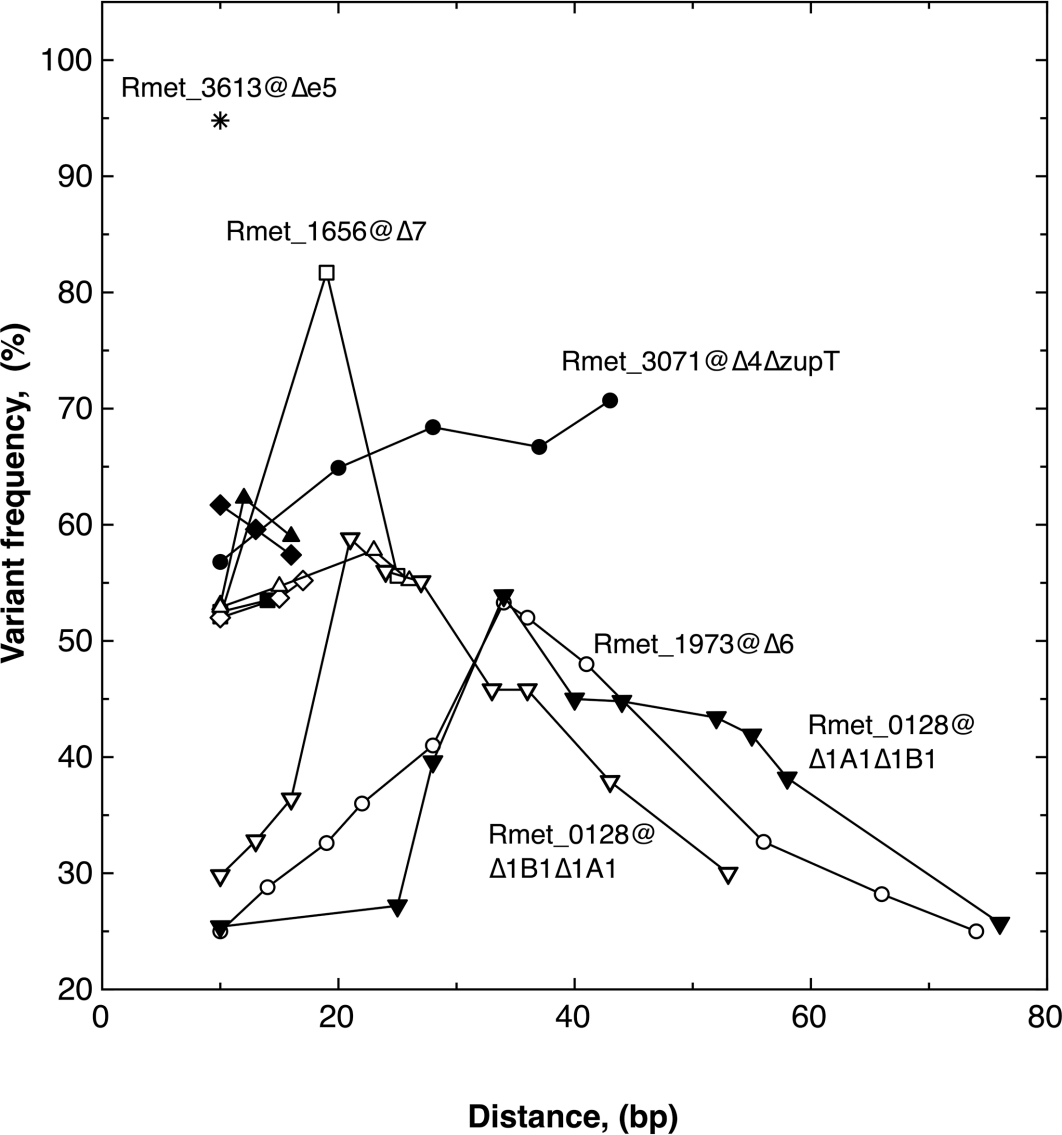
Hypervariable regions. The variant frequencies of polymorphisms were normalized to position 10 for the respective first polymorphism with a frequency larger than 25 and plotted to the resulting normalized position. These were polymorphisms in Rmet_1973/*pitA* in mutant ∆e6 (open circles, ○), Rmet_3071 in ∆e4∆*zupT* (closed circles, ●), Rmet_1656/*ygjG* in ∆7 (open squares, □), Rmet_1577/*betB* (closed squares, ■), Rmet_3361 (closed diamond, ◆), Rmet_5469 (open diamond, ◇), all three in CH34_10, Rmet_4700 in CH34_1 (closed triangle, ▲), Rmet_2164 in ∆1B1 (open triangle, △), as well as Rmet_0128/*zur* in ∆1A∆1B1 (closed inverted triangle, ▼) and ∆1B1∆1A (open inverted triangle, ▽). The star (✳) indicates the comparison of a polymorphism with only one change with a high variant frequency within Rmet_3613/*yidC* in mutant ∆e5.

Creation of mutants in *C. metallidurans* was associated with (i) deletions in CMGIs or other regions in the vicinity of transposase genes; (ii) mutations that are candidates for a suppression as in *yidC* (Rmet_3613); or (iii) hypervariable regions. The hypervariable regions close to or within open reading frames of *zur*, *pitA,* and other genes were normalized to the first polymorphism with a variant frequency above the threshold of 20% at position 10, and the variant frequencies were plotted against this value (Fig. 8). All these hypervariable regions covering a region of up to 70 base pairs displayed a peak with the highest frequency between 50% and 85%, flanked by polymorphisms with lower variant frequencies. However, the mutation at the peak never reached a variant frequency of > 90%, while mutations, as in *yidC* (Rmet_3613), never exhibited flanking polymorphisms. This separated candidates for a suppression by just one mutation from a possible suppression mediated by hypervariable regions. In the case of hypervariable regions, the sequence polymorphisms indicate that the cells or the population of the mutant should contain a variety of proteins with changes in a small part of the protein or the expression pattern of the corresponding gene.

### Effect of a hypervariable region in *zur* in the mutant ∆1A∆1B1

The *folE* double mutant with a marker-less ∆*folE_IA* deletion and an interrupted ∆*folE_IB1* gene (genomic sequence number 46) was characterized in more detail to understand the effect of the Zur polymorphism. A Φ(*folE_IB2-lacZ*) fusion was constructed in the parent strain AE104 and various mutants. While not much reporter activity could be determined in the parent strain (Table 2), deletion of the zinc importer ZupT, which results in problems with zinc allocation, increased expression of the fusion, while removal of Zur increased it even more. In contrast, deletion of *folE_IA* or of *folE_IB1* had no effect. The *folE_IB2* gene was strictly under Zur control, and its expression did not change when folate biosynthesis was compromised.

**TABLE 2:** Activity of a Φ(*folE_IB2-lacZ*) fusion under various conditions^*a*^

Bacterial strain	U/mg dry mass
AE104	0.96 ± 1.68
AE104Δ*zupT*	20.9 ± 14.6
AE104Δ*zur*	468 ± 17.2
AE104Δ*folE_1A*	0.60 ± 0.70
AE104Δ*folE_1B1*	0.89 ± 1.37

^*a*^ The beta-galactosidase activity of the F(*folE_IB2-lacZ) *fusion is shown. Since only interruptions, but no marker-free deletion could be obtained when the *folE* double mutants were constructed, no selection marker was available to construct a F(*folE_IB2-lacZ) *fusion in the double mutant.

**Fig 9:**
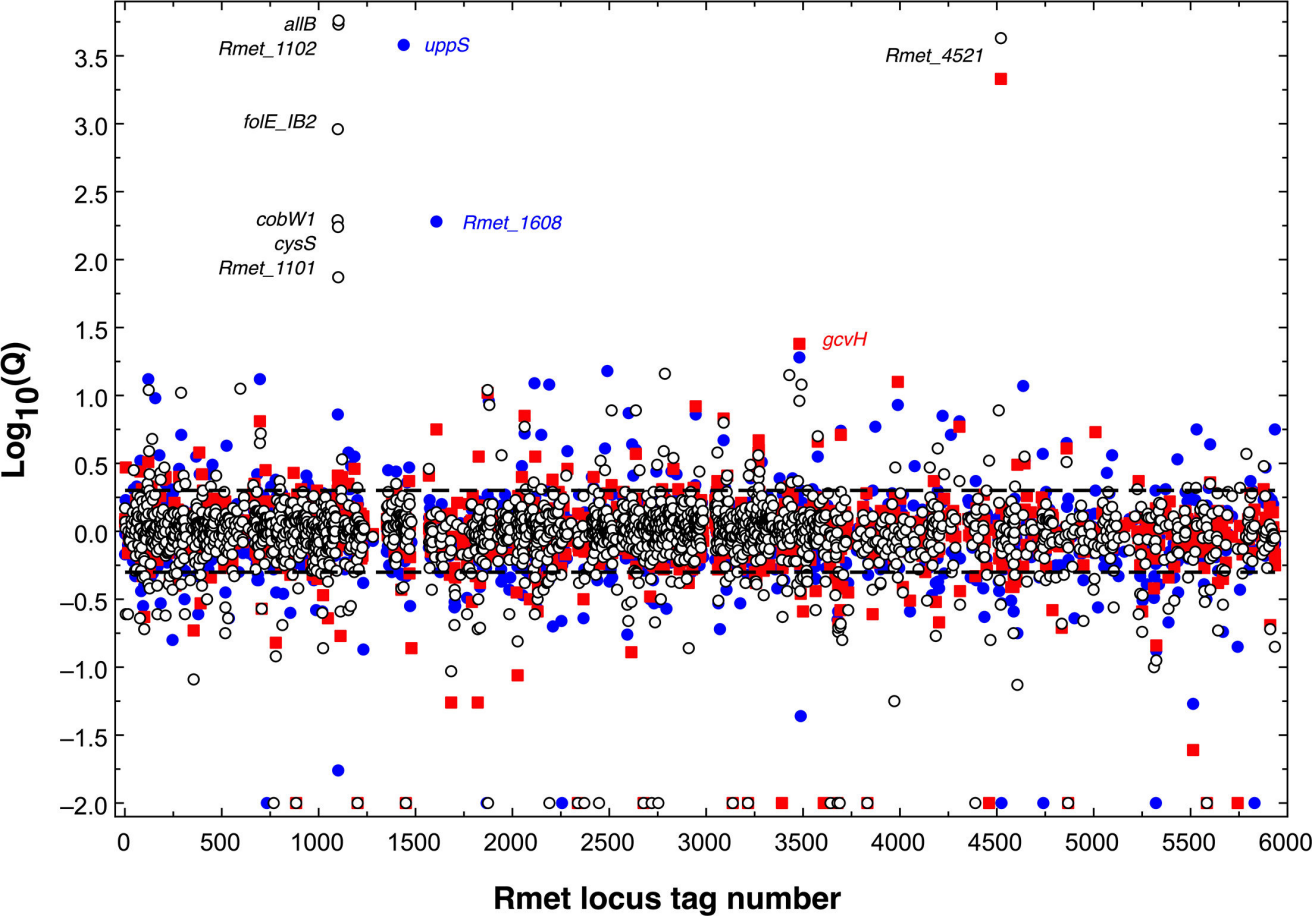
Up- and down-regulated proteins in the proteomes of *folE* mutants. The decadic logarithm of the ratios of the protein number in the ∆*folE_IA* (blue closed circles, ●), the ∆*folE_IB1* (red closed squares, ■), and the ∆*folE_IA ∆folE_IB1::I* (open circles, ○) were plotted against the Rmet locus tag number. Proteins no longer found are indicated at position −2. The dashed lines indicate the borders of a 2-fold up- or down-regulation, respectively. Selected proteins are labeled.

Unfortunately, it was not possible to construct a Φ(*folE_IB2-lacZ*) fusion in either of the *folE* double mutants. Instead, the proteome of the ∆*folE_IA, ∆folE_IB1,* and the ∆*folE_IA ∆folE_IB1::I* mutants was characterized using three independent biological samples of each strain and the parent AE104 (File S3; Table S1; Fig. 9). In the double mutant, but not the single mutants, all proteins encoded by the Zur-controlled *cobW1* operon Op0317f_1 were strongly upregulated, from 75-fold to 5,815-fold (Fig. 9); additionally, CobW3 was 3.9-fold and CobW2 was 2.6-fold upregulated. DksA, encoded by the gene located between *cobW2* and *cobW3,* was not regulated. Zur is encoded by a gene upstream of *cobW2* and is under autogenous control. The gene displayed a lower copy number by 38% of the number determined in the parent, but this value was not significant due to large deviations of the measurement.

Noteworthy was a strong upregulation of the gene encoding undecaprenyl pyrophosphate synthetase, as was the gene encoding GcvH, a component of the glycine-cleavage system and which is required for biosynthesis of tetrahydrofolate, both of which were upregulated in the ∆1A and ∆1B1 single mutants. The upstream region of the *gcvH* gene carried a ZMP/ZTP-dependent *pfl* riboswitch (30), indicating its response to disturbed GTP synthesis in both mutants. In all mutants, ZniR encoding a response regulator was downregulated, involved in the control of the synthesis of the ZniCBA transenvelope system, which is involved in zinc acquisition (15).

The proteomic data impressively indicated the effect of the *zur* polymorphism in the ∆*folE_IA ∆1B1* double mutant, although Zur was still encoded in the cells of this strain, albeit with a lower copy number of 38% compared to that of the parent strain AE104. Zur was identified by three peptides in mass spectrometry (indicated by boxes with different colors in Fig. 7B in the wild-type sequence, Table 3). Peptide 1 (black box) at the N-terminus was clearly identified in the single mutants and the parent. This indicates that Zur indeed started with M1 as annotated.

**TABLE 3:** Peptides used to identify and quantify Zur in the mass spectrometry^*a*^

Sample	Peptide 1	Peptide 2	Peptide 3
Annotated sequence	{R}.TTPHAAASDDAVMPGDPLATAQLR.{L}	{R}.VLDWLVDQGLAQK.{R}	{R}.FSLVEHEAAR.{A}
Positions in master proteins	Q1LS62 (aa 4–27)	Q1LS62 (aa 76–88)	Q1LS62 (aa 98–107)
Theo. MH^+^ (Da)	2,406.1718	1,484.81,075	1,158.5902
Abundances soluble + membrane, control	104 ± 94 + 10.1 ± 16.6	209 ± 51 + 0.0 ± 0.0	70.5 ± 13.8 + 185 ± 154
Control high/peak soluble + membrane	0/2 + 1/2	0/1 + 0/0	0/1 + 2/1
Abundances soluble + membrane, ∆1A	387 ± 54 + 273 ± 33	193 ± 16 + 0.0 ± 0.0	226 ± 193 + 369 ± 62
∆1A high/peak soluble + membrane	1/1 + 3/0	1/1 + 0/0	2/1 + 2/1
Abundances soluble + membrane, ∆1B1	205 ± 210 + 0.0 ± 0.0	172 ± 91 + 0.0 ± 0.0	93.2 ± 108.4 + 17.7 ± 13.3
∆1B1 high/peak soluble + membrane	2/1 + 0/0	0/2 + 0/0	1/2 + 0/3
Abundances soluble + membrane, ∆1A ∆1B1::I	13.6 ± 2.2 + 7.2 ± 0.8	426 ± 115 + 0.0 ± 0.0	37.5 ± 5.3 + 0.0 ± 0.0
∆1A ∆1B1:I high/peak soluble + membrane	0/1 + 0/1	0/1 + 0/0	0/1 + 0/0
Abundances soluble + membrane, ∆1B1 ∆1A::I	0.0 ± 0.0 + 0.0 ± 0.0	0.0 ± 0.0 + 0.0 ± 0.0	0.0 ± 0.0 + 1.2 ± 0.2
∆1B1 ∆1A::I high/peak soluble + membrane	0/0 + 0/0	0/0 + 0/0	0/0 + 0/1

^*a*^ Zur was identified and quantified using three different peptides, resulting from protease cleavage. The first lines give their sequences, position, and molecular mass. The subsequent lines give for each strain the abundance of the respective peptide in the three biological samples in the supernatant plus solubilized pellet of the ultracentrifugation step used to prepare the samples, and below the number of samples leading to a quality of the measurement judged by the analysis software as “High”/”Peak.” Zero means no peptide found in none of the three biological samples.

In the parent strain AE104 and the two ∆*folE* single deletion strains, all three peptides were found and used to quantify the number of Zur proteins per cell. Peptides 1 and 3 occurred at least three times in the determination, while peptide 2 was found only once or twice in the respective six samples (three biological experiments with an ultracentrifugation supernatant and solubilized pellet for each reproduction). In cells of the ∆1A ∆1B1::I mutant (genomic sequence number 46), peptide 1 was found twice and peptide 3 once (Table 3), and both showed lower abundance compared to the parent and the single mutants. Peptide 2 was found only once with high abundance. Taking only peptides 1 and 3 into consideration, the number of Zur molecules per cell should be 13.1% and 14.7%, respectively, of the number in the parent cells. Nevertheless, Zur was present in the cells of this double mutant, and even peptide 3, which should not appear in the fully mutated protein (Fig. 7A, red letters in the mutant and the blue box for peptide 3 in the mutant sequence), was identified. To obtain additional information, the Zur level was also determined in the proteome of the second ∆1B1 ∆1A::I double mutant (genomic sequence number 33), which should not express *zur*. Peptide 3 was found here in one sample only and with a very low abundance (Table 3). Only traces of Zur were present in the cells of this double mutant.

## DISCUSSION

### Stability of the genomes of *C. metallidurans* CH34 wild type and its mutants

The genome of *C. metallidurans* CH34 wild type was stable but only when regularly revived from frozen stock and kept under alternating selection pressure to maintain the presence of its two plasmids. The few differences from the published sequences (10, 13) may be the result of sequencing errors in the older sequence or differences in the sequenced strains. Strain CH34 was isolated in Mol/Belgium in the 1970s (2, 77), subsequently distributed to other laboratories (1), and sequenced in 2001 for the first time (10). The strain that was sequenced later in 2020 had been in Göttingen (Germany), Berlin (Germany), Chicago (IL, USA), and Halle (Saale), Germany (13). In light of more than 40 years of a varied history of the two versions of CH34 wild type, just five SNPs in the six million base pair genome are a surprisingly low number.

On the other hand, the genomic stability of *C. metallidurans* requires strict observance of the correct conditions. Any omission or mutation results in genomic changes that are, however, not random but evolve reproducibly in sister strains. As previously published (13), large deletions remove the genomic island CMGI-2 containing genes for the membrane-bound nickel-containing hydrogenase (Type A deletion), CMGI-3 for the soluble, NAD^+^-reducing hydrogenase and the enzymes for the Calvin cycle (B deletion), the *caiB* region of two genes on the chromid (C deletion), and the island CMGI-4 (E deletion) in most strains (8, 9, 13). Region E contains the genes for the recessive *hmz* determinant, which is composed of three adjacent operons (File S2). Op0848r_4 in the reverse direction contains the genes for a ferric reductase and the two-component regulatory system HmzRS. Op0847f_1 in the forward direction comprises two genes for a cytochrome b*561* and a transporter related to the vacuolar iron transporter VIT1 (78). Op0846r_1 is in the reverse direction again and contains the genes for a membrane fusion protein (MFP) and the RND protein HmzA, which is truncated at its C-terminus due to insertion of Tn*6048* into the 3′ end of *hmzA*. Instead of the gene for an outer membrane factor, which is transcribed in some RND-encoding operons in the vicinity of the MFP and RND genes but in the opposite direction of transcription, the *hmz* determinant encodes a cytochrome and a possible iron transporter. This indicates a possible role of the *hmz* determinant in iron homeostasis.

**Fig 10:**
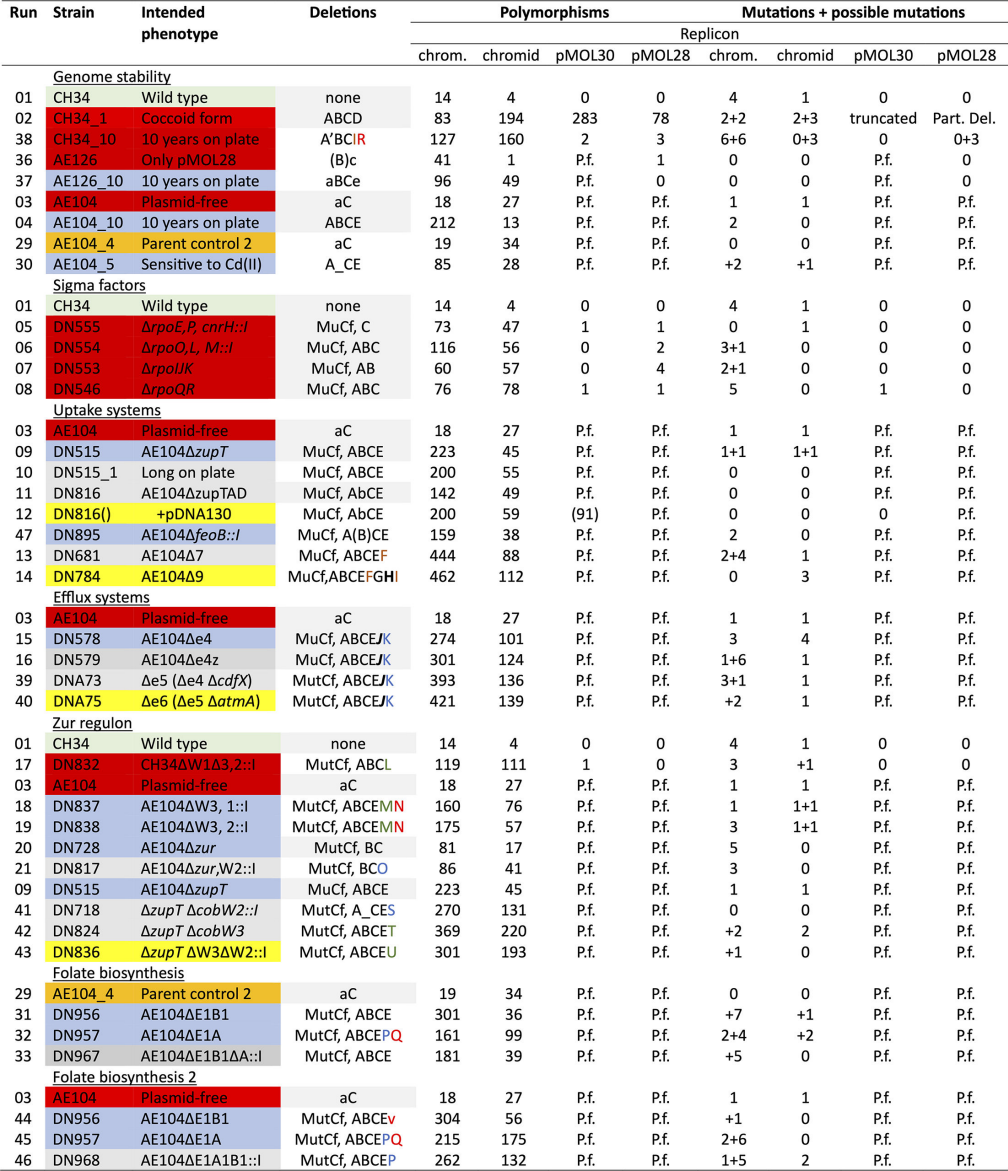
The number of potential reproducible polymorphisms in 21 newly sequenced strains of *C. metallidurans* CH34. The polymorphisms, mutations, and possible mutations in a series of newly sequenced strains of *C. metallidurans* CH34 are listed. “Polymorphisms” were defined as all changes identified by the SNP search in Geneious. They were cured from assembly, annotation, and other sequencing artifacts, which occurred in the flanking regions of intended or naturally created deletions, reads misannotated due to multiple paralogs, such as *tnpB*, or regions with low coverage. In the first genomic sequences from number 1 to number 21, only changes that were present in the MiSeq and NextSeq double reads with variant frequencies > 50% were considered. The number of almost certain mutations (variant frequencies ≥ 90%) plus possible mutations (> 50% and <90%) is listed as outlined in the text. In the rows “strain” and “intended phenotype,” the field colors indicate whether the bacterial strain is wild-type CH34 (green), a primary derivative of this strain (red), a secondary (blue), tertiary (gray), or a quaternary (yellow) derivative, as shown in Fig. 1. A reproduction of the genomic sequence of the plasmid-free strain AE104 is on a field in orange. In the row “deletions,” some have already been published (13) and are on a gray field. “MutCf” means that the created deletions were confirmed. The secondary deletions (not intended, following the constructed deletion) were named with a capital letter when complete, in parentheses when nearly complete as indicated by only a few remaining reads (<50 compared to about 800 elsewhere), or in small letters in case of a starting deletion as demonstrated by a region with a lower coverage compared to that in the vicinity of the region. Deletions on the chromosome: A: ∆CMGI-2 ∆*Rmet_1236-1351* (A’, deletion incomplete and starts at *Rmet_1253*); B: ∆CMGI-3; deletion of the central part from *Rmet_1492 = cbbA1* to *Rmet_1541 = hoxX;* E: ∆CMGI-4 except Tn*6048*; G: ∆*Rmet_*6477; bold faced **H**: ∆*Rmet_2162_2161,* and ***J***: ∆Rmet_6403_0032. Deletion of a gene for a hypothetical protein and an outer membrane protein. Deletions on plasmid pMOL28: D: Deletion from downstream *tnpA* (Rmet_6188, Tn*4378*) to downstream of reverse gene Rmet_6245, including *chr* and *cnr* determinants. Deletions on the chromid: C: ∆*Rmet_5543/5544* (*caiB* and a gene for aldehyde dehydrogenase). Groups of chromid deletions that are overlapping or in the same region: In orange letters: F: ∆*Rmet_5535-5536;* I: ∆*Rmet_5561_6747_5562_5563*. In red letters: N: ∆*Rmet_5689-5754*: plus the following deletions Q, R, V in the same region: Q: ∆*Rmet_5739-5754* (including *Rmet_5746* = *furB*); R: ∆*Rmet_5681-5754*; and V: ∆*Rmet_5657-5717*. In blue letters: K: ∆*Rmet_4603-4618* downstream of *zntA* (Rmet_4594) plus the following deletions O, P, and S in the same region: O: ∆*Rmet_4603-4693*; P: ∆*Rmet_4603-4616;* and S: ∆*Rmet_4603-4651*. In green letters: L: Chromid ∆*Rmet_4208-4462* except Tn*6049* plus the following deletions M, T, and U in the same region: M: ∆*Rmet_4434-4469*; T: ∆*Rmet_4208-4497* except Tn*6049* and Tn*6050*; and U: ∆*Rmet_4370-4469* except Tn*6049* and Tn*6050*. The Supplement contains a detailed description of all deletions and of all mutations, which were also embedded in the transcriptional landscape of the DNA strands of all four replicons of *C. metallidurans*.

Transposon Tn*6048* was not part of the type E deletions, but the regions of CMGI-4 flanking it. The type E deletion occurred in all plasmid-free strains, including AE104_10 and the cadmium-sensitive AE104_5, but not the parent AE104, its ∆*zur* mutants, or AE126, although it was observed as an emerging deletion in AE126_10. All CH34 strains carrying both plasmids still possess CMGI-4, including the sigma factor mutants (Fig. 10). This means that the presence of plasmid pMOL30 with the *czc* determinant or an overexpression of the zinc allocation pathway in ∆*zur* is a prerequisite for the function of Hmz, which could mobilize iron for uptake by cycling, as has been suggested for zinc and the ZniCBA transenvelope system (15). Moreover, the alternative importance of the periplasmic CzcCBA and the components of the Zur regulon indicate that the iron and zinc mobilization and import routes may interfere with each other.

For the remaining deletions A, B, and C that were present in the majority of the strains, the genotype of those strains that still contain these regions shed some light on the function of the genes harbored by the corresponding regions. As discussed previously (13), regions A and B encoding the genes for nickel-containing hydrogenases are both present in the sigma factor mutant DN555, which carries an insertion in the gene for sigma factor CnrH required for expression of the *cnr* cobalt-nickel resistance determinant (79–81). Both regions are lost in strain CH34 when not kept under selection pressure and not regularly revived from frozen stock. Region A with the genes for the membrane-bound hydrogenase was present in strain AE126, which contains only plasmid pMOL28 with *cnr* but shows signs of its emerging deletion in AE126_10, as in the plasmid-free strain AE104. This region was present in ∆*zur* mutants. The presence of the plasmid pMOL30-encoded *czc* region or an overexpression of zinc allocation pathways in ∆*zur* mutants seems to increase the importance of region A in addition to region E. Region B, with the soluble, NAD^+^-reducing hydrogenase and the Calvin cycle enzymes, is present in the sigma factor mutant DN555; it is deleted in most cells in strain AE126 and completely deleted in AE126_10. However, it is maintained in the ∆*zupT ∆cobW2* double mutant DN718 (Fig. 10). This indicates that a fully functional CnrCBA efflux system for cobalt and nickel in the presence of CzcCBA selects against region B, while in the absence of either transenvelope system, a compromised zinc allocation and storage capability allows its retention. Under zinc starvation conditions, cobalt is able to substitute for zinc, and ZupT and CobW2 are involved in the control of this process (31). Moreover, the activity of Czc is quenched by CzcI (27), and CnrCBA mediates cobalt resistance by transenvelope efflux under these conditions. On the other hand, nickel seems not to be able to substitute for zinc (31). This indicates that the synthesis of the hydrogenases may be simply a mechanism to sequester Ni(II) to prevent it from interfering with the already delicate cobalt-zinc mixed homeostasis. The decreased cobalt and nickel resistance of strain CH34_10 that was kept for more than 10 years on agar plates without selection pressure argues for the hydrogenases as sinks and buffers for cellular nickel ions.

The hydrogenases are constitutively produced in *C. metallidurans* (1). Weathering in the natural metal-rich environment of *C. metallidurans* may produce sufficient molecular hydrogen (82, 83) to compensate for the energy used for the synthesis of the hydrogenases, and together with the Calvin cycle enzymes allowing mixotrophic growth. In the laboratory, only strict selection for zinc and nickel resistance prevents loss of the plasmids and provides sufficient zinc to the cells, such that loss of regions A and B does not provide a growth advantage within a limited period of up to 1 year. This situation changes with loss of either plasmid pMOL30, both plasmids, or gene deletions with only very few exceptions (Fig. 10).

Indications of deletions A, B, C, and E occur gradually when genome sequences of different strains were compared. The sequence coverage of the respective regions started with multiple regions of low coverage, followed by low coverage of the complete region. The percentage of coverage decreased subsequently until only a few reads remained that were annotated to the respective regions (Fig. S7). This indicates a gradual decrease of templates for the respective islands in the mutant cells or their population. While regions A and B could be connected to nickel and region E to iron homeostasis, only the sigma factor mutant DN553 (∆*rpoIJK*) still contains region C. These sigma factors are also involved in iron homeostasis (14), but a connection between the loss of a paralog of a carnitine-dehydrogenase and an aldehyde dehydrogenase is not evident. Although this publication did not track the emergence of mutations over time, comparison of freshly revived strains with those kept for 10 years on a plate without selection pressure enabled some understanding of the evolutionary trajectories in *C. metallidurans*.

### Other large deletions were associated with groups of mutants

While the frequently occurring deletions A, B, and E affected the genomic islands CMGI-2, -3, and -4 on the chromosome (8, 9), respectively, changes in the chromid other than type C could be grouped into clusters of overlapping deletions, which, in turn, could be assigned to categories of mutants (Fig. 10, features of large deletions in the Supplement). Deletion types F and I were found in CH34_10 and in the “uptake” mutants ∆7 and ∆9. Among others, the gene encoding another carnitine dehydrogenase and that encoding an H-NS-type DNA-binding protein that silences foreign DNA were deleted (84–87), indicating that in strain ∆7, the decreased fitness may lead to an upregulation of otherwise silenced DNA regions; however, this does not reflect what has been observed (22, 23). The ∆9 mutant carries additional chromosomal deletion types G and H, but the genes deleted in these regions did not lead to new insights with respect to metal ion homeostasis.

Deletions N, Q, R, and V (red in Fig. 10) appeared in ∆*cobW3 ∆cobW1/2* double mutants, strains with deleted *folE_I* genes for the initiation of folate biosynthesis, and strain CH34_10. This region of nearly 100 genes includes, near its center, the *nim* determinant encoding a further transenvelope efflux system. The region also includes an interrupted *nimA* gene encoding the RND component of the efflux system, suggesting a function for the respective outer membrane protein, NimC, and for the membrane fusion protein, NimB, in metal homeostasis. This determinant was also lost in strain CH34_10 so that *nim* may be contributing to the plasmid-encoded cobalt and nickel resistance. In the type R, B, and Q deletions, the *furB* gene for a paralog of the iron-uptake regulator, FurA (34), is absent in the *cobW3 cobW1/2* double mutants, in the ∆*folE_IA* mutant, encoding the zinc-dependent GTP cyclohydrolase, and in CH34_10. The *cobW3 cobW1/2* double mutants contained, in addition to deletion N and the most frequently occurring deletions A, B, C, and E, deletion type M, which is part of another cluster of deletions (L, T, U, and M) (Fig. 10, green). These deletions occurred in all strains with a *cobW3* deletion and affected nearly 300 genes, including the inactivated *czc_2_* determinant, porins, and siderophore receptors. CobW3 resembles a metal-chaperoning GTPase but lacks GTPase activity. It binds zinc and other metal cations in its C-terminal domain, and those with different affinities, and appears to interact with metal-transport systems, including ZupT (31–33). Loss of its function seems to result in an additional deletion of genes encoding many other proteins involved in transition metal homeostasis.

Another cluster comprising the deletions P, K, S, and O (Fig. 10, blue) appeared in mutants with deleted efflux systems, in mutants with defective Zur regulon components, and in ∆*folE_IA* mutants. The deletions affected genes for TonB-dependent outer membrane factors. In the largest deletion, type O, present in the ∆*zur* ∆*cobW2::I* mutant, the genes encoding the sigma factor RpoD2 and RpoQ, and the *gig* determinant were additionally removed. RpoD2 is a paralog of the main housekeeping sigma factor RpoD, while RpoQ and the *gig* gene products are involved in copper homeostasis (88, 89).

All these secondary deletions following the constructed one indicate that not only homeostasis of Zn, Co, and Ni is linked in *C. metallidurans,* but also that of Cu and Fe. CzcCBA and other transenvelope systems in the periplasm, along with ZupT and the other Zur regulon components in the cytoplasm, mediate an intricate zinc homeostasis at the center of this transition metal homeostasis, which subsequently guarantees sufficient synthesis of folate and takes advantage of a variety of additional gene products encoded on the chromid and chromosomal islands. When the central parts of the dominating zinc homeostasis system are compromised, all these additional genes are no longer beneficial or become a burden to the cell, so that deletion mutants outgrow their parents. This part of the ongoing evolution of *C. metallidurans* to adapt to laboratory conditions can be clearly seen by the decrease of sequencing templates for regions A, B, and C, which led to a decrease in coverage of the annotated sequences to these regions (Fig. S7).

### Interference between the secondary and intended mutations

In addition to the large deletions of chromosomal islands, parts of the chromid, or loss of the plasmids, single-nucleotide polymorphisms (SNPs) occurred in most genomes of the mutant strains (Fig. 10). These SNPs may suppress the effect of the introduced mutation, potentially influencing the results of the subsequent characterization of the mutant strains. Two different kinds of polymorphisms were observed. One kind involved single point mutations, deletions, or insertions (InDels) of a few base pairs, which occurred with a high variant frequency (> 90%). The second kind involved hypervariable regions and clusters of adjacent polymorphisms with low variant frequencies (i.e., > 50%). Some SNPs with high variant frequencies were candidates for suppressor mutations. In the ∆*feoB* mutant that has lost the Feo Fe(II) uptake system, a SNP in the *corA1* gene for the main Mg(II) and metal cation uptake system may change the flexibility of the connection between transmembrane alpha helices, which consequently could influence the gating mechanism of the protein and flux control of the import of divalent metal cations (Fig. 2, [52, 90, 91]). Since CorA1 is just one metal import system among nine or more, its individual contribution to metal cation uptake is small (22, 23, 36), so that this change in the gating system should not affect the measurement of metal uptake by the ∆*feoB* mutant cells.

A suppression was even expected in the mutant ∆9, in which all known import systems for transition metal cations were removed. This strain was able to grow, albeit with decreased fitness (22, 23), due to slow import of these metals (18). Surprisingly, when cultivated under conditions with sufficient metal supply, but not under metal starvation conditions, strain ∆9 exhibited zinc import with a higher rate than its ∆7 parent (18). A candidate for the suppression by SNPs in various genes in the ∆9 mutant may generate a zinc-binding site in the mechanosensitive channel MscS and allow expression of the corresponding gene (Fig. 3). This would potentially explain the results of the respective pulse-chase experiments with ∆9 cells (18).

In the mutation ∆e5 with five metal-efflux systems deleted (26), a SNP led to deletion of a Phe from a FFFF sequence at position 518 of YidC (Fig. 4). YidC is required, in addition to SecYEG, for membrane insertion of proteins in bacteria (92), which is needed for translocation of periplasmic domains with negative charges or insertion of transmembrane segments with polar residues, while YidC, together with SecYEG, is needed for processing of regions with positive charges. Together with another alpha-helical domain not clearly visible in the *T. maritima* YidC (Fig. 4), the positively charged C-terminus of YidC is essential for its function (93). The positive charges at the C-terminus are also present in YidC from *C. metallidurans* (Fig. 4). Removal of one Phe from the FFFF sequence in the last transmembrane alpha helix of YidC would shorten it and may influence YidC-mediated insertion of membrane proteins, for instance, the a and b subunits of the F_1_F_0_ ATPase and TatC (92). *Streptococcus mutans* contains two YidC paralogs with YidC1 having a shorter C-terminal part compared to YidC2. Interestingly, YidC1 was required for full zinc resistance in this bacterium (94). The deletion in YidC of the ∆e5 strain might thus compensate for the increased zinc sensitivity of this quintuple deletion mutant; however, it should not influence metal import or efflux reactions if YidC is not involved in the insertion of the respective transport protein into the cytoplasmic membrane.

*C. metallidurans* possesses 11 sigma factors of the extracytoplasmic sigma factor family, ECF (95–100). Among these, there is one founding member of the family (101), CnrH, which is involved in expression of the plasmid pMOL28-mediated *cnr* determinant (79–81, 102). Mutants of *C. metallidurans* carrying triple or double deletions of the genes for related ECF sigma factors demonstrated a clear phenotype that allowed an association of groups of sigma factors to particular cellular processes, for instance, managing homeostasis of other transition metal cations despite a strong preference for iron import (14). On the other hand, none of these sigma factors was essential for the expression of even one single gene (14), although CnrH was important for *cnr* expression (79, 81, 102), and RpoI was essential for the synthesis of the siderophore of this bacterium (99). Some compensatory mechanisms allowed the expression of known target genes in the sigma factor mutants (14). No SNPs were, however, found in the upstream regions of target genes such as *cnr* or siderophore biosynthesis genes. The number of SNPs in the genomes of the sigma factor mutants was low (Fig. 10) and not connected to promoter regions, genes for other sigma factors, or the RNA polymerase.

Only in one case, a possible connection between a sigma factor mutant and a SNP was evident. Strain DN546 carries a deletion in the genes for sigma factors RpoQ and RpoR. Its phenotype was associated with copper and thiol homeostasis (14). The *rpoQ* gene and that encoding its anti-sigma factor are in the vicinity of the *gig* operon, which is involved in copper resistance of *C. metallidurans* and is upregulated by gold (44, 88, 103–105). This operon encodes bufferins, ribosomally synthesized and post-translationally modified peptides, long overlooked as contributors to copper resistance (89). Mutant DN546 carried a SNP in the gene for the main periplasmic copper-dependent copper oxidase CopA1. This SNP may decrease the number of Cu(I) ions bound to a copper-storage domain of CopA1 (Fig. S10). Due to the contribution of CopA1 to the multi-factor copper homeostasis system in *C. metallidurans* (88), this should mediate only a mild change in copper homeostasis of this bacterium. However, this SNP does not explain why RpoQ is not essential for *gig* expression.

In the case of SNPs with high variant abundance, this means that probably none of the experimental results obtained with *C. metallidurans* mutant cells was affected by the secondary mutations, except the SNPs in the ∆9 mutants, which clearly demonstrated the effect of a suppression in pulse-chase experiments (18). Moreover, the compensatory effect observed in the sigma factor mutants was not associated with SNPs, but is likely to be based upon another mechanism.

### The hypervariable regions

Hypervariable regions were clusters of adjacent polymorphisms with low variant frequencies, usually surrounding one or two SNPs with frequencies > 50% (Fig. 6 to 8). Because all SNPs with variant frequencies > 50% were associated with regions of low coverage, for example, at the edge of deletions or assigned to genes with many paralogs and which were excluded from further considerations, the observed hypervariable regions could not be considered annotation artifacts caused by the respective factors.

Twelve hypervariable regions were identified: (i) in *Rmet_1656* or *yhjG* for a glutathione-S-transferase-like protein in the ∆7 uptake mutant; (ii) *Rmet_3071* for a histone H1-like DNA-packaging protein in ∆e4 ∆*zupT*; (iii) in the gene for the PitA import system Rmet_1973 of metal phosphate complexes in ∆e6; (iv) upstream of the *murI* gene *Rmet_2274* for a glutamate racemase important for synthesis of D-glutamate for the peptide cross-link of the bacterial peptidoglycan in four mutants with deletions in the genes for FolE_I-type GTP cyclohydrolases; (v) upstream of *slyB* in the ∆*folE* and ∆*cobW* double mutants; (vi) *Rmet_2164* encoding a KAP-P-loop NTPase in ∆*folE* mutants; (vii) *Rmet577* or *betB* for an aldehyde dehydrogenase; (viii) *Rmet_3361* and (ix) *Rmet_5469* for uncharacterized proteins, all three in CH34_10; (x) *Rmet_4700* for a conserved protein with a signal peptide in CH34_1; and (xi and xii) two different regions in *zur* in ∆*folE* double mutants. Hypervariable regions occurred frequently in *C. metallidurans* mutants, especially in those with multiple deletions or with a strong impact of the single or double deletion on the overall metabolism.

Comparison of 10 hypervariable regions within open reading frames (Fig. 8) revealed that hypervariable regions are clustered around one or two SNPs in a region of up to 70 base pairs with a variant frequency between 50% and 85%. None of the SNPs with a high variant frequency exhibited such a cluster of accompanying SNPs with low variant frequencies (shown for *yidC* in Fig. 8). In the case of *pitA*, the two central SNPs either grouped together with the SNPs upstream or downstream of them. This indicates that about half of the sequencing templates contained the two central SNPs, half again of those together with upstream SNPs that decrease from 45% to 25% in variant frequency with the distance to the central SNPs, and the other 25% with downstream SNPs with a similar distance-frequency pattern (Fig. 6H). This gives the impression that the central SNPs occurred first and were subsequently expanded in the upstream or the downstream direction.

The two hypervariable regions in *zur* that were identified in different ∆*folE* double mutants were at different positions in *zur*, one at the 5′ end and one within the open reading frame close to the 3′ end of the gene (Fig. 7). This indicated that hypervariable regions occurred at random places within, or upstream of, genes.

The hypervariable region in *zur* in the two different ∆*folE* double mutants had a clear phenotype. In the parent strains, the *cobW1* operon is under strict control of Zur (32, 33). In the ∆*folE_IA ∆folE_IB1::I* mutant, the *cobW1* operon was expressed, and the products of this operon were identified in the proteome (Fig. 9). The proteome includes the third FolE protein FolE_IB2, which now allows folate biosynthesis in the mutant cells, whereas in the parent cells, this protein was not present. The hypervariable region in the ∆*folE_IA ∆folE_IB1::I* mutant could grow due to the SNPs in the hypervariable region. It should be noted that the single mutant displayed an upregulation of the copy number of GcvH. Since the respective gene carries the ZMP/ZTP-*pfl* riboswitch upstream (30), this indicates a down-regulated GTP synthesis in these mutants due to the lack of folate.

The central SNP in the *zur* gene of the ∆*folE_IA ∆folE_IB1::I* mutant had a variant frequency of about 55%, indicating that some Zur protein should still be present in the cells. Compared to the parent strain AE104, the Zur content of the double mutant was 38%, but this value was not significant due to a high level of deviation. Zur was identified using three peptides (Table 3; Fig. 7A, black, red, and blue boxes). Peptide 1 at the N-terminus (black box) was found twice and with a much lower abundance compared to that of peptide 1 in the single mutants and the parent strain. Peptide 2, just upstream of the amino-acyl residues affected by the SNPs in the hypervariable region (red box), was found just once with a high technical deviation of the measurement, and peptide 3 (blue box), within the mutated region, also occurred only once and with a comparably low abundance. Some residual Zur content was found in the mutant, but it could not be stated whether this was wild-type Zur or a mutant protein. In contrast, in the other double mutant ∆*folE_IB1 ∆folE_IA::I*, which had the SNPs upstream of *zur* or its 5′ end (Fig. 7A), Zur was found only as peptide 3 in just one example (Table 3). This did not exclude a low copy number of the Zur protein in the cells of this mutant, and also did not prove this fact.

In both ∆*folE* double mutants and other examples of mutants containing hypervariable regions (Fig. 8), many variable sequence reads were annotated to the respective target gene. This indicates the existence of different sequencing templates in the cell sample used for genomic sequence analysis. This may indicate a variety of sub-populations with different target gene sequences or copies of the respective gene within the cells, for instance, if *C. metallidurans* exhibited polyploidy (106).

This means that hypervariable regions were not an annotation artifact; they occurred frequently in *C. metallidurans* mutants and at random positions of the affected gene. Moreover, they resulted in a phenotype. In the case of *zur*, the mutations clustered as a hypervariable region allowed the cells to grow because folate biosynthesis was again possible in the respective *folE* double mutant, causing a suppression of the effect of the two introduced mutations. Due to the absence of FolE_IA and FolE_IB1, plus repression of synthesis of FolE_IB2 by Zur, initiation of folate biosynthesis is no longer possible after introduction of the second ∆*folE* deletion, which could only be realized as an interruption (30, 62, 107–110). Only cells with mutations in *zur* or in the promoter region of the *cobW1* operon would be able to produce FolE_IB2 and subsequently synthesize folate and so be able to grow. The corresponding mutations were realized as hypervariable regions in *zur* based on *zur* variants in the population of the double mutant strains or within a bacterial cell exhibiting polyploidy. Sub-populations that are unable to produce folate due to the repression of *folE_IB2* transcription by Zur would only be able to grow if they received folate from the cells of another, *folE_IB2*-expressing sub-population, which would require a folate import and export system in the respective cells. On the other hand, polyploid cells might contain wild type and mutated *zur* genes in the same cell, which would lead to some expression of *folE_IB2* and subsequently folate synthesis. Consequently, Ockham’s razor would favor the “polyploidy” hypothesis.

The FolE_IB2-producing mutant cells contained only one hypervariable region, as indicated by at least one SNP with a variant frequency above 50%. These are not the result of a loss of the mismatch repair system that leads to hypermutation in the cell line because, in that case, more hypervariable regions should have been observed or indeed a higher number of SNPs in general (111). Instead, they are reminiscent of localized hypermutation events observed in evolving bacterial populations and could represent variations of the mechanisms described here (112).

## MATERIALS AND METHODS

### Bacterial strains and growth conditions

Strains used for experiments were derivatives of the plasmid-free derivative AE104 or *C. metallidurans* CH34 wild type (1) and are listed in Table S2. Tris-buffered mineral salts medium (1) containing 2 g sodium gluconate/L (TMM) was used to cultivate these strains aerobically with shaking at 30°C. TMM with different zinc concentrations was used, as described (31). Tris-buffered media were solidified by inclusion of 20 g agar/L. Strains were routinely transferred to fresh TMM plates every 2 weeks and taken from the −80°C stock culture twice a year.

### Genetics, measurements, determination of resistance, and reporter genes

Dose-response growth curves in 96-well plates, construction of reporter genes, ß-galactosidase assays, gene deletions, disruptions, construction of reporter operon fusions, and other genetic methods, including PCR, were performed exactly as described in previous studies (30, 31). Primers are listed in Table S3. Proteomics was done and analyzed as previously published (15).

### Statistics

Students’ *t*-test was used, but in most cases, the distance (D) value has been used several times previously for such analyses (23, 105, 113). It is a simple, more useful value than Student’s *t*-test because non-intersecting deviation bars of two values (D > 1) for three repeats always means a statistically relevant (≥ 95%) difference, provided the deviations are within a similar range. At *n* = 4, significance is ≥ 97.5%; at *n* = 5, significance is ≥ 99%; and at *n* = 8, significance is ≥ 99.9% (highly significant).

### Re-sequencing

As published previously (13), genomic DNA was isolated using the Genomic DNA Purification Kit (Thermo Scientific) according to the manufacturer’s instructions and eluted with 100 µL ddH_2_O. DNA concentration was determined using an IMPLEN N60 NanoPhotometer, and the quality was checked on agarose gels (42). From extracted genomic DNA, next-generation sequencing libraries were generated using a modified Illumina Nextera library kit protocol (114), and libraries were sequenced in a 2 × 150 bp or 2 × 300 bp paired-end run on the Illumina NextSeq 500 or MiSeq instrument, respectively (Illumina, San Diego, CA, USA) for the genomic sequence runs 1–21. Due to the continually increasing efficiency and accuracy of the next-generation sequencing protocols, hardware, and software, the more recently performed runs 29–46 were double runs on just one instrument, 29–33 on a MiSeq, and 36–46 on a NexSeq (Fig. 10). Those numbers not listed in Fig. 10 are bacterial strains and species not used for this publication.

The genomic sequences were evaluated using Geneious 2025.0.2 (115). The reads were trimmed using the BBduk algorithm of Geneious, duplicates were removed, and the reads were assembled to the genome of *C. metallidurans* CH34 (Version CP000352.1). SNPs and regions of low coverage of the re-sequenced *C. metallidurans* CH34 genome and the mutant strains were identified in comparison to the CH34 gene bank entry in the MiSeq or NextSeq double runs in all replicons. A region was identified as being of low coverage when its coverage was below the mean value of the coverage of the replicon sequence minus two standard deviations.

### Data curing

When double runs on the MiSeq and NexSeq instruments were available (runs 1–21, Fig. 10), SNPs were further considered that were present in the MiSeq and NextSeq double reads with variant frequencies > 50%, present in both double reads, and additionally possessed a mean variant frequency over all runs > 90%. Additionally, SNPs with variant frequencies between 50% and 90% were not discarded when they were (i) outside of regions of low coverage; (ii) not flanking large deletions; and (iii) not annotated to genes having multiple paralogs, for example, the transposase *tnpB* or the replication initiator *repA*. When double runs of a genome were only available from one sequencing instrument (runs 29–46), also the groups of “almost certain SNP” with a variant frequency of > 90% and that of “possible SNPs” with frequencies between 50% and 90% were formed, provided that they are outside of regions of low coverage, not in flanking regions, and not annotated to genes with multiple paralogs.

SNPs already discussed in the respective parent strains (Fig. 1) were not mentioned when the mutant derivatives were analyzed. Almost all certain mutations were also present in the derivatives, but not all possible mutations. When the overall number of mutations was analyzed, mutations in sister strains were also counted as one mutation (Table 1). The supplement “overview mutations” contains all these mutations, divided into those outside of open reading frames, within genes, and expressed in *C. metallidurans* CH34 wild type under non-challenging conditions (12, 72). Moreover, the mutations were inserted into the transcriptional landscape of the four replicons, which forms the connection between the abundance of transcripts of genes, upstream and downstream regions, plus the abundance of transcriptional start points.

## Data Availability

Re-sequenced genomic and proteomic data were deposited with the Bioproject accession PRJNA769585 at NCBI.

## SUPPLEMENTAL MATERIAL

File S1 jb.00121-26-s0001.xlsx All the identified mutations are listed and categorized.

File S2 jb.00121-26-s0002.xlsx All mutations are embedded into the transcriptional landscape of the four replicons.

File S3 jb.00121-26-s0003.xlsx This contains the proteomic data of a double mutant.

Supplemental materials jb.00121-26-s0004.pdf Supplementary figures, tables, and an extended Result part.

ASM does not own the copyrights to Supplemental Material that may be linked to, or accessed through, an article. The authors have granted ASM a non-exclusive, world-wide license to publish the Supplemental Material files. Please contact the corresponding author directly for reuse.
