# Supplementary material for: How mutagenesis and laboratory conditions affect the genome of the metal-resistant bacterium Cupriavidus metallidurans strain CH34
Source: J Bacteriol. 2026 Apr 29;208(5):e00121-26. doi: 10.1128/jb.00121-26 (PMC13192273; doi:10.1128/jb.00121-26)
Supplement: Supplemental materials — Supplementary figures, tables, and an extended Result part. [file jb.00121-26-s0004.pdf]

Supplement

Supplementary Figure S1. Screenshots of the Geneious analysis of *C. metallidurans* genomes. 2
EXTENDED RESULTS 3
Genome stability. 3
Supplementary Figure S2. Fusion of gene fragments by two frame-shift mutation creates a full-length *merA* gene. 4
Supplementary Figure S3. Alignment of the translated product of a fused open reading frame that results from a C-insertion at position 3620762 of the chromosome. 5
Supplementary Figure S4. Verification of the presence or absence of genes in strain CH34_10 compared to CH34 wild type 7
Supplementary Figure S5. Predicted secondary structure of the intergenic region between *mreB* and *mreC* on the chromosome of C. metallidurans. 8
Supplementary Figures S6. Comparison of metal resistance of strains CH34 and CH34_10. 9
Supplementary Figure S7. Emerging, nearly complete and complete deletions type A, B and C in strains AE104, AE126 and AE126_10. 10
Supplementary Figure. S8. Metal resistance of a spontaneous mutant of C. metallidurans AE104. 11
Supplementary Figure S9. No trace for a beginning deletion type B in two mutant strains of AE104. 12
Sigma factors. 12
Supplementary Figure S10. CopA1 wild type (Panel A) and mutant strain DN546(Panel B). 14
Uptake systems. 14
Supplementary Figure S11. Comparison of the activity of the parent and mutated *rpoH* promoter. 15
Efflux systems. 18
Supplementary Figure S12. Model of the TetR-like regulatory protein Rmet_4723. 20
Zur regulon components. 21
Supplementary Figure S13. Hypervariable regions in *slyB* and Rmet_2164. 24
Folate biosynthesis. 26
Supplementary Figure S14. Structure of UvrB wild type and its D14G mutant. 28
FEATURES OF THE LARGE DELETIONS 29
Deletions on the chromosome 29
Deletions on plasmid pMOL28 30
Deletions on the chromid 30
OTHER SUPPLEMENTARY MATERIAL 33
Supplementary Table S1. Changes in the proteome of ΔfolE mutants 33
Supplementary Table S2. Bacterial strains. 36
Supplementary Table S3. Primers. 37
SUPPLEMENTARY EXCEL FILES 38
LITERATURE OF THE SUPPLEMENT 38

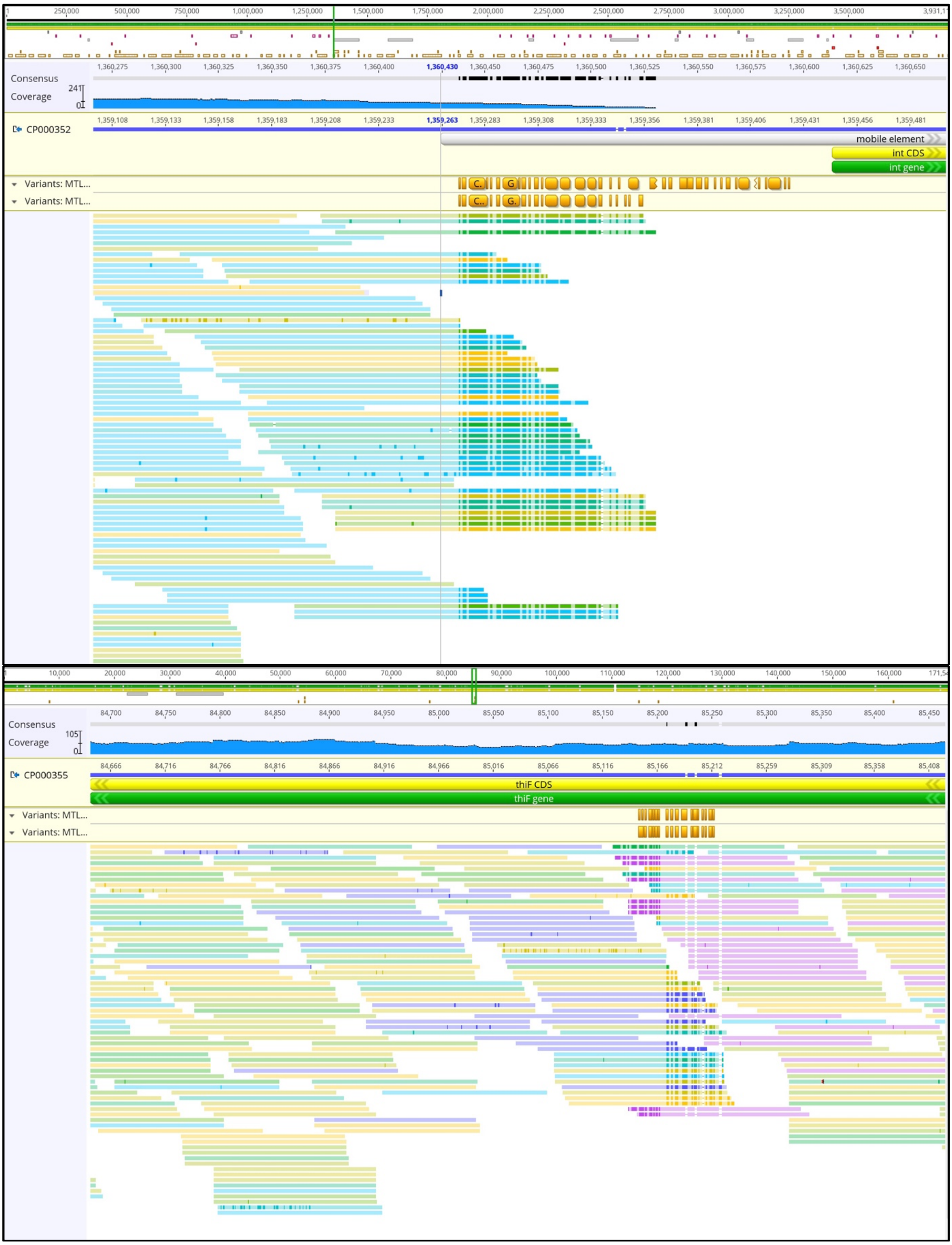

Supplementary Figure S1. Screenshots of the Geneious analysis of *C. metallidurans* genomes. The top shows artificial SNPs indicated by Geneious (yellow fields in the variant lines) at the beginning of the deleted genomic island GMGI-2. The sequence reads (bars below) stemming from strain CH34_1 were mis-assembled to the CH34 genome because strain CH34_1 carries the deletion of this genomic island. Bottom, similar artificial SNPs in a region with low coverage.

Extended Results

Genome stability. *C. metallidurans* CH34 wild type was maintained under constant selection pressure alternating between 1 mM Zn(II) for the presence of plasmid pMOL30 and 1 mM Ni(II) for pMOL28 and refreshed from stock culture regularly. It contained all genomic islands, plasmids and no deletion (Table 1). Five differences between the genomes of the newly sequenced and the published genome (1) were found, three insertions into chromosomal genes leading to a frame shift, one intergenic insertion on the chromosome and an intergenic transversion on the chromid. These SNPs were present in all derivatives of strain CH34 (Fig. 1). Two insertions into the chromosome resulted in frame-shifts that fused two fragments of a *merA* gene fragment (Rmet_2315) for a mercury reductase into a gene coding for a full-length MerA protein that was 78% identical to a plasmid-encoded ortholog from *E. coli* (Suppl. Fig. S2). Another insertion fused the reading frame locus tag Rmet_1728 and a later annotated Rmet_6474 downstream into a full-length gene that encoded a transmembrane protein of the major facilitator superfamily predicted to be a proton metabolite symporter. The deduced protein sequence was 99% identical to proteins in other *Cupriavidus* strains (WP_017513852.1). Another insertion into an intergenic region led to a frame-shift upstream of the open reading frame (orf) Rmet_3343, which increased the size of the orf, yielding a 702 aa long membrane protein. A BLAST search (2) indicated that this protein was identical to three predicted proteins in other *C. metallidurans* strains (Suppl. Figure S3). The transversion on the chromid was 14 bp downstream from a transcriptional start site. It may influence a riboswitch, the interaction with a *trans* regulatory small RNA, or have no effect.

All changes on the chromosome resulted in open reading frames encoding proteins with a high similarity to orthologs in other bacteria. Since the probability is low that a random mutation creates a fully functional gene from an inactivated one, the four SNPs on the chromosome of *C. metallidurans* wild type were probably caused by reading errors in the originally published (1) genomic sequence, highlighting the increase in sequence fidelity during the last two decades. Most important, it demonstrated that the genome of *C. metallidurans* CH34 wild type was stable when kept under appropriate conditions.

Mutant CH34_1 of strain CH34 originated spontaneously and was different from the wild type strain in several respects, e.g. a larger cellular diameter and a round-cell morphology (3). It contained the two large deletions A and B on the chromosome, deletion C in the chromid and D on plasmid pMOL28 (3). The type D deletion in plasmid pMOL28 removed the *chr-cnr* metal resistance determinants and was unique to strain CH34_1 (Table 1).

```
Consensus                          MAEAITLHIEGMTCTSCAEHVQQALTNVPGVRAASVSYPQRQAEIEADAGVSVAPLVAAV   60
CP000352 - merA' CDS translation   MAEAITLHIEGMTCTSCAEHVQQALTNVPGVRAASVSYPQRQAEIEADAGVSVAPLVAAV   60
CP000352 - merA'' CDS translation  ------------------------------------------------------------
MerA_Ecoli                         ---MSTLKITGMTCDSCAVHVKDALEKVPGVQSADVSYAKGSAKLAIEVGTSPDALTAAV   57
MerA1-MerA2 translation            MAEAITLHIEGMTCTSCAEHVQQALTNVPGVRAASVSYPQRQAEIEADAGVSVAPLVAAV   60

Consensus                          ATLGYRARLTDTPN--KPAGLLDKALGWLGGETKHVGGEQALHVAVIGSGGAAMAAALKA  118
CP000352 - merA' CDS translation   ATLGYRARLTDTPN--KPAGLLDKALGWLGGETKHVGGEQALHVAVIGSGGAAMAAALKA  118
CP000352 - merA'' CDS translation  ------------------------------------------------------------
MerA_Ecoli                         AGLGYRATLADAPSVSTPGGLLDKMRDLLGRNDK-TGSSGALHIAVIGSGGAAMAAALKA  116
MerA1-MerA2 translation            ATLGYRARLTDTPN--KPAGLLDKALGWLGGETKHVGGEQALHVAVIGSGGAAMAAALKA  118

Consensus                          VEQGARVTLIERGTIGGTCVNVGCVPSKIMIRAAHIVHLRRESPFDAGLPAAAPAVLRER  178
CP000352 - merA' CDS translation   VEQGARVTLIERGTIGGTCVNVGCVPSKIMIRAAHIVHLRRESPFDAGLPAAAPAVLRER  178
CP000352 - merA'' CDS translation  ------------------------------------------------------------
MerA_Ecoli                         VEQGARVTLIERGTIGGTCVNVGCVPSKIMIRAAHIAHLRRESPFDGGIAATTPTIQRTA  176
MerA1-MerA2 translation            VEQGARVTLIERGTIGGTCVNVGCVPSKIMIRAAHIVHLRRESPFDAGLPAAAPAVLRER  178

Consensus                          LLAQQQGRVEELRHAKYEGILASTPAITVLRGEARFRDTRTLTVATADGGTHEVNFDRCL  238
CP000352 - merA' CDS translation   LLAQQQGRVEELRHAKYEGILASTPAITVLRGEARFRDTRTLTVATADGGTHEVNFDRCL  238
CP000352 - merA'' CDS translation  ------------------------------------------------------------
MerA_Ecoli                         LLAQQQARVDELRHAKYEGILEGNPAITVLHGSARFKDNRNLIVQLNDGGERVVAFDRCL  236
MerA1-MerA2 translation            LLAQQQGRVEELRHAKYEGILASTPAITVLRGEARFRDTRTLTVATADGGTHEVNFDRCL  238

Consensus                          IATGASPALPPIPGLADTPHWTSTEALESSSLPERLAVIGSSVVAVELAQAFARL---GA  295
CP000352 - merA' CDS translation   IATGASPALPPIPGLADTPHWTSTEALESSSLPERLAVIGSSVVAVELAQAFARR---AA  295
CP000352 - merA'' CDS translation  ------------------------------------------MVPPWWRSSWRKPSPGGQ   18
MerA_Ecoli                         IATGASPAVPPIPGLKDTPYWTSTEALVSETIPKRLAVIGSSVVALELAQAFARL---GA  293
MerA1-MerA2 translation            IATGASPALPPIPGLADTPHWTSTEALESSSLPERLAVIGSSVVAVELAQAFARL---GS  295

Consensus                          XVTILAXSTLFFREDPAIGEAVTDAFRAEGIEVLDHTQASHVAY----XGGEFVLTTXQG  351
CP000352 - merA' CDS translation   RSRSWRAARCSSEKTGHRGSRNRRLPRRGHRGAGPHPGEPRCLC----GRGIRAHHRAGG  351
CP000352 - merA'' CDS translation  PGHDPGAQHAVLPRRPAIGEAVTDAFRAEGIEVLDHTQASHVAY----AGGEFVLTTGQG   74
MerA_Ecoli                         KVTILARSTLFFREDPAIGEAVTAAFRMEGIEVREHTQASQVAYINGEGDGEFVLTTAHG  353
MerA1-MerA2 translation            QVTILARSTLFFREDPAIGEAVTDAFRAEGIEVLDHTQASHVAY----AGGEFVLTTGQG  351

Consensus                          EVRADKLLVATGRAPNTRSLNLEAAGVEVNAQGAIVIDRAMRTSAPHIFAAGDCTDQPQF  411
CP000352 - merA' CDS translation   SARRQAAGRHRSRAEHAQPEP---------------------------------------  411
CP000352 - merA'' CDS translation  EVRADKLLVATGRAPNTRSLNLEAAGVEVNAQGAIVIDRAMRTSAPHIFAAGDCTDQPQF  134
MerA_Ecoli                         ELRADKLLVATGRAPNTRKLALDATGVTLTPQGAIVIDPGMRTSVEHIYAAGDCTDQPQF  413
MerA1-MerA2 translation            EVRADKLLVATGRAPNTRSLNLEAAGVEVNAQGAIVIDRAMRTSAPHIFAAGDCTDQPQF  411

Consensus                          VYVAAAAGTRAAINMTGGDAALDLTAMPAVVFTDPQVATVGYSEAEAHHDGIETDSRLLT  471
CP000352 - merA'' CDS translation  ------------------------------------------------------------  471
CP000352 - merA'' CDS translation  VYVAAAAGTRAAINMTGGDAALDLTAMPAVVFTDPQVATVGYSEAEAHHDGIETDSRLLT  194
MerA_Ecoli                         VYVAAAAGTRAAINMTGGDAALNLTAMPAVVFTDPQVATVGYSEAEAHHDGIKTDSRTLT  473
MerA1-MerA2 translation            VYVAAAAGTRAAINMTGGDAALDLTAMPAVVFTDPQVATVGYSEAEAHHDGIETDSRLLT  471

Consensus                          LDNVPRALANFDTRGFIKLVAEAGSGRLIGVQAVAPEAGELIQTAALAIRHRMTVQELAD  531
CP000352 - merA' CDS translation   ------------------------------------------------------------  531
CP000352 - merA'' CDS translation  LDNVPRALANFDTRGFIKLVAEAGSGRLIGVQAVAPEAGELIQTAALAIRHRMTVQELAD  254
MerA_Ecoli                         LDNVPRALANFDTRGFIKLVVEEGSGRLIGVQAVAPEAGELIQTAALAIRNRMTVQELAD  533
MerA1-MerA2 translation            LDNVPRALANFDTRGFIKLVAEAGSGRLIGVQAVAPEAGELIQTAALAIRHRMTVQELAD  531

Consensus                          QLFPYLTMVEGLKLAAQTFNKDVKQLSCCAG                              562
CP000352 - merA' CDS translation   -------------------------------                              372
CP000352 - merA'' CDS translation  QLFPYLTMVEGLKLAAQTFNKDVKQLSCCAG                              285
MerA_Ecoli                         QLFPYLTMVEGLKLAAQTFNKDVKQLSCCAG                              564
MerA1-MerA2 translation            QLFPYLTMVEGLKLAAQTFNKDVKQLSCCAG                              562
```

Supplementary Figure S2. Fusion of gene fragments by two frame-shift mutation creates a full-length *merA* gene. The figure shows an alignment of the translated *merA’* and *merA”* genes on the chromosome of *C. metallidurans* with MerA from *E. coli* (QHJ90100.1) and translation of a gene that results from two SNPs found in this region. Peptide parts not related to the *E. coli* protein are shown in red. The amino acid residues in green show the regions present in the fused protein, and underlined blue shows the region between the frame-shift mutations.

```
Sequence ID: WP_029306892.1 Length: 702
>FUSC family protein; Cupriavidus metallidurans
Identities:702/702(100%), Positives:702/702(100%), Gaps:0/702(0%)

Query  1    MRPALHYLRYWPDAAFLRRGAVYALSFLTLAGTAWLTGDTGYIWSATASIWTCLADRPGT  60
Sbjct  1    MRPALHYLRYWPDAAFLRRGAVYALSFLTLAGTAWLTGDTGYIWSATASIWTCLADRPGT  60

Query  61   AAARIRGLATVGIGGAAVSVIGASLHASPLAALAFVLAAGLMAGLSEVRGPATALWFKLL  120
Sbjct  61   AAARIRGLATVGIGGAAVSVIGASLHASPLAALAFVLAAGLMAGLSEVRGPATALWFKLL  120

Query  121  YVVLIAACLQPASGPSASAHAWMAGLDFLRGGLFACAVSLVLIPSDRETRPRTEIIAIYD  180
Sbjct  121  YVVLIAACLQPASGPSASAHAWMAGLDFLRGGLFACAVSLVLIPSDRETRPRTEIIAIYD  180

Query  181  ALRRFAVALAEAGSLDMTPHKQEIRLCIETARRALASRRGLADPVALVHYAYAIAVGDAI  240
Sbjct  181  ALRRFAVALAEAGSLDMTPHKQEIRLCIETARRALASRRGLADPVALVHYAYAIAVGDAI  240

Query  241  FALLIVAGELRERLGVGHALPLACAASRLTDIHAQVLQALSCHGPDLPALTAVLFRDLRE  300
Sbjct  241  FALLIVAGELRERLGVGHALPLACAASRLTDIHAQVLQALSCHGPDLPALTAVLFRDLRE  300

Query  301  LAARRTHASTPPAYQSALAALAQFPAFDRWREGFSWPNSGFAGLIDRLGLMLADLAARDT  360
Sbjct  301  LAARRTHASTPPAYQSALAALAQFPAFDRWREGFSWPNSGFAGLIDRLGLMLADLAARDT  360

Query  361  RVTRHSVRLALAGGLSLLPAQIWHVDHGYWVAVTVIMVLSPRLQTTRQISFLRFAGSLAG  420
Sbjct  361  RVTRHSVRLALAGGLSLLPAQIWHVDHGYWVAVTVIMVLSPRLQTTRQISFLRFAGSLAG  420

Query  421  ALLACAISLWHPTPAPVLGLSALFLASAYAARLAGNPAGFAFCLTPAVILFSWLGEPTSS  480
Sbjct  421  ALLACAISLWHPTPAPVLGLSALFLASAYAARLAGNPAGFAFCLTPAVILFSWLGEPTSS  480

Query  481  SSQVAAMRGLDTAIGCLIALASYYILAPRAELSRVFRHSIDALAVNAVYLRAASRSSRTL  540
Sbjct  481  SSQVAAMRGLDTAIGCLIALASYYILAPRAELSRVFRHSIDALAVNAVYLRAASRSSRTL  540

Query  541  TPSHLRLEALRVAAGRASSRAEATLSQSAGDLASDLTAAHTSLHDTARRMASLAGLIRAG  600
Sbjct  541  TPSHLRLEALRVAAGRASSRAEATLSQSAGDLASDLTAAHTSLHDTARRMASLAGLIRAG  600

Query  601  AESGSVNEHPGPAVQAMLSELEVRLAEVAARPGRIDAIAPSAPDRIGPSPLATTAFEQFL  660
Sbjct  601  AESGSVNEHPGPAVQAMLSELEVRLAEVAARPGRIDAIAPSAPDRIGPSPLATTAFEQFL  660

Query  661  VEQAAYANAHVDSAHQAVARMSALASAHEHGGRSRRGSLHAS  702
Sbjct  661  VEQAAYANAHVDSAHQAVARMSALASAHEHGGRSRRGSLHAS  702
```

Supplementary Figure S3. Alignment of the translated product of a fused open reading frame that results from a C-insertion at position 3620762 of the chromosome. The additional N-terminal sequence is underlined. The enlarged predicted protein is identical to those found in the *C. metallidurans* strains FDAARGOS_675, NA1 and NA4.

Deletion A removed the genomic island CMGI-2 from the chromosome of strain CH34_1. Region A harbored the genes for the membrane-bound hydrogenase, B the genes for the Calvin cycle enzymes and the soluble, NAD^+-reducing hydrogenase on the chromosomal island CMGI-3 (3, 4). The type C deletion, present in most mutants except one (Table 1), removed the *caiB* gene for a L-carnithine dehydratase and Rmet_5544 for an aldehyde dehydrogenase, as already noted (3). Both genes were downstream of Rmet_5545 for an alcohol dehydrogenase, upstream of transposase genes and not expressed under any tested

condition (5). The genome of strain CH34_1 with its altered cell morphology contained 9 SNPs, five of them possible mutations. A silent mutation appeared in the *gltB* gene for the glutamate synthase and the other four mutations in genes for non-expressed hypothetical or uncharacterized genes. The SNPs in intergenic regions also did not lead to insights concerning the phenotype of this strain (Suppl. Overview mutations.xlsx).

Strain CH34_1 was kept for more than 10 years with monthly transfers onto Tris-buffered mineral salts medium plates without selection pressure. In none of the monthly transfers were revertants with the cell morphology of the wild type observed. Strain CH34 wild type was also kept under the same conditions, without selection pressure or revival from a stock culture, leading to strain CH34_10 (Fig. 1). This strain never showed cells with the round morphology of CH34_1 during a monthly transfer. Strain CH34_10 lost the regions B, C and a part of region A, but none of the plasmids (Table 1). This was also verified by PCR (Suppl. Fig. S4).

Additionally, CH34_10 carried the deletions I and R. The chromidal deletion I comprised the gene encoding a H-NS-type DNA-binding protein, along with R pilus-assembly genes and a part of the recessive and partly inactivated *nim* cluster for a transenvelope efflux system. The CH34_10 genome contained 15 SNPs, 13 within genes. Among these were 7 possible mutants in expressed genes, two of these transversions in Rmet_1577 for an aldehyde dehydrogenase. The other 6 polymorphisms (4 certain and 2 possible mutations) were in weakly or non-expressed genes encoding a periplasmic substrate-binding protein of an ABC import system, a histidine kinase, benzoate-CoA ligase and a universal stress protein, UspA. Two mutations were in the intergenin region between the genes for the rod shape-determining proteins MreB and MreC. The predicted secondary structure of this region (Suppl. Fig. S5) demonstrated that these mutations would lead to an improvement of a large stem-loop structure in strain CH34_10. A homology search revealed matches of the sequence CCCCCATCTTGAAC between two of the stem-loop structures with three genes, Rmet_2030 (*rbfA*, 13/14 nucleotides matched, 30S ribosome binding protein), Rmet_2430 (*fabH*, 12/14, 3-oxoacyl-{acyl-carrier protein} synthase) and Rmet_5849 (*epsB*, 12/14, tyrosine protein kinase). These may be targets of a possible sRNA encoded between *mreB* and *mreC*. Alternatively, the region may control expression of the genes downstream of *mreB* in the rod-shape operon Op0017f_1, *mreBCD-mrdAB* (6). This operon is two-fold down-regulated in metal-stressed CH34 cells. While the anti-sense RNA of *mreC* is 55-fold up-regulated, those of the *mreD-mrdAB* genes are 2 to 4-fold down-regulated (5). Strain CH34_10 was no longer under metal stress and the mutations may allow it to maintain its rod-shape during growth under non-challenging conditions. Interestingly, these two mutations were present in strain CH34_10 with its rod form but not in strain CH34_1 with its round cells.

When metal resistance of CH34_10 was compared with that of CH34 (Suppl. Fig. S6), zinc resistance between both strains was similar. CH34_10 clearly lost some resistance to Co(II) and Ni(II) (Suppl. Fig. S6A). This indicated that the regions A, B, and C or the recessive and inactivated *nim* determinant may contribute to full cobalt and nickel resistance in *C. metallidurans* CH34.

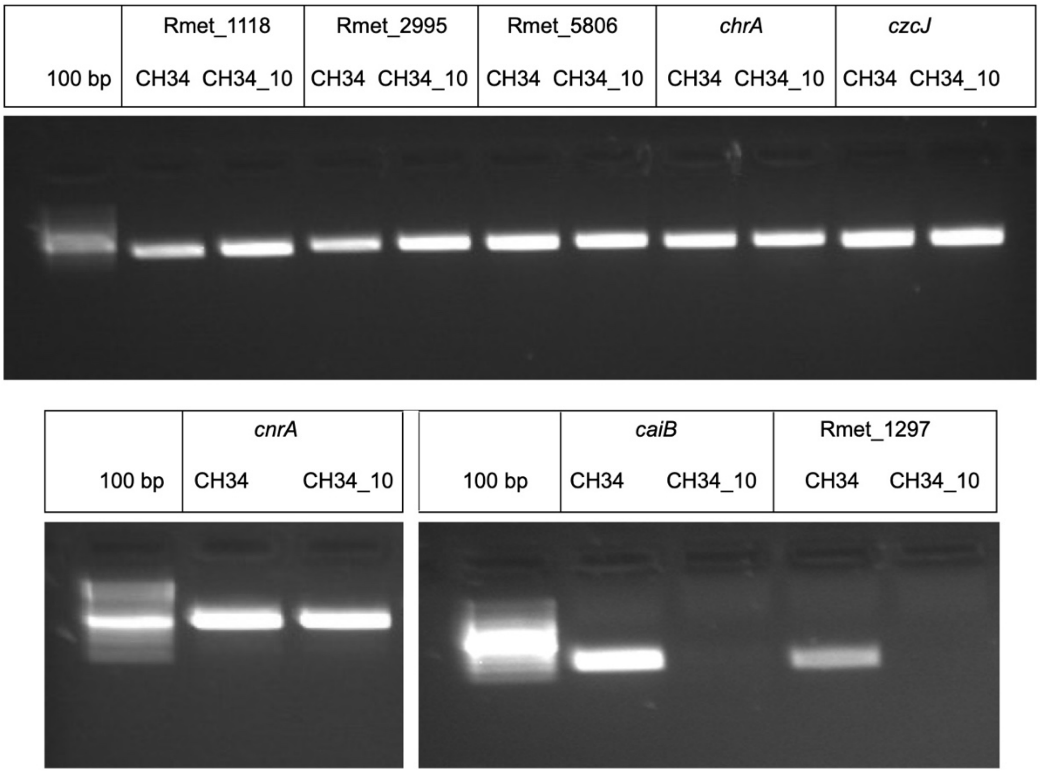

Supplementary Figure S4. Verification of the presence or absence of genes in strain CH34_10 compared to CH34 wild type. A PCR was done with DNA isolated from both strains. It demonstrated the presence of chromosomal (Rmet_1118, Rmet_2995), chromid (Rmet_5806), pMOL30 (*czcJ*) and pMOL28 (*chrA, cnrA)* genes but deletion of Rmet_1297 (type A deletion) and of *caiB* (type C deletion).

The genome of strain CH34 remained stable when kept under appropriate conditions but accumulated mutations and lost regions when on a plate without selection pressure. The plasmid-free mutant AE104 contained all regions except the *caiB*-region C on the chromid when revived regularly from frozen stock. Since this strain is no longer metal resistant, selection for plasmid-encoded metal resistance could not be applied. After 10 years on agar plates without selection pressure, strain AE104_10 lost the regions A, B, C and E. Deletion E comprises genomic CMGI-4 except transposon Tn*6048*, which is located within it. The deleted region encodes genes for a methylene-tetrahydrofolate reductase, a methionine biosynthesis regulator MetR and the *hmzBA-hmzRS* interrupted region for a non-expressed transenvelope system and its two-component regulators (Table 1).

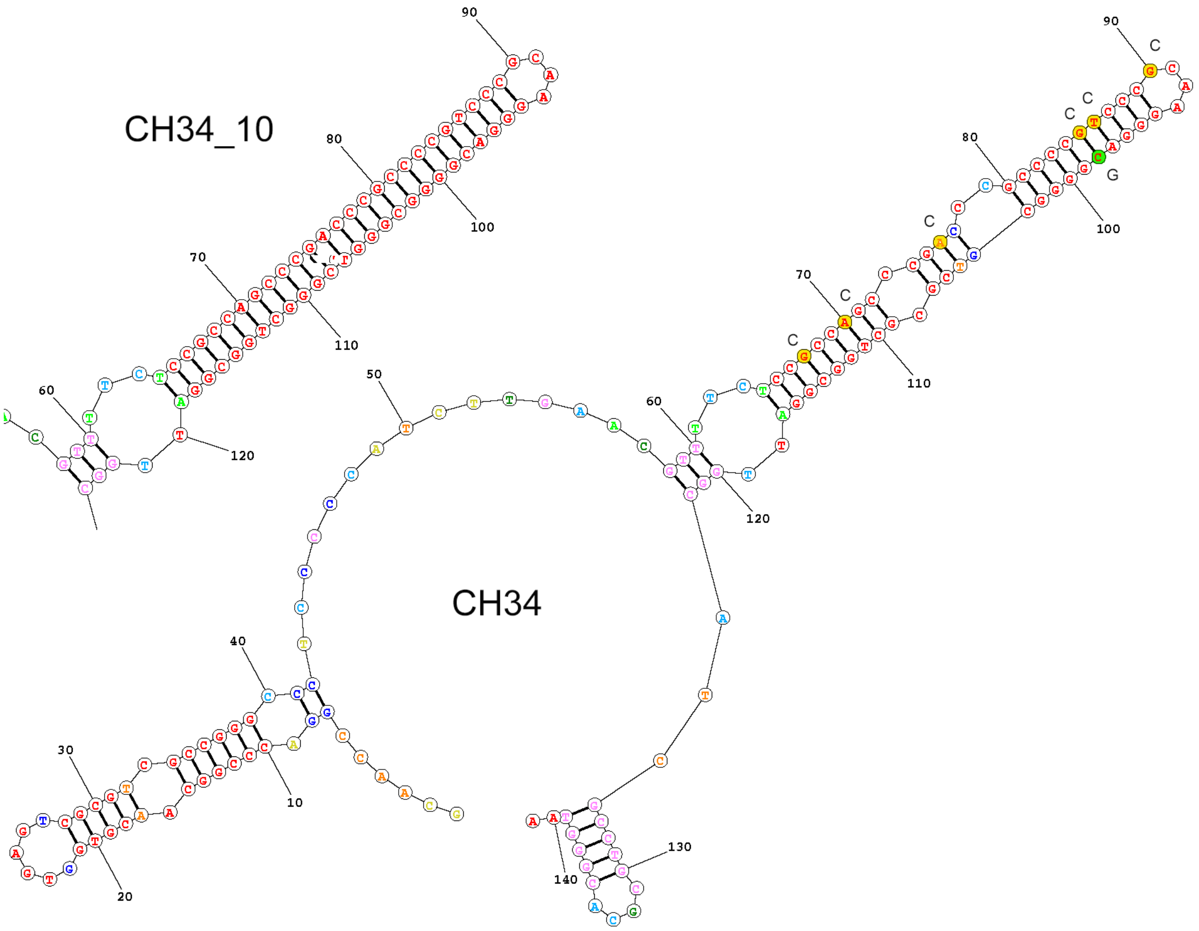

Supplementary Figure S5. Predicted secondary structure of the intergenic region between *mreB* and *mreC* on the chromosome of C. metallidurans. This structure was predicted using https://rna.urmc.rochester.edu. The mutations found in CH34_10 lead to a change of the large stem-loop as indicated. The yellow labels show the nucleotides mutated in CH34_10 with the changed nucleotides beside. In one case, these mutations keep the base-pairing between a G and a C (yellow-green).

Careful analysis of the genomic sequence of strain AE104 revealed an emerging deletion A of the island CMGI-2 (Table 1, indicated by a small "a"; Suppl. Fig. S7A, left-hand) as indicated by low coverage of the island by the sequence reads. Even island CMGI-3 (Suppl. Fig. S7A, right hand) displayed many local regions of low coverage in CMGI-3. While both regions were completely deleted in strain AE104_10, a part of the sub-population of strain AE104 also carried deletion A and an even smaller proportion also deletion C.

To understand how the selection pressure for the plasmid-encoded metal resistance affected mutations and large deletions, strains AE126(pMOL28) and AE126_10 were considered. The latter strain was kept for more than 10 years on TMM but with alternating selection for the cobalt-nickel resistance determinant *cnr* using 1 mM Ni(II) and 275 µM chromate for *chr*. Although regularly revived from frozen stock, strain AE126 carried a nearly

complete deletion of region B as indicated by only very few annotated sequence reads (Table 1, indicated by a "(B)", Suppl. Fig. S7B, right hand), accumulation of regions of low coverage on region A (left hand) and an even lower coverage of region C (Fig. S7D). Compared to this, region B was completely gone in strain AE126_10 despite the selection pressure (Suppl. Fig. S7C, right hand). Regions A (S7C, left hand) and E displayed emerging deletions (Table 1).

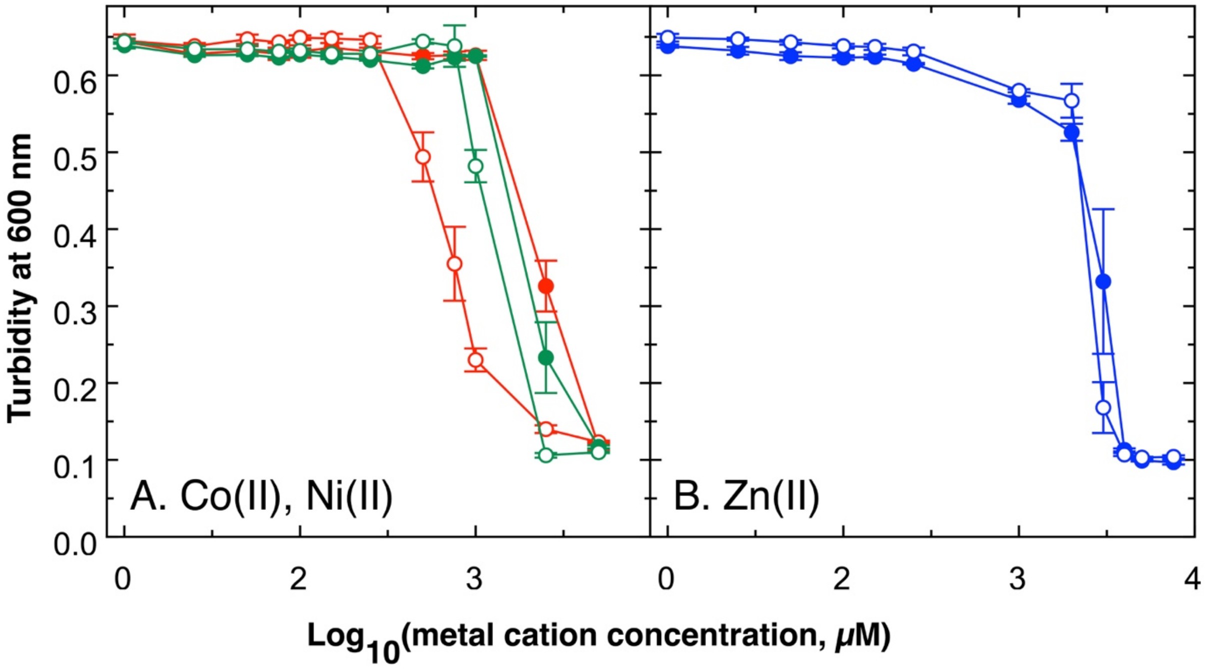

Supplementary Figures S6. Comparison of metal resistance of strains CH34 and CH34_10.
The dose-response experiments show the turbidity of CH34 (closed symbols) and CH34_10 (open symbols) in the presence of increasing concentrations of Co(II) (Panel A, red), Ni(II) (Panel A, green) or Zn(II) (Panel B, blue) after 20 h of growth at 30°C in TMM with shaking. Three independent experiments were carried out and the standard deviations are indicated.

Only CH34 wild type was completely stable under the appropriate conditions. Deletions A, B, C, and E appeared when either a plasmid was deleted, selection pressure for the presence of the plasmids or revival from the frozen stock were omitted. When one or two of these factors were present, loss of the regions was reduced but not prevented. Deletions started with regions of low coverage. This means that in the pure cultures an increasing percentage of a subpopulation carried the deletions.

No mutations were found in strains AE126 and AE126_10 on the chromosome, chromid of plasmid pMOL28 (Table 1, Supplement). Strain AE104 carried 2 mutations, transversions leading to substitutions in two predicted FAD-dependent oxidoreductases involved in lipid metabolism encoded in the chromosome and chromid, respectively, with the chromid gene not expressed. Strain AE104_10 contained two additional mutations on the chromosome, a transversion leading to truncation of a gene for a putative amidase and a transition leading to a substitution in a not expressed gene encoding a putative uncharacterized protein.

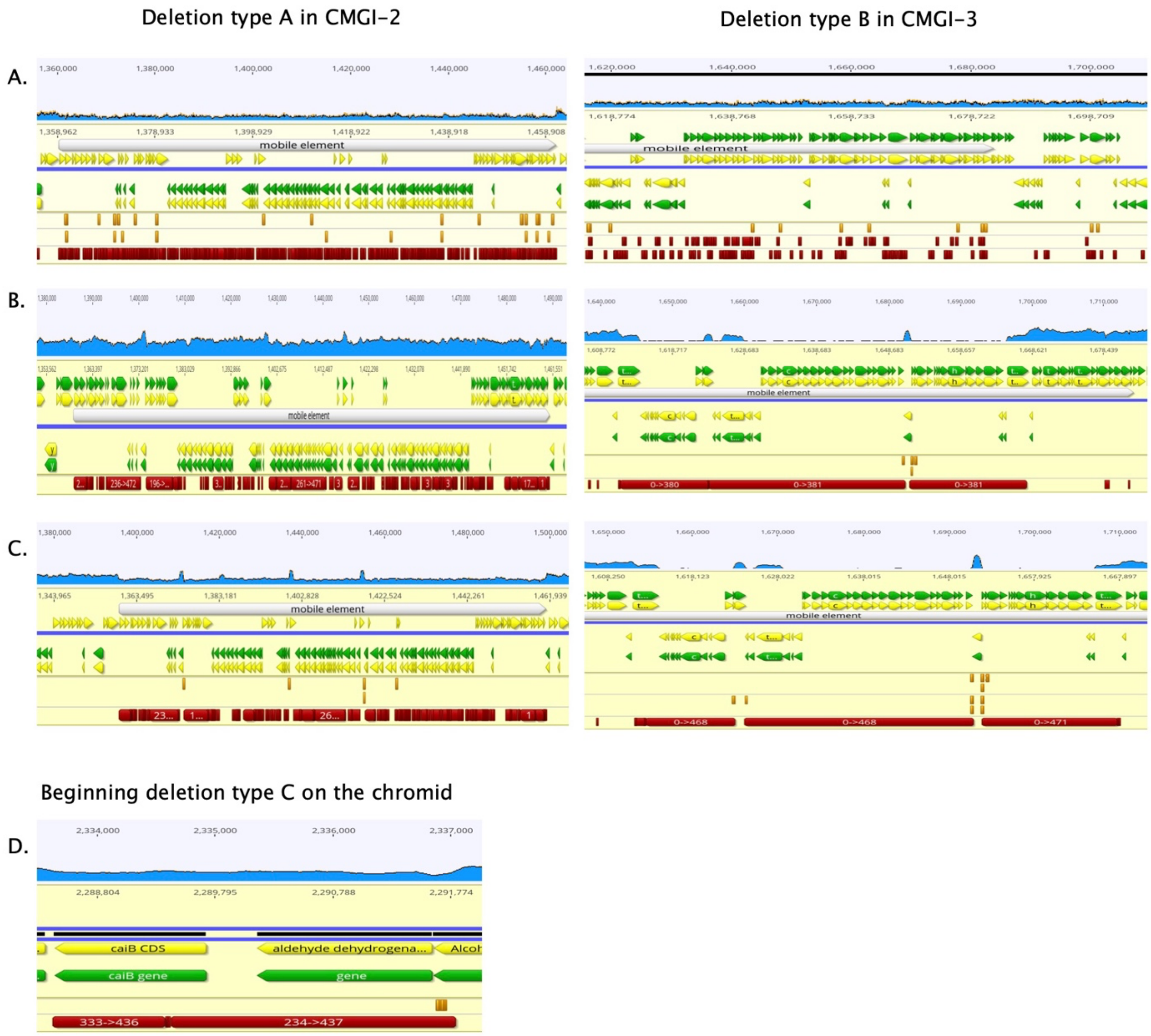

Supplementary Figure S7. Emerging, nearly complete and complete deletions type A, B and C in strains AE104, AE126 and AE126_10. The screenshots show the regions A and B in strain AE104 (Panel A), AE126 (Panel B), AE126_10 (Panel C) and the region C in strain AE126 (Panel D). The sequence coverage is shown in blue, the open reading frames in yellow and green, regions of low coverage in brown. The positions on the chromosome and chromid, respectively, are on the top. The figure shows: (i) a very early state of the deletion as indicated by a large number of poorly covered single nucleotides in region B of strain AE104; (ii) a mid-level of deletion abundance indicated by the existence of reads, but large continuing regions in region A in all strains and region C in AE126; (iii) a nearly complete deletion with very few reads in region B in AE126; and (iv) a complete deletion of region B of strain AE126_10.

A mutant strain of AE104 named AE104_5 had been identified by chance and had lost its resistance to cadmium and cobalt when compared to its parent (Suppl. Fig. S8). Cadmium and cobalt resistance of strain AE104_5 was similar in TMM containing 1 mM Mg(II) compared to the parent strain AE104 in TMM with only 100 µM Mg(II) (Suppl. Fig. S8). Since a low magnesium content increased metal cation uptake (7), it was expected that strain AE104_5 may carry mutations in regulators of magnesium uptake, for instance the *pho* regulon or riboswitches upstream of the genes encoding the magnesium-importing P-type ATPases *mgtA* or *mgtB*. AE104_5 and as an additional control its parent (designated in the sequencing

experiment "AE104_4") were both sequenced. As expected, AE104_4 carried the same mutations and deletion as AE104 (Table 1). In its cadmium-sensitive descendant AE104_5, deletions A and E were complete, while in region B, the genes encoding the Calvin cycle enzymes and the soluble hydrogenase were still present. Three possible mutations were found. One was a transversion in an intergenic region between two promoters for a putative uncharacterized protein Rmet_4729 and two leading to two different substitutions in the gene *Rmet_3488* for a periplasmic substrate-binding protein of an ABC import system for branched-chain amino acids. The mutation changed a 50-AspMet-51 sequence to a GlyGly. This mutation was outside of the predicted substrate-binding site of this protein. Since a connection to genes or intergenic regions involved in magnesium uptake was not evident in the mutations carried by strain AE104_5, decreased resistance to cadmium and cobalt may be connected with the large deletions rather than to possible mutations. Interestingly, only one other mutant carried an A deletion but kept region B, a double mutant in *zur* and *cobW2* (Table 1). Only very few regions of low coverage were found in region B in these two strains (Suppl. Fig. S9).

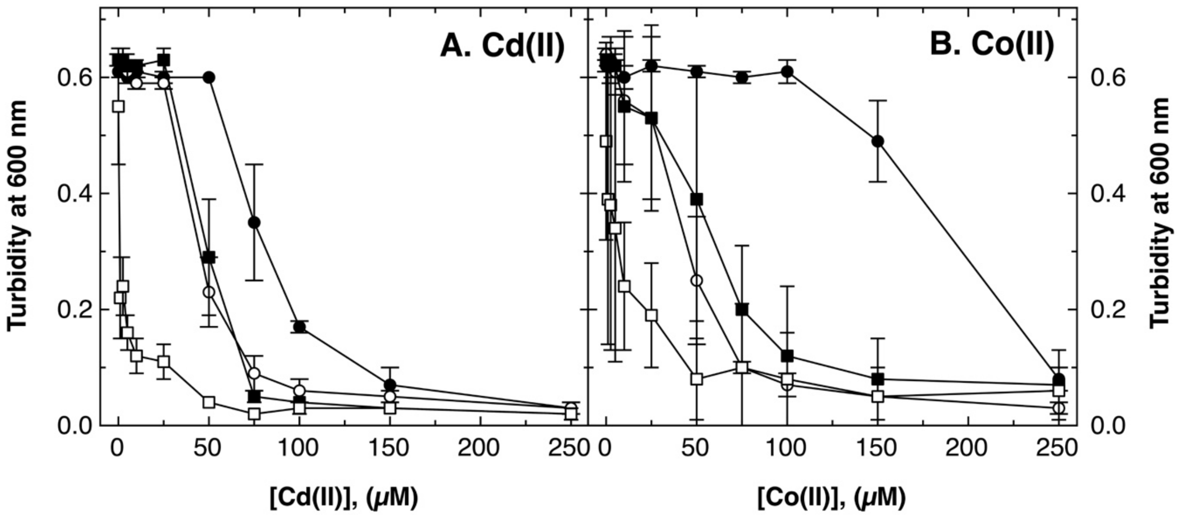

Supplementary Figure. S8. Metal resistance of a spontaneous mutant of C. metallidurans AE104. Dose-response curves show the resistance of a spontaneously evolved mutant AE104_5 (open symbols) compared to AE104 (closed symbols) in the presence of increasing concentration of Cd(II) (Panel A) or Co(II) (Panel B) in Tris-buffered mineral salts medium TMM (circles) or TMM with 100 µM Mg(II) instead of 1 mM (squares). Deviations shown, n ≥ 3.

When not prevented by usage of frozen stock and selections pressure to maintain the two plasmids, *C. metallidurans* accumulated only few mutations but lost predominantly the regions A (CMGI-2, membrane-bound hydrogenase), B (CMGI-3, Calvin cycle and soluble hydrogenase), C (*caiB*-region on the chromid) and E (CMGI-4 except Tn*6048*; *hmz* determinant, gene for MetR, a methylene-THF and a ferric reductase that may be linked to metal homeostasis). Selection pressure that allows to keep region B despite a type A deletion may be connected to a cadmium and cobalt sensitive phenotype of a spontaneous AE104

mutant. This indicated that the four regions A, B, C and E may be involved in metal homeostasis, but only when both plasmids were present in the cells of *C. metallidurans* and selection pressure was continuously applied.

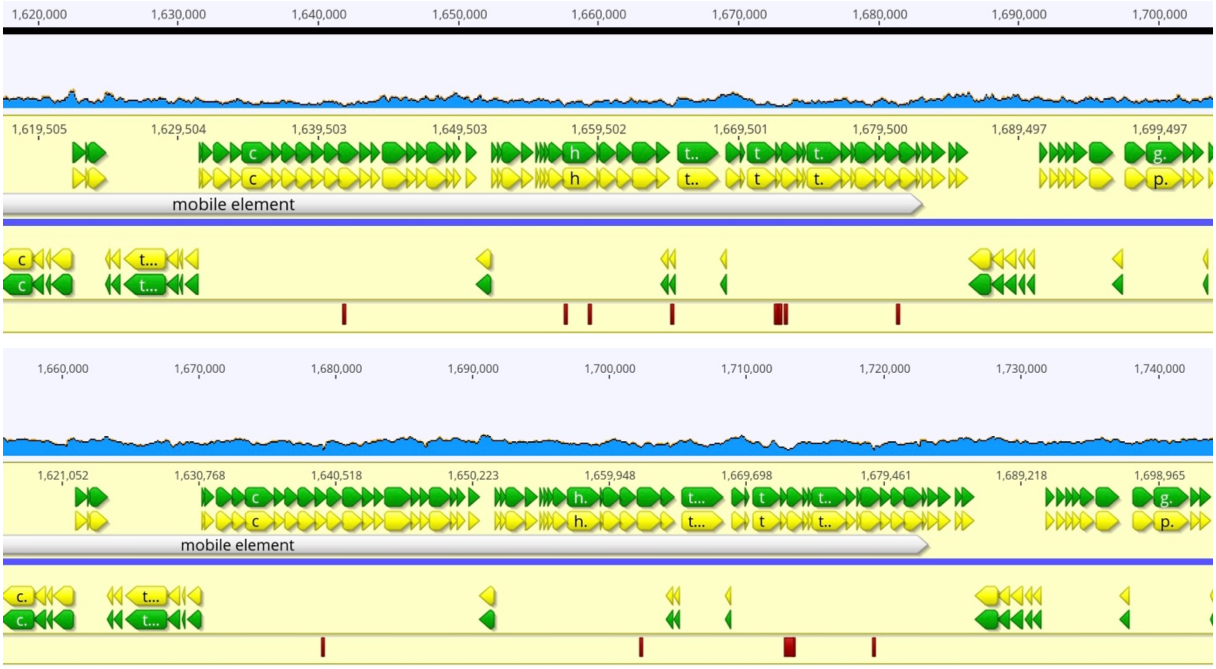

Supplementary Figure S9. No trace for a beginning deletion type B in two mutant strains of AE104. The screenshot shows the region B in strain in the cadmium sensitive strain AE104_5 (top) and the AE104 mutant Δ*zur* Δ*cobW2* (bottom). The sequence coverage is shown in blue, the open reading frames in yellow and green, regions of low coverage in brown. The position of the mutant genomes on the chromosome are on the top. The figure shows only very few regions of low coverage (brown bars) in these two strains.

<u>Sigma factors.</u> The four mutants with deletions in genes for extracytoplasmic function sigma (ECF) factors, which all contained both plasmids, had different patterns of large deletions (3, 8). None had a type E deletion. This appeared only in plasmid-free strains except the AE104 parent, the Δ*zur* mutants and as an emerging deletion in strain AE126_10 (Table 1). Selection for the presence of the plasmids or deletion for Zur prevented deletion E. As published, strains DN554 (Δ*rpoO* Δ*rpoL* Δ*rpoM::pLO2*) and DN546 (Δ*rpoQ* Δ*rpoR*) had type A, B and C deletions (Table 1), whereas strains DN555 and DN553 carried complementary deletions. DN555 (Δ*rpoE* Δ*rpoP* Δ*cnrH::pLO2*) with deletion of the sigma factor CnrH for high-level expression of the nickel-cobalt resistance operon *cnr* on plasmid pMOL28 kept both regions A and B for the hydrogenase islands. Strain DN553 (Δ*rpoI* Δ*rpoJ* Δ*rpoK*) lost these but kept region C. The three sigma factors absent in strain DN553 were needed to mollify the effect of the “iron-first” rule of *C. metallidurans* on the homeostasis of other transition metals (8). This connected the genomic island encoding the nickel-depending hydrogenases to nickel resistance on plasmid pMOL28

and the *caiB* region C to the interaction of iron homeostasis with that of other transition metal cations (3).

Strain DN555, which exhibited a higher cellular nickel content (8) and kept both hydrogenase gene clusters, had just one additional mutation, a deletion of an 11 bp in a repeat region that decreases the number of repeats from four to three. This deletion occurred in a 5' untranslated part adjacent to Rmet_3818 on the chromid and was not in the vicinity of a TSS. Most likely, this deletion had no phenotypical effect so that the type C deletion was the only unintended mutation in this strain.

The other three sigma factor mutants DN554, DN553 and DN546 shared two changes in addition to the loss of the two hydrogenase islands. Both mutations were also present in strain DN832, a triple Δ*cobW* deletion mutant of strain CH34 wild type, which also possessed both plasmids. One was a silent transition in Rmet_1484, which is not expressed and encodes a putative uncharacterized protein. The second change was a T->G transversion resulted in a F->V exchange in position 277 of the expressed gene *bzdA* (Rmet_1224) encoding a benzoate-CoA ligase. Strain DN554 additionally suffered from a silent mutation in Rmet_1596 for the DNA translocase FtsK.

One of the four unique changes in strain DN546(Δ*rpoQ* Δ*rpoR*) was deletion of an A from a $(A)_9$ repeat outside of a gene and not in the vicinity of a transcriptional start site. Two mutations on the chromosome affected two neighboring genes. One transition led to a Y->H exchange at position 217 of the product of Rmet_2439. In the open reading frame of Rmet_2440, 12 nucleotides were deleted in a repeat region of 5 copies so that 4 copies remained. This *in frame* deletion removed at base position 2821 the codons for the four amino acids PVAA from a $(PVAA)_5$ repeat in the primary sequence. Both genes are expressed from a common promoter region but are located on different DNA strands. Rmet_2439 in the reverse direction encodes the pseudiuridine synthase RluC and Rmet_2440 in the forward direction the RNAse E. Both gene products had a role in translation, RNA modification and stability.

The last unique mutation in strain DN546 concerned the *copA1* gene on plasmid pMOL30, which is involved in high-level copper resistance (9). It encodes a periplasmic copper-dependent copper oxidase related to CueO from *E. coli* (10-14). These proteins belong to the multicopper oxidases that oxidize periplasmic Cu(I) to Cu(II), which subsequently decreases import of Cu(I) ions from the periplasm to the cytoplasm (15). The mutation in *copA1* of strain DN546 led to an *in frame*-deletion of an MGG in a region containing 18 of the total 36 Met residues of the protein, which also organizes a part of the protein (Suppl. Fig. S10). Methionine-rich loops serve as sponges that scavenge copper ions in the vicinity of the substrate-binding site (16). This may decrease the number of Cu(I) bound to the copper sponge by one.

Strain DN546(Δ*rpoQ* Δ*rpoR*) with a perturbed thiol homeostasis may have compensated a part of this effect by a mutation in CopA1 and two factors involved in translation. The other changes in sigma factor mutants were not conclusive. None of these mutants had polymorphisms in the genes for other sigma factors.

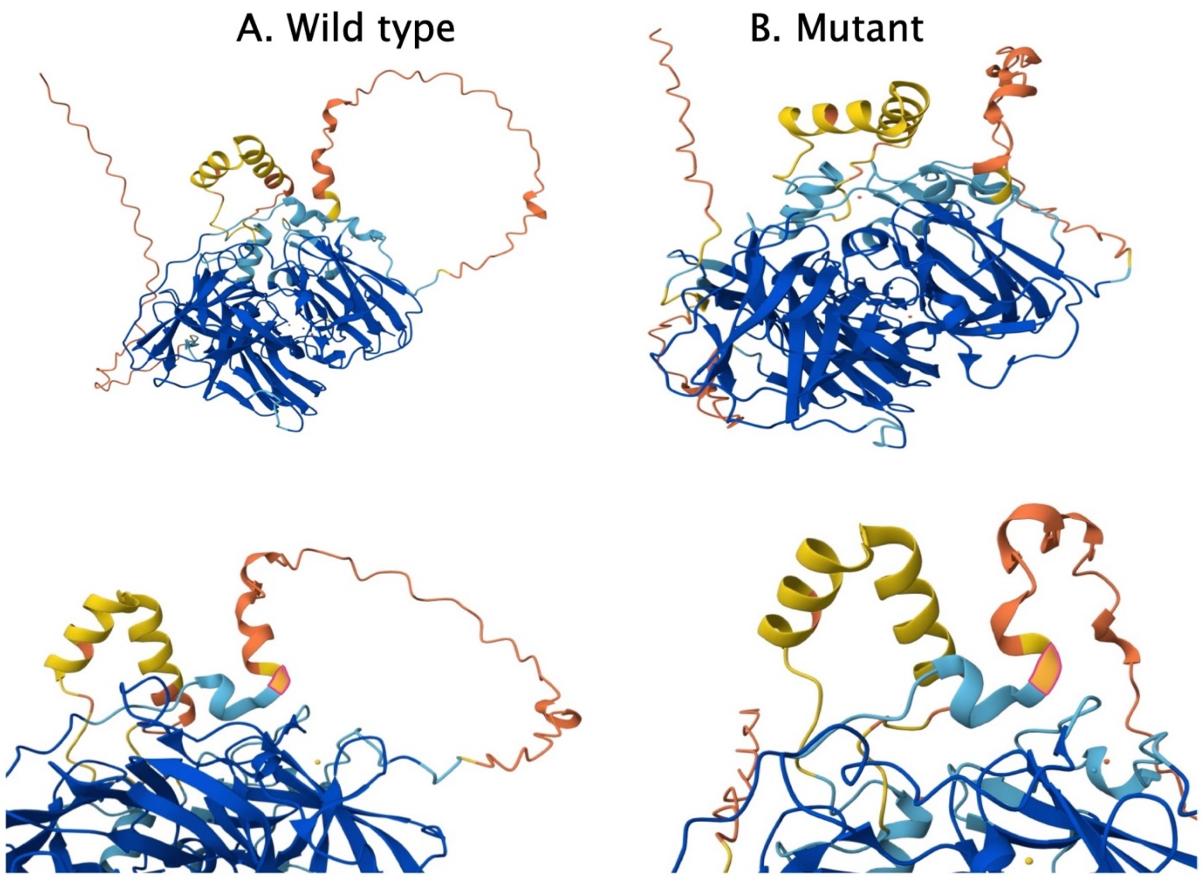

Supplementary Figure S10. CopA1 wild type (Panel A) and mutant strain DN546(Panel B). Modeled with AlphaFold3 (17). Below each model, a close-up of the mutated region is shown. The program simulates a more ordered structure in the mutant compared to the wild type.

<u>Uptake systems.</u> All the strains with deletions in the genes for uptake systems carried the type A, B, C and E deletion that were also present in strain AE104_10, sometimes as nearly complete or emerging deletions (Table 1). The Δ7 strain (Δ*zupT* Δ*corA1* Δ*corA2* Δ*corA3* Δ*pitA* Δ*hoxN* Δ*zntB*) (18, 19) had additionally deleted region F encoding a second carnitine dehydratase, in addition to *ciaB*. Removal of the two genes for magnesium-transporting P-type ATPases, MgtA and MgtB, in strain Δ9 resulted in three more deletions in regions G, H and I. Deletions G and H were unique for strain Δ9, leading to loss of a hypothetical exported protein with an un-interrupted stretch of 50 Leu residues in G and two putative uncharacterized proteins in H. Deletion I had also been observed in CH34_10. Both mutants lost a gene for the DNA-binding protein H-NS, which silences foreign genes. Removal of the genes for uptake systems not only led to a loss of fitness (18, 19), but also to decreased stability of the genome.

Deletion of the Δ*zupT* gene for the main zinc importer in *C. metallidurans* caused severe pleiotropic effects, for instance problems to fold the beta-prime subunit RpoC of the RNA polymerase, although the cells were still able to accumulate sufficient amount of zinc (7, 20). Two Δ*zupT* deletion strains that were kept for different periods of time on TMM, differed with respect to the deletion types. All had deletions A, B, C and E like AE104_10, although only as an emerging deletion ("b" in Table 1) in two strains. The presence of a copy of the *czcCBAD'* region cloned on the vector pVDZ'2 (21) leading to plasmid pDNA130 (22) changed neither the pattern of large deletions, nor did it yield unique mutations (Table 1).

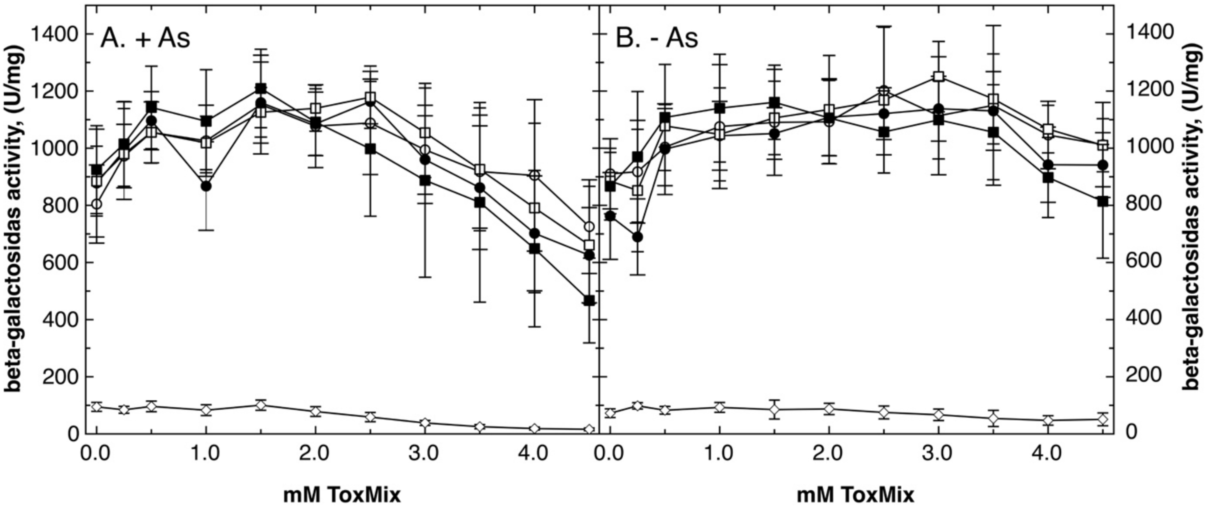

Supplementary Figure S11. Comparison of the activity of the parent and mutated *rpoH* promoter. The *rpoH* promoter fragments from parent strain AE104 (circles) and its Δ*zupT* mutant (squares) were cloned upstream of a promoterless *lacZ* gene on plasmid pVDZ'2. The resulting plasmids were conjugated into strains AE104 (open symbols) and Δ*zupT* (closed symbols). Negative control was strain AE104 with the promoter-less *lacZ* on plasmid pVDZ'2 (open diamonds, ◇). The strains AE104 with the original *rpoH* promoter (open circles, ❍), with the mutated promoter (open squares, ☐), strain Δ*zupT* with the original promoter (closed circles, ●), the mutated promoter (closed squares, ■) or the control (diamonds, ◇) were cultivated to the exponential phase of growth, AE104-specific ToxMix with (Panel A) or without (Panel B) arsenate was added, the cells were incubated with shaking at 30°C for 3 h and the specific activity of the beta-galactosidase reporter was determined. Three independent biological experiments were performed and data are presented with standard deviation.

All Δ*zupT* strains showed the same four mutations. A transversion on the chromid would result in an L->Q exchange in Rmet_4937 for a transcriptional regulator of the AsnC family. This gene was not expressed in strain CH34 under standard growth conditions. Two possible mutations on the chromosome and chromid, respectively, affected the genes for an uncharacterized protein and for a lysine exporter, both at a low-expression level. The second mutation on the chromosome was located upstream in the promoter region of the heat-shock sigma factor, RpoH. The *rpoH* gene contained two transcriptional start sites (23) and the T->C transition occurred at position -31 with respect to one of these promoters, which was 4 base pairs downstream of the -35 region in the space between -35 and -10 site. The mutated region

upstream of *rpoH* in strain DN515 (Δ*zupT*) and the same region in the parent strain AE104 were cloned upstream of a promoterless *lacZ* gene on vector pVDZ'2 (21) and transferred into strains AE104 and DN515. The original and the mutated *rpoH* regions clearly resulted in expression of the reporter gene in both strains (Suppl. Fig. S11). The strains were incubated in the presence of a mixture of toxic metal with or without arsenate. Expression of the reporter in the presence of increasing concentration of a mixture of toxic metal ions did not result in differences between the promoters or strains (Suppl. Fig. S11) but demonstrated that the mixture with arsenate was more toxic than that without the metalloid oxyanion. The mutation upstream of *rpoH* had no effect under the tested conditions.

A mutant strain with a deletion Δ*feoB* of a central iron instead of the zinc uptake system suffered from the same large deletions A, B, C and E, albeit with a few sequence reads still annotated to region B (Table 1). As in strain AE126, this region was deleted in most, but not all, cells. Two additional mutations were found in the chromosome of the Δ*feoB* strain. One was a transition leading to a substitution in the gene encoding a C4-dicarboxylate transport system that was not expressed in strain CH34 under standard conditions. The second mutation was a deletion of one repeat out of four in a $(GCC)_4$ motif in the gene for the magnesium uptake system *corA1* leading to deletion of an alanine residue from an AAAAD sequence. Comparison of the modeled CorA1 protomers from mutant strain and parent with the crystal structure of CorA from *Thermotoga maritima* (17, 24)(Fig. 2) indicates that the mutation may change the gating mechanism, substrate selectivity or flux control of this protein. Removal of the central Fe(II) import system of *C. metallidurans* led to a mutation that might be a suppression, which adapts homeostasis of Mg(II), Co(II) and other divalent metal cations to a barred import route for Fe(II).

Deletion of more genes for possible zinc uptake systems in strain Δ*zupT* (Fig. 1) led to the Δ7 strain (Δ*zupT* Δ*corA1* Δ*corA2* Δ*corA3* Δ*pitA* Δ*hoxN* Δ*zntB*) (18, 19). This strain accumulated less zinc compared to the Δ*zupT* strain, showed a decrease in fitness but was still able to survive at low zinc concentrations in the growth medium (7).

In addition to the type A, B, C, and E deletions that were already present in the Δ*zupT* strain, another deletion occurred in the Δ7 strain. This type F deletion was just 7 genes upstream of the type C deletion on the chromid and affected two silent genes (NPKM ≤ 10. nucleotide activities per kilobase of exon model per million mapped reads), one a small open reading frame, the other a possible L-carnitine dehydratase. Three mutations plus 4 possible ones appeared in addition to those in the Δ*zupT* strain. A silent transition on the chromosome concerned Rmet_2247 for a diguanylate cyclase. A deletion of one unit of a fivefold repeat of 8 bps occurred 26 bp downstream of the gene encoding the nickel chaperone and GTPase UreG on one DNA strand and also 44 bp downstream of the gene for a regulatory protein on the other. A possible deletion (88.4%) deleted one C from a nine-C stretch 347 bp downstream

of the gene for porin Rmet_3234 within the transcribed 3UTR of this gene. A transition led to an Ala->Val substitution in Rmet_5938, which encodes a membrane protein with two domains. The N-terminal 200 amino acids are an integral membrane protein related to the DedA protein YghB from *E. coli*, which seemed to be a membrane transporter involved in drug resistance (25). The carboxy-terminal domain showed signatures of the rhodanese superfamily including the conserved Cys residue (26). Rhodaneses are sulfur transferases and widespread in bacteria. The A->V exchange was located at position 120 at the beginning of the third transmembrane alpha-helix, which increased the hydrophobicity of this transmembrane helix. If any effect would be visible, the mutation should facilitate synthesis of this protein and its insertion into the cytoplasmic membrane. Finally, three possible mutations occurred in Rmet_1656 or *yhjG* encoding a glutathione-S-transferase-like protein, two deletions and a transition. These three possible mutations are just part of a variety of polymorphisms in between codons 215 and 244 of this gene, which were mostly below the 50% threshold of polymorphisms that were judged as possible or almost certain mutations. These mutations would cause frame-shifts and disrupt the gene, which is on a low expression level in strain CH34 wild type. The Δ7 mutant might possess suppressions that change regulatory processes connected to porin production and sulfur metabolism.

Strain Δ9 resulted from a deletion of the gene for the Mg/Ca-transporting P-type ATPase MgtB and an interruption of its paralog *mgtA*. No marker-free deletion of *mgtA* could be obtained in the Δ7 Δ*mgtB* or the Δ7 mutant (18, 19). In pulse-chase experiments, Δ9 displays a decreased accumulation of zinc ions compared to Δ*zupT* and on a similar level as the Δ7 strain under metal-starvation conditions. Interestingly, when sufficient zinc was present, Δ9 accumulated zinc with a higher initial uptake rate than the Δ*zupT* strain, Δ7 or even the parent AE104 (7). A zinc uptake system may have been activated in the Δ9 strain although no transcriptome investigations identified this system (18, 19).

Compared to the Δ7 strain, the Δ9 strain possessed three additional large deletions and also three additional SNPs. The type G deletion was located at the 5’ end of Rmet_6477, that was not initially annotated (1). Rmet_6477 is located in the same region as Rmet_1797 on the other strand. This non-expressed gene encodes a putative uncharacterized protein. The type G deletion was located at the non-overlapping parts between both open reading frames. The type H deletion removes the two expressed open reading frames Rmet_2161 and Rmet_2162 on the chromosome (Table 1). Rmet_2162 contains at its N-terminus the domain of unknown function DUF4365 (pfam14280). Rmet_2161 belongs to the Piwi-like superfamily, which occurs in bacteria, archaea and eukaryotes. PiWi is an Argonaute subfamily protein involved in RNA interference (27).

The type I deletion occurred again on the chromid, just 18 genes downstream from the type C deletion (Δ*Rmet_5561_6747_5562_5563*). These genes are all expressed in *C.*

metallidurans from a common promoter region with *Rmet_5562_6747_5561* in the backward and *Rmet_5563* in the forward direction. Rmet_5561 was annotated as a transcriptional regulator of the XRE family and Rmet_5562 as H-NS-type DNA-binding protein. The other two locus tags encode putative uncharacterized proteins. H-NS proteins compact the nucleoid (28) and interfere with gene expression (29), silencing foreign DNA elements (30-33). The deletions appearing in the Δ9 strain removed a gene encoding a regulator, a H-NS protein that may silence foreign DNA and a protein involved in RNA interference. It is tempting the speculate that these deletions may be connected to the appearance of the zinc-import activity in the Δ9 strain by un-blocking expression of a metal importer gene.

The additional three mutations were all located on the chromid, a transition in Rmet_3908 leading in a I->T exchange, an insertion into a tandem repeat region resulting in insertion of additional GH amino acids into Rmet_4198 and a silent transition into Rmet_4771. *Rmet_3908* is expressed on a low level and encodes an MscS-type mechanosensitive ion channel, *Rmet_4198* a transcriptional regulator of the PadR-family and *Rmet_4771* another regulator of the RpiR family. The I->T change at position 117 in *mscS* is located in the first predicted extracellular part of the protein between the transmembrane alpha helix 1 and 2 of this 771 aa protein with 12 transmembrane spans. As modelled by AlphaFold 3, this change mediates an approach of two periplasmic alpha-helices, which may form a zinc-binding site formed by three His and an Asp or Glu residue (Fig. 3).

In the Δ9 strain compared to Δ7, deletion or interruption of the two magnesium-importing P-type ATPases led to a loss of factors involved in regulatory tasks, gene-silencing, RNA-interference and regulation of transcription initiation. These deletions might have been required to produce the transport activity measured in the pulse-chase experiments with Δ9 cells cultivated in the presence of 1 mM magnesium and 200 nM Zn(II), but not 0.1 mM Mg(II) and zinc-starvation conditions (7). The target of these regulatory processes might be the MscS-type mechanosensitive ion channel Rmet_4198.

<u>Efflux systems.</u> Deletion of the four efflux systems known to be involved in Zn(II) export from the cytoplasm to the periplasm (34, 35), namely the two P-type ATPases ZntA and CadA, as well as the two CDF proteins DmeF and FieF (36, 37), led to the Δe4 strain. This strain and its descendants carried the A, B, C, and E deletions, plus two additional ones, J and K (3). Type J included the deletion on the chromosome of the gene encoding a small hypothetical protein Rmet_6403 and a fragment of a TonB-dependent outer membrane protein Rmet_0032, which was already interrupted by a transposon insertion. The type K deletion on the chromid comprised the region Rmet_4603 to Rmet_4618. This deletion was located eight genes away from the intended deletion of *zntA*. The *zntA* gene was part of an ancient *zntA*<->$czcI_2C_2B_2A_2$ region on the chromid, which was interrupted by insertion of a Tn*6050* transposon into $czcB_2$

and subsequently to a separation of *zntA<->czcI*$_2$*C*$_2$*B'*$_2$ and *czcB"*$_2$*A*$_2$ (38, 39). The type K deletion occurred directly downstream of the Tn*6050* transposon and included two genes encoding TonB-dependent outer membrane proteins, an enzyme involved in methionine biosynthesis, a histidine protein kinase and a periplasmic substrate-binding protein. This deletion might decrease the import of metal cations into the cell.

Additionally, strain ∆e4 carried 7 mutations, all within genes. A transversion on the chromosome in *Rmet_0730* for an uracil-xanthine-permease was silent. An *in-frame* insertion of 3 amino acids into *Rmet_1145* was within a non-expressed gene encoding a predicted TorA-like molybdenum cofactor-containing reductase. The third chromosomal mutation resulted in a G->A transition at codon position 70 of gene *Rmet_3077* for a weakly expressed (NPKM = 15±1) TonB-dependent outer membrane protein. Three mutations on the chromid included the *in-frame* insertion of two additional amino acid into the non-expressed *Rmet_3834* for a L-carnithin dehydratase, a deletion of two 9 bp repeats out of six yielding an *in-frame* deletion of the six amino acids PTPPTP from a much longer PTP-repeat of a TetR-family regulator Rmet_4723 and another *in frame* deletion of 6 base pairs of the two amino acids "DH" in *Rmet_4943* for an iron-containing alcohol-dehydrogenase.

TetR-family regulators contain the DNA-binding helix-turn-helix motif at the N-terminus (40). Rmet_3834 is located in a "type III" setting (40) in an operon with the gene for a cardiolipin synthetase upstream and for a putative lipoprotein downstream, such that a role in lipid metabolism is likely, as in case of many other TetR-family members (40). The deletion that decreases the number of PTP repeats in Rmet_4723 is in a disordered N-terminal extension upstream of the DNA-binding helix-turn-helix (Suppl. Fig. S12). There is no indication for another start codon located more closely to the helix-turn-helix-encoding part. Other TerR-family members such as SimR also contain a N-terminal extension that interacts with the minor groove of the DNA to enhance DNA binding (41). The deletion in Rmet_4723 would decrease the number of Thr residues interacting with the minor groove from 6 to 4, which should have only a mild effect or no phenotype. Overall, mutation in the gene encoding this regulator and the effect on two porins were the major changes in the ∆e4 mutant.

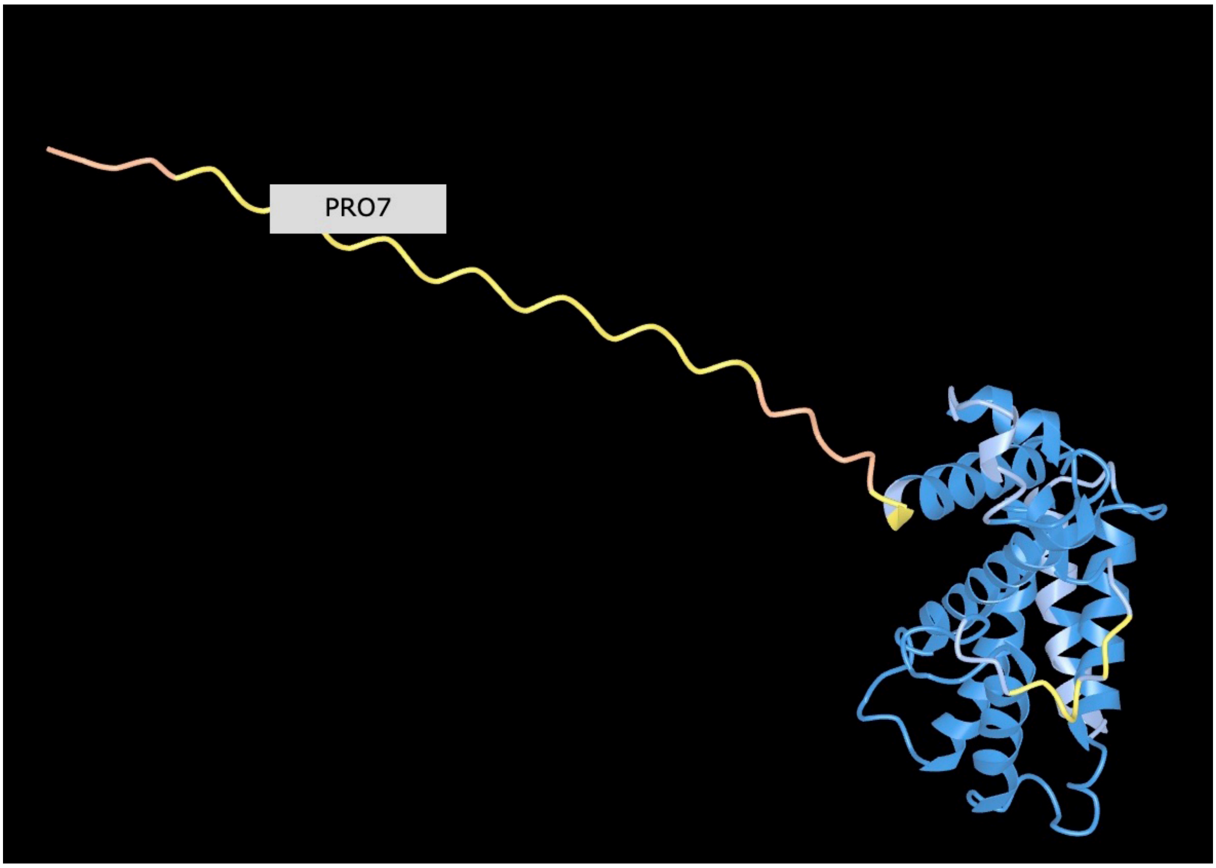

Supplementary Figure S12. Model of the TetR-like regulatory protein Rmet_4723. Modeled with AlphaFold3 (17).The position of the proline-rich region in the unstructured amino terminus is indicated by highlighting Pro7.

Deletion of the gene encoding the zinc importer ZupT in the ∆e4 strain resulted in two mutations in intergenic regions and 6 possible mutations, 5 of them in the gene *Rmet_3071*. Operon region Op0320 encodes the genes *hpcH* and *lysA* for enzymes involved in amino acid biosynthesis. A deletion of one G from a GGGG-repeat occurred on the chromosome in the 3UTR region of *Rmet_1110/lysA* and 123 bp upstream of another gene *Rmet_1109* for a putative uncharacterized protein, co-transcribed with the other two genes. The second mutation in an intergenic region was a transition 76 bp upstream of the transcriptional start site of *Rmet_4908* or *mmsQ* that encodes a further putative uncharacterized protein. This transition was probably outside of the promoter region for this gene. A possible mutation was an insertion causing a frame-shift mutation of the weakly expressed *Rmet_2171*, again encoding a putative uncharacterized protein. The five possible mutations were in the highly expressed *Rmet_3071* for a histone H1-like DNA-packaging protein. Reminiscent to *Rmet_1656*/*yhjG* in the ∆7 uptake mutant, the five possible mutations were surrounded by a variety of polymorphisms below the threshold level of a frequency < 50%, located between codon numbers 113 and 160, which should inactivate this gene. Deletion of *zupT* in the quadruple efflux mutant ∆e4 might lead to a suppression that inactivates a DNA-packing protein.

The *cdfX* gene encodes a CDF protein functioning as additional zinc efflux system that relieves the main zinc-cadmium exporting P-type ATPase ZntA under certain conditions (42). Deletion of *cdfX* in Δe4 results in the quintuple efflux mutant Δe5, accompanied by four mutations and a possible one, all within genes. Three of these mutations led to substitutions in putative uncharacterized proteins and an N-acetyl transferase, plus a deletion leading to a frame-shift mutation in the gene for a predicted transcriptional regulator. The remaining mutation, a deletion of a Phe from a FFFF sequence of YidC, was described and discussed in the main text.

The Δe5 strain was still able to efflux zinc, indicating the presence of yet another transport protein with metal efflux activity in *C. metallidurans* (42). A candidate could be the ABC transporter AtmA that was involved in nickel and cobalt resistance (43). The Δ*atmA* gene was deleted in the Δe5 strain leading to Δe6 as well as in the AE104 Δ*cdfX* strain. Zinc and cadmium resistance of AE104 Δ*cdfX* was not affected by the additional Δ*atmA* deletion (Fig. 5). While zinc resistance of Δe6 compared to that of Δe5 seemed to be lower, albeit not significantly, there was no difference in cadmium resistance.

Strain Δe6 had acquired one mutation on the chromid plus two possible deletions on the chromosome. On the chromid, the gene encoding a fusaric acid-resistance protein had a transversion leading to an Asp->Ala substitution. The respective gene was expressed at a low level. The two possible mutations on the chromosome affected the gene for the important PitA import system Rmet_1973 of metal phosphate complexes. Both were transitions leading to an Ile -> Val and a Val -> Ala substitution. Reminiscent to two other examples mentioned above, these two possible mutations were surrounded by a variety of additional polymorphisms in the region of codon 215 to 236 (Fig. 6). The VI->AV mutations with a variant frequency > 50% were connected either to low-frequency polymorphisms upstream (Fig. 6, GH, blue) or downstream (red), which may affect the formation, stability or membrane insertion of the transmembrane helices 6 to 7 (Fig. 6). Loss of all known efflux systems for divalent transition metal cations in the *C. metallidurans* Δe6 strain results in genomic sequence reads indicating variants in the Δe6 population that contain mutated *pitA* genes encoding an important metal phosphate uptake system.

Zur regulon components. The *zupT* gene, three genes encoding zinc-binding CobW-GTPases of the COG0523 protein family and some genes for zinc-salvage proteins are under control of the Zur regulator (44-52). CobW1 and the salvage proteins are only produced under severe zinc-starvation conditions. CobW2 is a zinc-storage protein that occurs in two different conformations. CobW3 has no GTPase activity but seems to control the activity of transport systems (7, 49-51).

Deletion of *cobW* genes was accompanied by deletions in the chromid that were not observed in other mutant strains (Table 1). On the other hand, some deletions occurring in these other mutant strains were absent. All Δ*cobW* mutants with an intact *zur* gene possessed the type A, B, and C deletions plus the type E deletion in the two AE104 derivatives, so that these two strains resembled the AE104_1 strain with respect to the type A, B, C and E deletions. The two Δ*zur* mutants had type B and C deletions but CMGI-2 was present in these two strains (Table 1).

New types of the deletions were type L in the CH34 derivative, M and N in the AE104 Δ*cobW* derivatives and type O in the Δ*zur* Δ*cobW2::*pLO2 strain, all in the chromid. The type N deletion Δ*Rmet_5689-5754* affected genes that were not expressed, or on a low expression level. In the type L deletion, Δ*Rmet_4208-4462,* with the exception of Tn*6049*, were lost, which was the region upstream of the $czcR_2S_2A_2B_2$” determinant. Deletion M Δ*Rmet_4434-4469* overlapped partially with L and comprised a deletion up to $czcB_2$”, or Rmet_4469. The genes downstream of Rmet_4469 were located on transposon Tn*6050*, which was responsible for the interruption of the ancient czc_2 determinant and the subsequent re-arrangement of the chromid. Deletion O Δ*Rmet_4603-4693* was downstream of another copy of Tn*6050*, located directly downstream of *zntA*<->$czcI_2C_2B_2$’. These deletions were reminiscent of the Tn*6050*-mediated interruption of and subsequent re-arrangement of the two halves of czc_2 after acquisition of the *czc* determinant on plasmid pMOL30 (39).

In addition to the deletion, strain CH34 (Δ*cobW1* Δ*cobW3* Δ*cobW2::pLO2*) contained two SNPs on the chromosome, one on the chromid but no SNPs on one of the two plasmids (Table 1). One chromosomal SNP was a transversion leading to a F->V substitution in Rmet_1224 or *bzdA*, which was expressed on a low level (NPKM = 20±2) and encoded a benzoate-CoA ligase needed for benzoate degradation. Since *C. metallidurans* was grown on gluconate as the carbon source, this transversion should not have a phenotype. The second SNP on the chromosome was a silent transition in Rmet_1484 for a putative uncharacterized protein. The respective gene was located directly upstream, with the *repA* gene of CMGI-3 on operon region Op0419f directly adjacent to the type B deletion in this strain. The SNP on the chromid increased the number of guanines in a G7-repeat to G8 in the 5’-UTR of *yebK* or Rmet_1044 encoding an RpiR-type regulator, which may be involved in regulation of expression of the *edd* transcript from a common promoter region from the other DNA strand. Edd is the 6-phosphogluconate dehydratase of the KDPG pathway, which is central to the usage of gluconate as carbon source in *C. metallidurans*. This mutation in the 5’-UTR may affect expression of *yebK*, for instance by changing the interaction of the *yebK*-mRNA with a small regulatory RNA.

Two SNPs occurred in the AE104 Δ*cobW3* Δ*cobW1*::pLO2 strain DN837 (genomic sequence 18). A chromosomal SNP was a substitution in Rmet_1748 leading to a F->L

exchange. Rmet_1748 encodes the AgrC outer membrane protein of the silver-inducible AgrCBA transenvelope efflux system (53), which is, however, expressed only on a very low level of NPKM = 6.3 ± 0.6 in non-challenged *C. metallidurans* cells. A chromid SNP was an *in-frame* deletion of 6 base pairs in a $(GTCGGA)_8$-repeat and subsequently of PT out of a $(PT)_8$ motif of Rmet_3966, which was expressed on a similar low level of NPKM = 5 ± 1. This is somewhat reminiscent of the deletion in Rmet_4723, and would decrease the number of Thr residues in the disordered N-terminal part of this protein.

Due to the low expression level of the two mutated genes, the SNPs in strain DN837 should not have a strong phenotype. Both mutations were also present in the sister strain AE104 Δ*cobW3* Δ*cobW2*::pLO2 DN838 (genomic sequence 19), indicating that they existed in the parent single mutant strain AE104 Δ*cobW3*, together with the deletions type A, B, C, E, M, and N. Strain DN838 had two additional SNPs compared to its sister DN837. An out-of-frame insertion of 8 base pairs into a tandem repeat region $(GAACGTCA)_7$ resulted in a truncation of the product of Rmet_0443 at CDS position 870, which truncated the gene product just before the stop codon. The gene encodes a putative uncharacterized protein. The other SNP also on the chromosome was an insertion of 3 bp (effectively a Gln residue added to Rmet_1033) expressed on a low level (NPKM = 13.7 ± 1.5) and encoding *slyB*. This SNP was also present in strain DN837, but with a lower variant frequency (Supp. Fig. S13).

In addition to the large type B and C deletions, the Δ*zur* mutant of parent AE104, strain DN728, possessed five SNPs, all on the chromosome (Table 1) and all in genes expressed in non-challenged cells with abundances between NPKM = 18 ± 1 and NPKM = 216 ± 9. Two SNPs were silent, a transversion in Rmet_0304 and a transition in Rmet_1762 encoding an ABC transporter-related protein and an uncharacterized integral membrane protein. A transition in Rmet_1627 led to an R->H exchange in a response regulator. The respective gene is in an operon Op0454r_1 with the genes encoding a histidine kinase and a drug resistance transporter of the EmrB/QacA subfamily downstream. This mutation may change resistance of *C. metallidurans* to an unknown component. A second transition changed an Ala for a Thr in Rmet_3389 or *gspL* in operon Op0954f_1 for a component of a type II secretion pathway. The third SNP in the Δ*zur* strain concerned an *in-frame* deletion of the five amino acids KAVDF at amino acid position 208 of Rmet_3488 for an extracellular ligand-binding receptor of an unknown ABC-type uptake system that is not encoded in the vicinity of Rmet_3488. The *Rmet_3488* gene is in the same region Op0970 as the *atp* operon, but is in its own operon with its own transcriptional start site 400 bp upstream of the gene. The SNPs in the Δ*zur* strain could not be connected to a phenotype that would compensate in any way increased zinc uptake capacity by an over-production of the zinc importer ZupT, or increased zinc handling capacity by CobW1, CobW2 or CobW3.

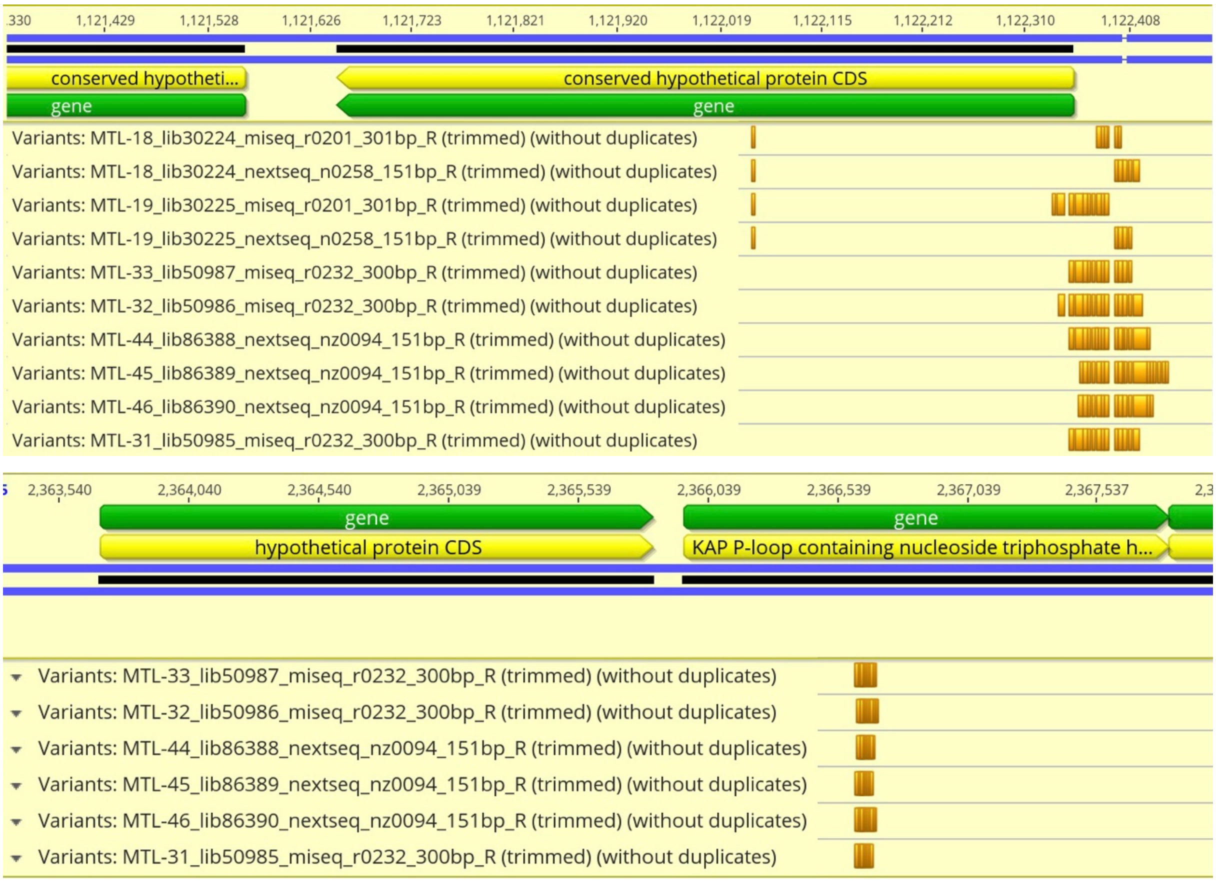

Supplementary Figure S13. Hypervariable regions in *slyB* and Rmet_2164. The screenshot on top shows polymorphisms in the 5'-UTR and at the beginning of the open reading frame of Rmet_1033 for Δ*slyB*, which occurred in all single and double *folE* mutants and no other strain except the two Δ*cobW3* Δ*cobW1* and Δ*cobW3* Δ*cobW2* double mutants of strain AE104. These strains also exhibited a SNP, an insertion of another CAA codon (decoded as Gln) in a series of already 10 Gln codons CAA. The screenshot at the bottom show polymorphisms in all *folE* mutants in *Rmet_2164*, which did not occur in any other sequenced strain.

Additional deletion of *cobW2* encoding the zinc-storage GTPase from the Δ*zur* strain resulted in the type O deletion in the chromid and three SNPs on the chromosome (Table 1), all of which were in genes expressed under non-challenging conditions. A deletion of three base pairs (CGC) in the tandem repeat $(CGC)_5$ deleted a proline residue in Rmet_0314, which encodes a transport system belonging to the major facilitator superfamily. Another deletion of 16 bp caused a frame-shift mutation and premature translation termination in Rmet_2477 (or *livG1*) encoding one of the two ATP-binding proteins of an ABC-type importer for the branched-chain amino acids Leu, Ile, Val. This particular import system comprises two membrane-integral subunits LivM1, LivH1, two ATP-binding subunits LivF1 and LivG1, and the periplasmic binding protein LivK1, all encoded in one operon Op0688r_2 together with components of the TCA cycle and enzymes for Leu biosynthesis. Since *C. metallidurans* contains multiple paralogs of Liv-ABC uptake systems, the consequences of the 16 bp deletion in *livG1* should not be severe, even when a polar effect on the expression of the genes downstream of *livG1* is assumed.

The third SNP of the Δ*zur* Δ*cobW2* mutant was a transition and subsequently a G->E exchange in Rmet_3225 for a diguanylate cyclase with a GGDEF domain (54), in this protein with a GGEEF sequence. The 599 aa long predicted protein could be membrane-associated with a large periplasmic and cytoplasmic part, two transmembrane alpha-helices and a short cytoplasmic amino terminus. The SNP exchanged a Gly in the second transmembrane helix (not in the GGEEF sequence) to an Glu, which decreased the hydrophobicity of the protein, may cause mis-folding during translation, insertion into the membrane und subsequently formation of a biofilm by *C. metallidurans*.

The Δ*zupT* Δ*cobW2* mutant still contained region B. Its parent probably was similar to the Δ*zupT* mutant strain DN816 and additional loss of the zinc-storage protein led to stabilization of the presence of CMGI-3, which encodes the soluble hydrogenase and Calvin-cycle enzymes. Again, this links the nickel-dependent protein to zinc homeostasis. Strain Δ*zupT* Δ*cobW2* contains a unique type S deletion, which is related to the type O deletion in strain Δ*zur* Δ*cobW2* but does not include deletion of the *gig* genes or of *rpoQ*. Compared to the Δ*zupT* parent, no additional SNPs occurred in the Δ*zupT* Δ*cobW2* mutant (Table 1).

Deletion of *cobW3* in the Δ*zupT* strain results in the A, B, C, and E mutations as in most strains and additionally in a unique type T deletion, which is part of the deletion cluster L, T, U, and M. Additional deletion of *cobW2* leads to the related deletion type U, while a type L deletion was found in the CH34 triple *cobW1,2,3* mutant and type M in AE104 *cobW* double mutants. Deletions L and T initially overlap and result in removal of porin genes among others in the L region *Rmet_4208-4462,* except Tn*6049*. Deletion T is the largest deletion of this cluster of related mutations and comprises removal of the second part of the ancient czc_2 determinant and for two TonB-dependent siderophore receptors. Both genes are present in the type U deletion, which ends after the $czcR_2S_2$-$czcB_2$”-$czcA_2$ deletion. The deletions L, T, U, and M change the composition of the outer membrane proteome in a different way and with $czcR_2S_2$ of important regulators of transition metal homeostasis.

Strain Δ*zupT* Δ*cobW3* contained two almost certain and two possible mutations, all in open reading frames. The two possible mutations were a transversion in an expressed *pilQ* gene for a type II/III secretion system and a transition in a gene for an extracellular ligand-binding receptor for an ABC importer. The two almost certain mutations were on the chromid, an insertion in a low-level expressed *fliH* gene for a flagellar assembly protein and a unexpressed *pdxK* gene for a pyridoxine kinase. Additional interruption of *cobW2* in the double mutant results in just one additional possible mutation, a transversion in a gene for a putative uncharacterized protein. The SNPs were less conclusive than the different types of deletions in the Δ*zupT* Δ*cobW3* strains with or without interruption of *cobW2.*

<u>Folate biosynthesis.</u> In *C. metallidurans* and other bacteria, folate biosynthesis is initiated by a FolE_I-type GTP cyclohydrolase (48, 55). Since tetrahydrofolate in essential for biosynthesis of GTP, deletion of *folE_I* genes might lead to conditional lethal conditions. *C. metallidurans* possesses three FolE-type enzymes, the strictly Zn-dependent FolE_IA and the two Fe/Mn/Co-dependent FolE_IB1 and FolE_IB2 enzymes (56). A triple mutant could not be constructed (56) so that *C. metallidurans* most likely does not possess independent pathways for folate biosynthesis (57). Interestingly, *folE_IB2* is part of the *cobW1* operon, which is controlled by Zur and is only expressed under severe zinc starvation conditions (50, 51). This suggests that conditional lethal conditions might indeed exist in a Δ*folE_IA* Δ*folE_IB1* double mutant when cultivated in the presence of sufficient zinc supply. Because the respective second mutation could only be realized as an interruption, this indicated that conditional lethal conditions might have occurred during the strain construction and might have been compensated by a suppression.

Genomic characterization of the *folE* double mutants was done twice. First, the Δ*folE_IB1* Δ*folE_IA::I* strain ("::I", indicating an interruption by an *in-frame* insertion; genome sequence number 33) and its parents were characterized. In a second round using improved protocols for DNA preparation and sequencing, the Δ*folE_IA* Δ*folE_IB1::I* mutant (number 46) was analyzed (Table 1). While in the first characterization the ΔE1B1 single mutant contained only type A, B, C, and E deletions, the same strain exhibited an emerging type V deletion in the second experiment, performed later. The large type V deletion Δ*Rmet_5657-5717* lost the gene encoding the periplasmic PstS phosphate binding protein, the $copA_2B_2C_2D_2$ and $copE_2S_2$ operons and part of the *nim* operon for an incomplete transenvelope efflux system. The second Δ*folE_IA* parent possessed a type Q deletion in both genomic sequences, which was related to type V but started more downstream with Δ*Rmet_5739*, extended to Δ*Rmet_5754* and included deletion of *furB* for the second regulator of iron uptake in *C. metallidurans*. The type Q deletion ended at the same position as the shorter deletions type R and type N, which are present in CH34_10, and the two Δ*cobW3* Δ*cobW1* or Δ*cobW2* double mutants (Table 1). Additionally, the Δ*folE_IA* single mutant carried a type P deletion, which was similar to the type O and S deletions in strains with other mutations in Zur-regulated genes. The Δ*folE* double mutants exhibited the same deletion pattern as their direct parent with the exception of the presence of region Q in the Δ1AΔ1B1 strain. Loss of other genes involved in metal and phosphate homeostasis might represent suppressions that allow an adaptation to handling of zinc and even of the consequences to zinc allocation to the important zinc-dependent FolE_IA enzyme.

The first sequencing experiment identified 8 possible mutations in the ΔE1B1 single mutant, but only one was confirmed in the second. This mutation was also present in both determinations of the ΔE1A genome, indicating an almost certain mutation despite the variation

frequencies between 73% and 92% in the four genomic sequences of the two strains. The mutation was a deletion of 12 bps at a distance of 51 bps upstream of the *murI* gene *Rmet_2274* for a glutamate racemase important for synthesis of D-glutamate for the peptide cross link of the bacterial peptidoglycan (58). Two additional possible mutations in the ΔE1B1 mutant occurred in the region upstream of the transcriptional start site of Rmet_1033. Closer examination (Suppl. Fig. S13 top) revealed a region containing polymorphisms with low variant frequencies directly upstream and at the 5' start of this gene in all *folE* mutants and also in the two Δ*cobW3* Δ*cobW1* and Δ*cobW3* Δ*cobW2* double mutants, which additionally possessed a SNP, an insertion of an additional Gln residue in a series of already 10 of these amino acyl residues. *Rmet_1033* is *slyB* and encodes a stress-induced complexes with outer membrane proteins during lipopolysaccharide destabilization (59), for instance as a consequence of a disturbed metal cation homeostasis. The hypervariable regions upstream of *murI* and *slyB* (Suppl. Fig. S13) were similar to those in the *pitA* gene in the Δe6 mutant (Fig. 6).

The remaining 6 SNPs identified in the first genomic sequence 31 were a substitution in chromid gene *Rmet_4641* on a low expression level encoding an ATP-dependent endonuclease and 5 SNPs in *Rmet_2164*. Again, careful analysis revealed that these changes were in a hypervariable region that was present in all other Δ*folE* mutants, albeit with low variant frequencies in some of these strains, but in no other sequenced strains (Supp. Fig. S13 bottom). Rmet_2164 encodes a KAP-P-loop NTPase (60), which may reorganize membrane-associated complexes in an NTP-dependent manner. No polymorphisms in addition to that upstream of *murI* was revealed in the second genomic sequence 44Δ1B1 (Table 1). Both hypervariable regions in all Δ*folE* mutants plus the *murI* mutation in the Δ1B1 mutant seemed to be involved in the cell wall-associated stress response.

In the two genomic sequences 32 and 45 of the Δ1A mutant, 2 mutations and 6 possible mutations were identified, the latter, however, were not identical (Table 1). The two mutations represented a transition leading to a D14->G14 exchange in the encoded UvrB protein of the nucleotide excision UvrABC system involved in DNA repair in general and also in the Mfd-mediated transcription-coupled DNA repair (61, 62). UvrB is an ATP-dependent exonuclease that binds to the UvrA homodimer and which scans DNA for damage. The mutation is located upstream of the N-terminal ATP-binding site in a region not conserved in the UvrD protein of *Bacillus subtilis* (63). AlphaFold3 models a slightly different structure of the ATP-binding wild type and mutant proteins (Suppl. Fig. S14) but the N-terminal disordered structure and the first alpha helix that contains the mutated region are not different. Any influence of the mutation on ATP-binding could not be derived from these structures. The second mutation revealed by both Δ1A genomic sequences were in *Rmet_0804*, a gene encoding a member of an uncharacterized prokaryotic family of stomatin-like proteins which is not usually expressed.

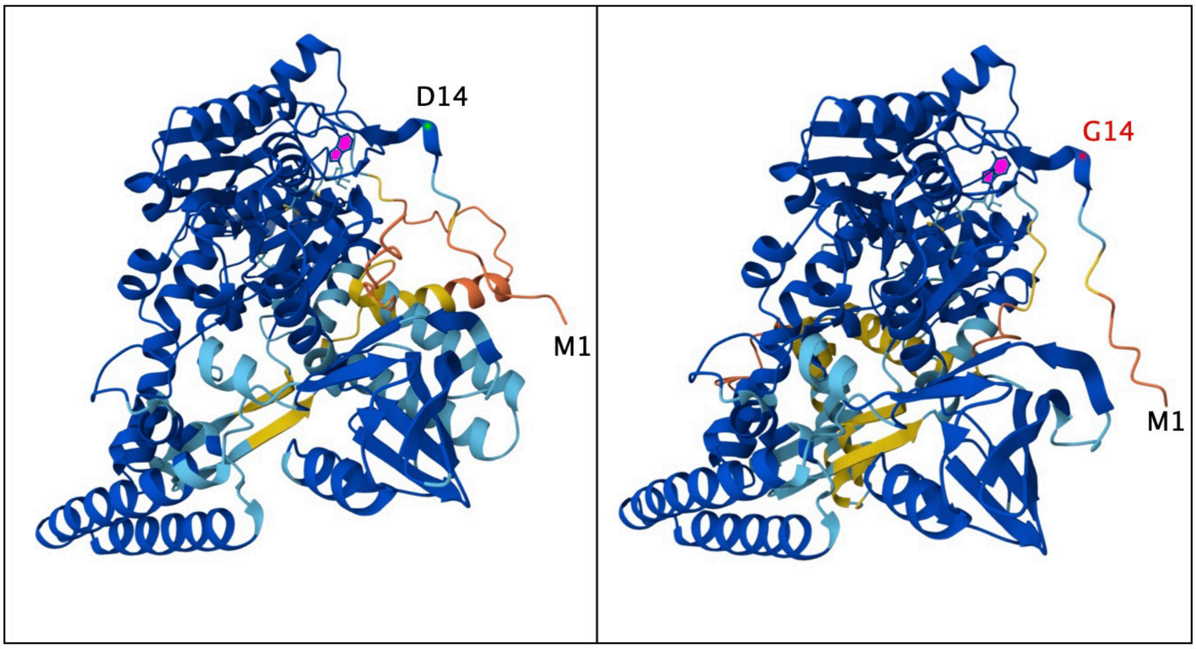

Supplementary Figure S14. Structure of UvrB wild type and its D14G mutant. Modeled with AlphaFold3 (17). The D14 in the wild type protein is marked by a green spot, the G14 by a red one. The adenine of the bound ATP is shown in purple.

The four possible chromosomal and two chromid mutation found in the first genomic sequence 32 of mutant Δ1A comprised mutations in *Rmet_2164*, *Rmet_4641* and an unexpressed putative uncharacterized protein, two changes upstream of *Rmet_1033/slyB* and one upstream of *Rmet_2274/murI*, all similar to mutations found in the first genomic sequence 31 of the sister strain Δ1B1. The six possible chromosomal polymorphisms found in the second genomic sequence 45 of the Δ1A strain were three SNPs within *Rmet_1033/slyB*, one SNP upstream of *Rmet_2274/murI* and two SNPs leading to substitution in genes for an extracellular ligand-binding receptor and a phosphoenolpyruvate protein phosphotransferase. All Δ*folE* single mutants exhibited the mutation in the region upstream of *murI*, the hypervariable regions upstream of *slyB* and the gene for the KAP-P-loop NTPase. Additionally, deletion in Δ*folE_IA* had a coincidence with a mutation in the UvrB gene which may have no physiological effect. Together, loss of FolE function seemed to be associated with cell envelope stress.

The two double mutants exhibited 5 possible chromosomal mutations in 33Δ1B1Δ1A plus in 46Δ1AΔ1B1 three almost certain and also 5 possible mutations (Table 1), the latter, however, is different from those found in the Δ1B1Δ1A mutant. One of the possible mutations in the Δ1B1Δ1A mutant resulted in a SFY->SEL mutation in a fimbrial biosynthesis outer membrane usher protein, a second is located in the promoter region of *agrC* 61 bp upstream of this gene with the transcriptional start site 138 bp upstream. The *agrCBA* operon encodes a silver-inducible RND-drive transenvelope system and the adjacent *agrRS* genes for a two-component regulatory system that interacts with regulation of metal resistance in *C. metallidurans* (64). The mutations in the other double mutant Δ1AΔ1B1 concern a mutation

between the gene encoding a molybdopterin-guanine dinucleotide biosynthesis protein and its transcriptional start site and two transitions leading to substitutions in a gene encoding an amino acid permease-associated region and a transporter of the major facilitator superfamily, respectively. Two of the five possible mutations are polymorphisms in the region between a gene for a malate dehydrogenase and an aldolase, another one in the upstream region of an integrase gene and yet another one resulted in a deletion of the four amino acids QRER in the RNAse E.

Features of the large deletions

Deletions on the chromosome:

A: ∆CMGI-2 ∆*Rmet_1236-1351* (A', deletion incomplete and starts at *Rmet_1253*). Genomic island-related *tra, trb, rep, par* and *int* genes; operon for the synthesis of the membrane-bound Ni-containing hydrogenase; toluene degradation gene cluster. It is present in all strains except CH34 wild type, AE126(pMOL28), but is emerging in AE126_10, emerging in AE104, but complete in AE104_10 and AE104_5, except in CH34 ∆*rpoE,P, cnrH::*interrupted, and is not deleted in ∆*zur* and ∆*zur* ∆*cobW2::*interrupted.

B: ∆CMGI-3. Deletion of the central part from *Rmet_1492 = cbbA1* to *Rmet_1541 = hoxX.* Deletion of all the genes encoding the Calvin cycle enzymes and those for the synthesis and maturation of the Ni-containing, soluble and NAD^+-reducing hydrogenase. It is present in all mutants except CH34 wild type; incomplete in AE126, but present in AE126_10, the AE104 parent and AE104_5, but in AE104_10*,* CH34 ∆*rpoE,P, cnrH::*interrupted, emerging in AE104∆*zupT*, and not present in AE104 ∆*zupT* ∆*cobW2::*interrupted.

E: ∆CMGI-4 except Tn*6048*. ∆*Rmet_6571/2987 - Rmet_3045* but not from *Rmet_2999* to *Rmet_3010*. Genes for methylene-THF reductase, the methionine biosynthesis regulator, MetR, an ABC-type importer for phosphonate in the first part of the deletion, silent and interrupted *hmzBA-hmzRS* genes for an HME-RND and its membrane fusion protein plus its two-component regulatory system, a cytochrome and a ferric reductase-like membrane protein. It does not occur in CH34 derivatives with both plasmids, emerging in AE126_10 and in all AE104 derivatives except the parent (although in AE104_10 and AE104_5), ∆*zur* and ∆*zur* ∆*cobW2::*interrupted.

G: ∆*Rmet*_6477, gene for a hypothetical exported protein with an uninterrupted stretch of 50 Leu residues. The open reading frame overlaps partly with that of *Rmet_1797* on the other

DNA strand. The region is not entirely deleted, but is a region of low coverage (5 to 18 runs). Together with deletion H only in strain AE104Δ9.

H: Δ*Rmet_2162_2161* for putative uncharacterized proteins. Together with deletion G only found in strain AE104Δ9.

J: ΔRmet_6403_0032. Deletion of a gene encoding a hypothetical protein and the outer membrane protein Rmet_0032. It is present in all mutants with multiple deletions in efflux systems.

<u>Deletions on plasmid pMOL28:</u>
D: Deletion Δ*Rmet_6189-6244* from downstream *tnpA* (Rmet_6188, Tn*4378*) to downstream of reverse gene Rmet_6245, including *chr* and *cnr* determinants. Presents a round colony morphology, but only in strain CH34_1.

<u>Deletions on the chromid</u>:
C: Δ*Rmet_5543-5544*, *caiB* for a carnitine dehydratase and *Rmet_2193* for a Zn-containing aldehyde dehydrogenase. Present in nearly all mutants. Exceptions are CH34 wild type, CH34 Δ*rpoIJK* (iron-connected ECF sigma factors) and it is not fully deleted in all cells of strain AE126(pMOL28).

F: Δ*Rmet_5535-5536* for another carnitine dehydratase and a putative uncharacterized protein. Present in AE104 Δ7 and Δ9.

I: Δ*Rmet_5561_6747_5562_5563* for a regulator and a H-NS-type DNA-binding protein that silences foreign DNA. In AE104 Δ9 and in CH34_10, which indicates the wild type was maintained 10 years on TMM without selection for metal resistance.

Deletion cluster V, R, N, and Q: in *folE* and *cobW* mutants and in CH34_10.

V: Δ*Rmet_5657-5717*. Gene for the periplasmic phosphate-binding protein PstS, the complete $copA_2B_2C_2D_2$ and $copE_2S_2$ operons, *nimC* outer membrane factor and the 3' part of the interrupted *nimA* for an HME-RND protein in the part not deleted in deletions N, Q, and R. Starts to appear in AE104 Δ*folE_IB1*.

R: Δ*Rmet_5681-5754*; in the part not deleted in deletions N and Q pilus assembly genes and those for the 5' part of the interrupted $nimA_2$ protein for an HME-RND protein plus *nimB* for its membrane fusion protein. The interrupted $nimBA_1$::A_2C genes are expressed. It is present in CH34 after 10 years on TMM without selection for metal resistance.

N: Δ*Rmet_5689-5754* encoding for an aquaporin channel protein and regulators in the part overlapping with deletions V and R. In the part overlapping only with deletion R the gene for porin *Rmet_5721*, some transport and capsule polysaccharide biosynthesis protein. In AE104 Δ*cobW3* mutants with a second deletion on one of the other *cobW* genes. Comes together with deletion M.

Q: Δ*Rmet_5739-5754* includes *Rmet_5746* = *furB* and other regulators. In AE104 Δ*folE_IA1*.

Deletion cluster P, K, S and O. Appear in constructed mutants with deletions in efflux systems and Zur regulon components.

All deletions are starting with Δ*Rmet_4603* downstream of *zntA-czcI2C2B2'-tnpA-sulP-uspA11-dksA* (*Rmet_4594*-4602) for the first part of the interrupted and translocated *zntA-czcICBA-RS* region, which is highly related to the *czc* region on plasmid pMOL30.

P: Δ*Rmet_4603-4616;* TonB-dependent copper receptor OprC and methionine biosynthesis protein MetY for the O-acetylhomoserine/-serine sulfhydrylase. In strains with a deletion of Δ*folE_IA1* but not in a double mutant that was constructed from a *folE_IB1* deletion strain.

K: Δ*Rmet_4603-4618.* Deletion P plus *piuA* for a TonB-dependent siderophore receptor. Appears in all strains with multiple deletions in genes encoding efflux systems.

S: Δ*Rmet_4603-4651.* Deletion K plus a Nod-like RND-driven transenvelope system, a sulfate transporter and a Zn-containing alcohol dehydrogenase. Appears in AE104 Δ*zupT* Δ*cobW2::*interrupted.

O: Δ*Rmet_4603-4693*. Deletion S plus *rpoD2*, a cluster for a silent third and membrane-bound hydrogenase, but without maturation factors, the *gig* genes and those for the sigma factor RpoQ. Appears in AE104 Δ*zur* Δ*cobW2::*interrupted.

Deletion cluster L, T, U and M: appears on strains with constructed mutations in the members of the Zur regulon.

L and T overlap; deletion L: Chromid Δ*Rmet_4208-4462* except Tn*6049*. Present in CH34 strain with all *cobW* genes deleted. In an overlap with deletion T, deletion of the genes for porins Rmet_4221, Rmet_4239, Rmet_4344, TCA cycle enzymes, NADH dehydrogenase and cytochrome *c* oxidase proteins, Zn-dependent alcohol dehydrogenases, transport and regulatory proteins before deletion U begins.

U, L and T overlap; deletion U: Δ*Rmet_4370-4469* except Tn*6049* and Tn*6050*. Appears in AE104 Δ*zupT* Δ*cobW3* Δ*cobW2::*interrupted. Enzymes involved in outer membrane biosynthesis, transport proteins, a *caiB* paralog and regulators before deletion M begins.

M, U, L and T overlap; deletion M: Δ*Rmet_4434-4469*. Present on two AE104 strains with a Δ*cobW3* deletion and deletion of one of the remaining *cobW* genes. Another *caiB* and *hns* paralog, respectively, before deletion L ends.

M, U and T overlap: The second part of the ancient $czcR_2S_2$-$czcB_2$”-$czcA_2$ cluster before deletions U and M end.

T: Δ*Rmet_4208-4497* except Tn*6049* and Tn*6050*. Appears in AE104 Δ*zupT* Δ*cobW3*. Comprises the deletions L, U and M including deletion of the second part of the ancient czc_2 cluster. After the overlap has ended within $czcB_2$”, the multiplied *dksA, uspA11* and *sulP* genes accompanying the interruption and relocation of the czc_2 region. Additionally, deletion of genes encoding an ABC-importer for branched-chain amino acids and the genes for the TonB-dependent siderophore receptors FfcA2 and FfcA1. The deletion ends before the genes for the sigma factor RpoJ and its regulator, which were not deleted. RpoJ is together with its paralogs RpoI and RpoK as FecI-like ECF sigma factor involved in mollifying the effect of the “iron-first” rule of *C. metallidurans* on the metabolism of other transition metal cations.

Other Supplementary Material

Supplementary Table S1. Changes in the proteome of ∆folE mutants[a].

Locus Tag	Gene	WT	Q (D)	Protein, Description
**UPREGULATION**				
**∆1A∆1B1 double mutant**				
Rmet_1103	*allB*	0	5815 (8)	Q1LPD7 Dihydroorotase
Rmet_1102		0	5367 (6)	Q1LPD8 Carbonic anhydrases
Rmet_4521		0	4295 (6)	Q1LEP0 Transcriptional regulator, Crp/Fnr family
Rmet_1099	*folE_IB2*	6±1	908 (5)	Q1LPE1 UPF0343 protein Rmet_1099
Rmet_1098	*cobW1*	28±7	194 (9)	Q1LPE2 Cobalamin synthesis protein, P47K
Rmet_1100	*cysS*	29±2	175 (13)	Q1LPE0 Cysteinyl-tRNA synthetase
Rmet_1101		57±19	74.5 (6.2)	Q1LPD9 6-pyruvoyl-tetrahydropterin synthase-like protein
Rmet_2787	*btuD*	520±38	14.5 (34.0)	Q1LJL6 ABC transporter-related protein
Rmet_3429	*yraO*	479±222	14.13 (5.18)	Q1LHS5 Phosphoheptose isomerase
Rmet_3492		598±45	11.93 (1.00)	Q1LHL2 Alpha/beta hydrolase fold-3
Rmet_0123		437±166	10.96 (2.75)	Q1LS67 TonB-dependent receptor
Rmet_0125	*cobW3*	843±46	3.91 (3.44)	Q1LS65 Cobalamin synthesis CobW-like protein
Rmet_0126	*dksA*	1359±340	*2.01 (0.71)*	Q1LS64 Transcriptional regulators, TraR/DksA family
Rmet_0127	*cobW2*	1541±404	2.61 (1.99)	Q1LS63 Cobalamin synthesis protein, P47K
**∆1A single mutant**				
Rmet_1439	*uppS*	0	3843 (4)	Q1LNF4 Undecaprenyl pyrophosphate synthetase
Rmet_1608		44±33	191 (1)	Q1LMY5 FAD linked oxidase-like protein
Rmet_3481	*gcvH*	150±121	19.2 (1.2)	Q1LHM3 Glycine cleavage system H protein
Rmet_2490		415±58	15.2 (8.9)	Q1LKF9 HpcH/HpaI aldolase
Rmet_0697	*pilA*	268±93	13.1 (6.0)	Q1LQJ3 Pilus assembly protein major pilin PilA
Rmet_0121		435±113	13.1 (2.0)	Q1LS69 Beta-lactamase-like protein
Rmet_2114		516±271	12.4 (1.0)	Q1LLI3 Putative uncharacterized protein
Rmet_2190		543±528	12.0 (6.0)	Q1LLA7 Putative uncharacterized protein
Rmet_4636		503±453	11.9 (6.3)	Q1LEC8 Putative uncharacterized protein

Rmet_0157		642±194	9.66 (1.05)	Q1LS33 Putative uncharacterized protein
Rmet_1099	*folE_IB2*	6±1	7.17 (1.35)	Q1LPE1 UPF0343 protein Rmet_1099
Rmet_0128	*zur*	2080±1224	*2.12 (0.94)*	Q1LS62 Putative ferric uptake regulator, FUR family
**Δ1B1 single mutant**				
Rmet_4521		0	2156 (4)	Q1LEP0 Transcriptional regulator, Crp/Fnr family
Rmet_3481	*gcvH*	150±121	24.2 (1.0)	Q1LHM3 Glycine cleavage system H protein
Rmet_3989		384±485	12.7 (1.1)	Q1LG69 Putative uncharacterized protein
Rmet_1872		359±50	10.5 (2.5)	Q1LM75 Diguanylate cyclase (GGDEF domain)
Rmet_2946	*secD*	476±188	8.2 (1.0)	Q1LJ57 Protein-export membrane protein SecD
Rmet_2063		360±36	7.1 (2.1)	Q1LLN4 Rhodanese-like protein
Rmet_3090	*pehR*	373±64	6.7 (4.8)	Q1LIR4 Transcriptional regulator, Fis family
Rmet_0697	*pilA*	268±93	6.4 (4.6)	Q1LQJ3 Pilus assembly protein major pilin PilA
Rmet_4307		488±661	*5.9 (0.8)*	Q1LFA3 Transcriptional regulator, TetR family
Rmet_1608		44±33	*5.66 (0.51)*	Q1LMY5 FAD linked oxidase-like protein
**DOWNREGULATION**				
**Δ1AΔ1B1 double mutant**				
Rmet_3689	*cheA*	2686±63	0.01 (37.01)	Q1LH15 CheA signal transduction histidine kinases
Rmet_3971		2434±399	0.06 (5.42)	Q1LG87 MCP methyltransferase, CheR-type
Rmet_4606	*metY*	1212±253	0.07 (3.92)	Q1LEF8 O-acetylhomoserine/O-acetylserine sulfhydrylase
Rmet_0355	*otsB*	2934±226	0.08 (8.36)	Q1LRI5 HAD-superfamily hydrolase subfamily IIB
Rmet_1684	*rbbA*	3162±429	0.09 (6.09)	Q1LMR1 Response regulator receiver domain protein (CheY-like)
Rmet_5311		4289±2210	0.10 (1.67)	Q1LCF6 Isochorismatase hydrolase
Rmet_5323	*zniR*	5121±257	0.11 (14.23)	Q1LCE4 Two component transcriptional regulator, LuxR family
Rmet_0779		2887±370	0.12 (5.44)	Q1LQB1 Maf-like protein Rmet_0779
Rmet_1023		3857±492	0.14 (6.42)	Q1LPL7 Putative site-specific recombinase transmembrane protein
Rmet_2910		3047±338	0.14 (4.20)	Q1LJ93 GCN5-related N-acetyltransferase
Rmet_5935		1022±314	0.14 (2.30)	Q1LAN2 Methyl-accepting chemotaxis sensory transducer
Rmet_0128	*zur*	2080±1224	*0.38 (0.96)*	Q1LS62 Putative ferric uptake regulator, FUR family
**Δ1A single mutant**				
Rmet_1101		57±19	0.02 (2.86)	Q1LPD9 6-pyruvoyl-tetrahydropterin synthase-like protein

Rmet_3488		4103±373	0.04 (8.07)	Q1LHL6 Extracellular ligand-binding receptor
Rmet_5513		4642±293	0.05 (10.78)	Q1LBV4 Transcriptional regulator, AraC family
Rmet_5323	*zniR*	5121±257	0.13 (13.68)	Q1LCE4 Two component transcriptional regulator, LuxR family
Rmet_1231	*kup*	1775±147	0.13 (8.48)	Q1LP09 Probable potassium transport system protein kup
Rmet_5743		2841±486	0.14 (4.75)	Q1LB74 AMP-dependent synthetase and ligase
Rmet_0246	*ptsI*	2683±211	0.16 (6.33)	Q1LRU4 Phosphoenolpyruvate--protein phosphotransferase
Rmet_2593		3141±231	0.17 (7.85)	Q1LK56 Glycosyl transferase, family 28
Rmet_4606	*metY*	1212±253	0.18 (3.48)	Q1LEF8 O-acetylhomoserine/O-acetylserine sulfhydrylase
Rmet_5667		4183±1248	0.18 (2.32)	Q1LBF0 Putative uncharacterized protein
**∆1B1 single mutant**				
Rmet_5513		4642±293	0.02 (14.82)	Q1LBV4 Transcriptional regulator, AraC family
Rmet_1821	*ybdG*	3011±348	0.05 (6.01)	Q1LMC6 MscS Mechanosensitive ion channel
Rmet_1684	*rbbA*	3162±429	0.06 (6.36)	Q1LMR1 Response regulator receiver domain protein (CheY-like)
Rmet_2027	*emrA*	4103±71	0.09 (29.52)	Q1LLS0 Secretion protein HlyD
Rmet_2614	*folE_IB1*	2834±350	0.13 (5.70)	Q1LK35 UPF0343 protein Rmet_2614
Rmet_1479		1992±282	0.14 (5.30)	Q1LNB4 Putative uncharacterized protein
Rmet_5323	*zniR*	5121±257	0.15 (10.74)	Q1LCE4 Two component transcriptional regulator, LuxR family
Rmet_0779		2887±370	0.15 (3.98)	Q1LQB1 Maf-like protein Rmet_0779
Rmet_1113		2091±357	0.17 (3.77)	Q1LPC7 IucA/IucC
Rmet_0355	*otsB*	2934±226	0.19 (6.19)	Q1LRI5 HAD-superfamily hydrolase subfamily IIB
Rmet_0128	*zur*	2080±1224	*0.50 (0.46)*	Q1LS62 Putative ferric uptake regulator, FUR family

[a]For each mutant, the ten proteins with the highest up- or down-regulation is listed. Three biological repeats, protein number in the parent strain AE104 indicated ± deviations. The quotient Q mutant/parent is indicated plus in paranthesises the D number, which is the quotient of the absolute distance of two data points divided by the sum of both deviations. In the double mutant, shaded fields indicate members of the Zur regulon. CobW2, DksA, CobW3 and the TonB-dependent protein were added, as FolE_IB1 in the ∆*folE_IA* mutant. Zur is listed for all three mutants. FolE_IA, Rmet_0124 and ZupT were not found.

Supplementary Table S2. Bacterial strains.

Strain	Description	Reference
CH34	Wild type	(65)
CH34_1	Coccoid form	(3)
CH34_10	10 years on plate	This publication
AE126	Only pMOL28	(65)
AE126_10	10 years on plate	This publication
AE104	Plasmid-free	(65)
AE104_5	Sensitive to Cd(II)	This publication
AE104_10	10 years on plate	(8) as “AE104_1”
Sigma factor mutants		
DN555	*ΔrpoE,P, cnrH::I*	(8)
DN554	*ΔrpoO,L, M::I*	(8)
DN553	*ΔrpoIJK*	(8)
DN546	*ΔrpoQR*	(8)
Uptake systems		
DN515	AE104Δ*zupT*	(66)
DN515_1	Long on plate	(3)
DN816	AE104ΔzupTAD	(3)
DN895	AE104Δ*feoB::I*	This publication
DN681	AE104Δ7	(18)
DN784	AE104Δ9	(19)
Efflux systems		
DN578	AE104Δe4	(34)
DN579	AE104Δe4z	(34)
DNA73	Δe5 (Δe4 Δ*cdfX*)	(42)
DNA75	Δe6 (Δe5 Δ*atmA*)	This publication
Zur regulon		
DN832	CH34ΔW1Δ3,2::I	(50)
DN837	AE104ΔW3, 1::I	(50)
DN838	AE104ΔW3, 2::I	(50)
DN728	AE104Δ*zur*	(52)
DN817	AE104Δ*zur*,W2::I	(50)
DN718	Δ*zupT* Δ*cobW2::I*	(50)
DN824	Δ*zupT* Δ*cobW3*	(50)
DN836	Δ*zupT* ΔW3ΔW2::I	(50)
Folate biosynthesis		
DN956	AE104ΔE1B1	(56)
DN957	AE104ΔE1A	(56)
DN967	AE104ΔE1B1ΔA::I	(56)
DN968	AE104ΔE1A1B1::I	(56)
folE_IB2-lacZ fusion in strain:		
	AE104	This publication
	AE104Δ*zupT*	This publication
	AE104Δ*zur*	This publication
	AE104Δ*folE_1A*	This publication
Plasmids		
	pVDZ’2	(21)
	pDNA130	(22)
	pVDZ’2(*lacZ*)	(67)
	pVDZ’2(*rpoHp-lacZ*)	This publication
	pVDZ’2(*rpoH$_{515}$p-lacZ*)	This publication

Supplementary Table S3. Primers.

Primer	5´→ 3´sequence
Rmet_5890 PstI Dis 5´	AAA CTG CAG CAC CGC GCA GTT CGA GGA C
Rmet_5890 XbaI Dis 5	AAA TCT AGA GGC CCG CGC TCA TCA TCA G
amtA cre-lox Mun	AAA CAA TTG GAC GAT GTC GGC ATA GAA GCT
amtA cre-lox Not	AAA GCG GCC GCA CGT TAG AAT TCG GGC CAA TG
atmA cre-lox Apa	AAA GGG CCC GCA AGG CAT CGA TTG TCG ACA
atmA cre-lox Age	AAA ACC GGT AAG CGC CAG TCG AGC TGA AAG
lacZ_fus_1099_Xba	AAA TCT AGA TCA TGC GGC CAC CTC CC
lacZ_fus_1099_Pst	AAA CTG CAG AGT GAG GCG TCG ATCA C
rpoH Promo836upBamHI	AAA GGA TCC AGG CGA CAT CAG GCT ATC CA
rpoH Promo938downBam	AAA GGA TCC GAA GGG ACA CCG CAT TGC AA

Supplementary Excel Files

1. Overview mutations
2. Transcriptional Landscape
3. Proteomics

Literature of the Supplement

1. Janssen PJ, Van Houdt R, Moors H, Monsieurs P, Morin N, Michaux A, Benotmane MA, Leys N, Vallaeys T, Lapidus A, Monchy S, Medigue C, Taghavi S, McCorkle S, Dunn J, van der Lelie D, Mergeay M. 2010. The complete genome sequence of *Cupriavidus metallidurans* strain CH34, a master survivalist in harsh and anthropogenic environments. PLoS One 5:e10433.
2. Altschul SF, Madden TL, Schaffer AA, Zhang J, Zhang Z, Miller W, Lipman DJ. 1997. Gapped BLAST and PSI-BLAST: a new generation of protein database search programs. Nucl Acid Res 25:3389–3402.
3. Große C, Kohl T, Herzberg M, Nies DH. 2022. Loss of mobile genomic islands in metal resistant, hydrogen-oxidizing *Cupriavidus metallidurans*. Appl Environ Microbiol 88:e02048–21.
4. Van Houdt R, Monchy S, Leys N, Mergeay M. 2009. New mobile genetic elements in *Cupriavidus metallidurans* CH34, their possible roles and occurrence in other bacteria. Antonie Van Leeuwenhoek 96:205–226.
5. Große C, Grau J, Herzberg M, Nies DH. 2024. Antisense transcription is associated with expression of metal resistance determinants in *Cupriavidus metallidurans* CH34. Metallomics 16:mfae057.
6. Gilman MSA, Shlosman I, Guerra DDS, Domecillo M, Fivenson EM, Bourett C, Bernhardt TG, Polizzi NF, Loparo JJ, Kruse AC. 2025. Conformational regulation of two essential activators of bacterial cell elongation. Proc Natl Acad Sci U S A 122:e2514198122.
7. Nies DH, Schleuder G, Galea D, Herzberg M. 2024. A flow equilibrium of zinc in cells of *Cupriavidus metallidurans*. J Bacteriol 206:e00080–24.
8. Große C, Poehlein A, Blank K, Schwarzenberger C, Schleuder G, Herzberg M, Nies DH. 2019. The third pillar of metal homeostasis in *Cupriavidus metallidurans* CH34: Preferences are controlled by extracytoplasmic functions sigma factors. Metallomics 11:291–316.
9. Monchy S, Benotmane MA, Wattiez R, van Aelst S, Auquier V, Borremans B, Mergeay M, Taghavi S, van der Lelie D, Vallaeys T. 2006. Transcriptomics and proteomic analysis of the pMOL30-encoded copper resistance in *Cupriavidus metallidurans* strain CH34. Microbiology 152:1765–1776.
10. Bütof L, Wiesemann N, Herzberg M, Altzschner M, Holleitner A, Reith F, Nies DH. 2018. Synergetic gold-copper detoxification at the core of gold biomineralisation in *Cupriavidus metallidurans*. Metallomics 10:278–286.
11. Singh SK, Grass G, Rensing C, Montfort WR. 2004. Cuprous oxidase activity of CueO from *Escherichia coli*. J Bacteriol 186:7815–7817.
12. Roberts SA, Wildner GF, Grass G, Weichsel A, Ambrus A, Rensing C, Montfort WR. 2003. A labile regulatory copper ion lies near the T1 copper site in the multicopper oxidase CueO. J Biol Chem 278:31958–31963.
13. Roberts SA, Weichsel A, Grass G, Thakali K, Hazzard JT, Tollin G, Rensing C, Montfort WR. 2002. Crystal structure and electron transfer kinetics of CueO, a multicopper oxidase

required for copper homeostasis in *Escherichia coli*. Proc Natl Acad Sci U S A 99:2766–2771.
14. Grass G, Rensing C. 2001. CueO is a multi-copper oxidase that confers copper tolerance in *Escherichia coli*. Biochem Biophys Res Commun 286:902–908.
15. Hirth N, Gerlach MS, Wiesemann N, Herzberg M, Grosse C, Nies DH. 2023. Full copper resistance in *Cupriavidus metallidurans* requires the interplay of many resistance systems. Appl Environ Microbiol 89:e00567–23.
16. Roulling F, Godin A, Feller G. 2022. Function and versatile location of Met-rich inserts in blue oxidases involved in bacterial copper resistance. Biochimie 194:118–126.
17. Abramson J, Adler J, Dunger J, Evans R, Green T, Pritzel A, Ronneberger O, Willmore L, Ballard AJ, Bambrick J, Bodenstein SW, Evans DA, Hung C-C, O'Neill M, Reiman D, Tunyasuvunakool K, Wu Z, Žemgulytė A, Arvaniti E, Beattie C, Bertolli O, Bridgland A, Cherepanov A, Congreve M, Cowen-Rivers AI, Cowie A, Figurnov M, Fuchs FB, Gladman H, Jain R, Khan YA, Low CMR, Perlin K, Potapenko A, Savy P, Singh S, Stecula A, Thillaisundaram A, Tong C, Yakneen S, Zhong ED, Zielinski M, Žídek A, Bapst V, Kohli P, Jaderberg M, Hassabis D, Jumper JM. 2024. Accurate structure prediction of biomolecular interactions with AlphaFold 3. Nature 630:493–500.
18. Herzberg M, Bauer L, Kirsten A, Nies DH. 2016. Interplay between seven secondary metal transport systems is required for full metal resistance of *Cupriavidus metallidurans*. Metallomics 8:313–326.
19. Grosse C, Herzberg M, Schüttau M, Nies DH. 2016. Characterization of the ∆7 mutant of *Cupriavidus metallidurans* with deletions of seven secondary metal uptake systems. mSystems 1:e00004–16.
20. Herzberg M, Bauer L, Nies DH. 2014. Deletion of the *zupT* gene for a zinc importer influences zinc pools in *Cupriavidus metallidurans* CH34. Metallomics 6:421–436.
21. Deretic V, Chandrasekharappa S, Gill JF, Chatterjee DK, Chakrabarty A. 1987. A set of cassettes and improved vectors for genetic and biochemical characterization of *Pseudomonas* genes. Gene 57:61–72.
22. Nies DH, Nies A, Chu L, Silver S. 1989. Expression and nucleotide sequence of a plasmid-determined divalent cation efflux system from *Alcaligenes eutrophus.* Proc Natl Acad Sci U S A 86:7351–7355.
23. Große C, Grau J, Große I, Nies DH. 2022. Importance of RpoD- and non-RpoD-dependent expression of horizontally acquired genes in *Cupriavidus metallidurans*. Microbiol Spectr 10:e00121–22.
24. Nordin N, Guskov A, Phua T, Sahaf N, Xia Y, Lu SY, Eshaghi H, Eshaghi S. 2013. Exploring the structure and function of *Thermotoga maritime* CorA reveals the mechanism of gating and ion selectivity in Co^{2+}/Mg^{2+} transport. Biochem J 451:365–374.
25. Kumar S, Doerrler WT. 2014. Members of the conserved DedA family are likely membrane transporters and are required for drug resistance in *Escherichia coli*. Antimicrob Agents Chemother 58:923–30.
26. Bordo D, Bork P. 2002. The rhodanese/Cdc25 phosphatase superfamily. Sequence-structure-function relations. EMBO Rep 3:741–746.
27. Faehnle CR, Joshua-Tor L. 2007. Argonautes confront new small RNAs. Curr Opin Chem Biol 11:569–77.
28. Arold ST, Leonard PG, Parkinson GN, Ladbury JE. 2010. H-NS forms a superhelical protein scaffold for DNA condensation. Proc Natl Acad Sci U S A 107:15728–32.
29. Rangarajan AA, Schnetz K. 2018. Interference of transcription across H-NS binding sites and repression by H-NS. Mol Microbiol 108:226–239.
30. Winardhi Ricksen S, Yan J, Kenney Linda J. 2015. H-NS regulates gene expression and compacts the nucleoid: insights from single-molecule experiments. Biophys J 109:1321–1329.
31. Gao Y, Foo YH, Winardhi RS, Tang Q, Yan J, Kenney LJ. 2017. Charged residues in the H-NS linker drive DNA binding and gene silencing in single cells. Proc Natl Acad Sci U S A 114:12560–12565.
32. Navarre WW, Porwollik S, Wang YP, McClelland M, Rosen H, Libby SJ, Fang FC. 2006. Selective silencing of foreign DNA with low GC content by the H-NS protein in *Salmonella*. Science 313:236–238.
33. Shin M, Lagda AC, Lee JW, Bhat A, Rhee JH, Kim JS, Takeyasu K, Choy HE. 2012. Gene silencing by H-NS from distal DNA site. Mol Microbiol 86:707–19.

34. Scherer J, Nies DH. 2009. CzcP is a novel efflux system contributing to transition metal resistance in *Cupriavidus metallidurans* CH34. Mol Microbiol 73:601–621.
35. Legatzki A, Anton A, Grass G, Rensing C, Nies DH. 2003. Interplay of the Czc-system and two P-type ATPases in conferring metal resistance to *Ralstonia metallidurans*. J Bacteriol 185:4354–4361.
36. Grass G, Otto M, Fricke B, Haney CJ, Rensing C, Nies DH, Munkelt D. 2005. FieF (YiiP) from *Escherichia coli* mediates decreased cellular accumulation of iron and relieves iron stress. Arch Microbiol 183:9–18.
37. Munkelt D, Grass G, Nies DH. 2004. The chromosomally encoded cation diffusion facilitator proteins DmeF and FieF from *Wautersia metallidurans* CH34 are transporters of broad metal specificity. J Bacteriol 186:8036–8043.
38. Van Houdt R, Monsieurs P, Mijnendonckx K, Provoost A, Janssen A, Mergeay M, Leys N. 2012. Variation in genomic islands contribute to genome plasticity in *Cupriavidus metallidurans*. BMC Genomics 13:111.
39. von Rozycki T, Nies DH. 2009. *Cupriavidus metallidurans*: evolution of a metal-resistant bacterium. Antonie Van Leeuwenhoek 96:115–139.
40. Cuthbertson L, Nodwell JR. 2013. The TetR Family of Regulators. Microbiol Mol Biol Rev 77:440–475.
41. Le TB, Schumacher MA, Lawson DM, Brennan RG, Buttner MJ. 2011. The crystal structure of the TetR family transcriptional repressor SimR bound to DNA and the role of a flexible N-terminal extension in minor groove binding. Nucleic Acids Res 39:9433–47.
42. Schulz V, Galea D, Schleuder G, Strohmeyer P, Große C, Herzberg M, Nies DH. 2024. The efflux system CdfX exports zinc that cannot be transported by ZntA in *Cupriavidus metallidurans*. J Bacteriol 206:e00299–24.
43. Mikolay A, Nies DH. 2009. The ABC-transporter AtmA is involved in nickel and cobalt resistance of *Cupriavidus metallidurans* strain CH34 Antonie van Leeuwenhoek 96:183–191.
44. Blaby-Haas CE, Flood JA, de Crecy-Lagard V, Zamble DB. 2012. YeiR: a metal-binding GTPase from *Escherichia coli* involved in metal homeostasis. Metallomics 4:488–497.
45. Edmonds KA, Jordan MR, Giedroc DP. 2021. COG0523 proteins: a functionally diverse family of transition metal-regulated G3E P-loop GTP hydrolases from bacteria to man. Metallomics 13.
46. Pasquini M, Grosjean N, Hixson KK, Nicora CD, Yee EF, Lipton M, Blaby IK, Haley JD, Blaby-Haas CE. 2022. Zng1 is a GTP-dependent zinc transferase needed for activation of methionine aminopeptidase. Cell Rep 39:110834.
47. Weiss A, Murdoch CC, Edmonds KA, Jordan MR, Monteith AJ, Perera YR, Nassif AMR, Petoletti AM, Beavers WN, Munneke MJ, Drury SL, Krystofiak ES, Thalluri K, Wu HW, Kruse ARS, DiMarchi RD, Caprioli RM, Spraggins JM, Chazin WJ, Giedroc DP, Skaar EP. 2022. Zn-regulated GTPase metalloprotein activator 1 modulates vertebrate zinc homeostasis. Cell 185:2148–2163.
48. Chandrangsu P, Huang X, Gaballa A, Helmann JD. 2019. *Bacillus subtilis* FolE is sustained by the ZagA zinc metallochaperone and the alarmone ZTP under conditions of zinc deficiency. Mol Microbiol 112:751–765.
49. Galea D, Herzberg M, Nies DH. 2024. The metal-binding GTPases CobW2 and CobW3 are at the cross-road of zinc and cobalt homeostasis in *Cupriavidus metallidurans* J Bacteriol 206:e00226–24.
50. Bütof L, Große C, Lilie H, Herzberg M, Nies DH. 2019. Interplay between the Zur regulon components and metal resistance in *Cupriavidus metallidurans*. J Bacteriol 201:e00192–19.
51. Bütof L, Schmidt-Vogler C, Herzberg M, Große C, Nies DH. 2017. The components of the unique Zur regulon of *Cupriavidus metallidurans* mediate cytoplasmic zinc handling. J Bacteriol 199:e00372–17, spotlight article.
52. Schmidt C, Schwarzenberger C, Grosse C, Nies DH. 2014. FurC regulates expression of *zupT* for the central zinc importer ZupT of *Cupriavidus metallidurans*. J Bacteriol 196:3461–3471.
53. Mijnendonckx K, Ali MM, Provoost A, Janssen P, Mergeay M, Leys N, Charlier D, Monsieurs P, Van Houdt R. 2019. Spontaneous mutation in the AgrRS two-component regulatory system of *Cupriavidus metallidurans* results in enhanced silver resistance. Metallomics 11:1912–1924.

54. Ryjenkov DA, Tarutina M, Moskvin OV, Gomelsky M. 2005. Cyclic diguanylate is a ubiquitous signaling molecule in bacteria: Insights into biochemistry of the GGDEF protein domain. Journal of Bacteriology 187:1792–1798.
55. Nies DH. 2019. The ancient alarmone ZTP and zinc homeostasis in *Bacillus subtilis*. Mol Microbiol 112:741–746.
56. Schulz V, Galea D, Herzberg M, Nies DH. 2024. Protecting the Achilles heel: three FolE_I-type GTP-cyclohydrolases needed for full growth of metal resistant *Cupriavidus metallidurans* under a variety of conditions. J Bacteriol 206:e00395–23.
57. de Crécy-Lagard V. 2014. Variations in metabolic pathways create challenges for automated metabolic reconstructions: Examples from the tetrahydrofolate synthesis pathway. Comput Struct Biotechnol J 10:41–50.
58. Doublet P, van Heijenoort J, Bohin JP, Mengin-Lecreulx D. 1993. The *murI* gene of *Escherichia coli* is an essential gene that encodes a glutamate racemase activity. J Bacteriol 175:2970–9.
59. Janssens A, Nguyen VS, Cecil AJ, Van der Verren SE, Timmerman E, Deghelt M, Pak AJ, Collet JF, Impens F, Remaut H. 2024. SlyB encapsulates outer membrane proteins in stress-induced lipid nanodomains. Nature 626:617–625.
60. Aravind L, Iyer LM, Leipe DD, Koonin EV. 2004. A novel family of P-loop NTPases with an unusual phyletic distribution and transmembrane segments inserted within the NTPase domain. Genome Biol 5:R30.
61. Adebali O, Chiou YY, Hu JC, Sancar A, Selby CP. 2017. Genome-wide transcription-coupled repair in *Escherichia coli* is mediated by the Mfd translocase. Proc Natl Acad Sci USA 114:E2116–E2125.
62. Adebali O, Sancar A, Selby CP. 2017. Mfd translocase is necessary and sufficient for transcription-coupled repair in *Escherichia coli*. J Biol Chem 292:18386–18391.
63. Webster MP, Jukes R, Zamfir VS, Kay CW, Bagnéris C, Barrett T. 2012. Crystal structure of the UvrB dimer: insights into the nature and functioning of the UvrAB damage engagement and UvrB-DNA complexes. Nucleic Acids Res 40:8743–58.
64. Große C, Scherer J, Schleuder G, Nies DH. 2023. Interplay between two-component regulatory systems is involved in control of *Cupriavidus metallidurans* metal resistance genes J Bacteriol 205:e00343–22.
65. Mergeay M, Nies D, Schlegel HG, Gerits J, Charles P, van Gijsegem F. 1985. *Alcaligenes eutrophus* CH34 is a facultative chemolithotroph with plasmid-bound resistance to heavy metals. J Bacteriol 162:328–334.
66. Kirsten A, Herzberg M, Voigt A, Seravalli J, Grass G, Scherer J, Nies DH. 2011. Contributions of five secondary metal uptake systems to metal homeostasis of *Cupriavidus metallidurans* CH34. J Bacteriol 193:4652–4663.
67. Große C, Anton A, Hoffmann T, Franke S, Schleuder G, Nies DH. 2004. Identification of a regulatory pathway that controls the heavy metal resistance system Czc via promoter *czcNp* in *Ralstonia metallidurans*. Arch Microbiol 182:109–118.
